# Trifunctionalized Naphthalene Diimides and Dimeric Analogues as G-Quadruplex-Targeting Anticancer Agents Selected by Affinity Chromatography

**DOI:** 10.3390/ijms21061964

**Published:** 2020-03-13

**Authors:** Chiara Platella, Valentina Pirota, Domenica Musumeci, Federica Rizzi, Sara Iachettini, Pasquale Zizza, Annamaria Biroccio, Mauro Freccero, Daniela Montesarchio, Filippo Doria

**Affiliations:** 1 Department of Chemical Sciences, University of Naples Federico II, 80126 Naples, Italy; chiara.platella@unina.it (C.P.); domenica.musumeci@unina.it (D.M.); 2 Department of Chemistry, University of Pavia, 27100 Pavia, Italy; valentina.pirota@unipv.it (V.P.); f.rizzi@soton.ac.uk (F.R.); mauro.freccero@unipv.it (M.F.); 3 Oncogenomic and Epigenetic Unit, IRCCS-Regina Elena National Cancer Institute, 00144 Rome, Italy; sara.iachettini@ifo.gov.it (S.I.); Pasquale.zizza@ifo.gov.it (P.Z.); annamaria.biroccio@ifo.gov.it (A.B.)

**Keywords:** naphthalene diimide, G-quadruplex, G4-CPG assay, G-quadruplex-selective ligand, anticancer drug

## Abstract

A focused library of newly designed monomeric and dimeric naphthalene diimides (NDIs) was analyzed in its ability to recognize specific G-quadruplex (G4) structures discriminating duplex DNA. The best G4 ligands—according to an affinity chromatography-based screening method named G4-CPG—were tested on human cancer and healthy cells, inducing DNA damage at telomeres, and in parallel, showing selective antiproliferative activity on HeLa cancer cells with IC_50_ values in the low nanomolar range. CD and fluorescence spectroscopy studies allowed detailed investigation of the interaction in solution with different G4 and duplex DNA models of the most promising NDI of the series, as determined by combining the biophysical and biological assays’ data.

## 1. Introduction

DNA and RNA sequences potentially forming G-quadruplex (G4) structures are located in functional and highly conserved human genomic regions involved in cancer onset and progression, such as telomeres and oncogene promoters [1,2,3,4,5,6]. In the search for G4-selective ligands as novel anticancer drugs, naphthalene diimides (NDIs) emerged as promising compounds for their ability to interact with G-tetrads. Among the many advantages of NDIs, the most noteworthy are their chemical accessibility and possibility to easily functionalize their aromatic cores with multiple diverse pendant groups, thus allowing a fine modulation of their properties, and particularly of their affinity toward different secondary structure-forming oligonucleotides [7,8,9,10]. For these peculiar features, NDIs have been extensively studied both as fluorescent probes and oligonucleotide-targeting ligands [11,12].

Indeed, it has been shown that the substitution pattern of their core, as well as the chemical nature of their substituents, plays a crucial role in NDI G4 binding, as well as in G4 vs. duplex DNA selective recognition [8,13]. In this context, some of us recently synthesized a set of NDIs derivatized with alkylamine substituents on the naphthalene core [14,15]. These compounds exhibited excellent water solubility and cellular entry capability, thus emerging as versatile tools for both therapeutic and diagnostic applications [14,15]. A number of di-, tri- and tetrasubstituted monomeric NDIs were also designed and synthesized, showing telomerase inhibitory activity and antiproliferative effects on different cancer cell lines, as well as the ability to easily access the nucleus [11,12,13,16,17,18]. It is noteworthy that the trisubstituted NDIs proved to be the most cytotoxic compounds in the investigated series, being also the most selective ones on several cancer cell lines over healthy cells and displaying antitumor activity in vivo in human pancreatic ductal adenocarcinoma (PDAC) animal models [19].

The chemical diversity of this class of compounds has been further expanded with the design and synthesis of the first prototype of dimeric NDI, obtained by coupling two monomeric NDI units through a flexible heptyl linker [20]. Remarkably, this NDI had appealing properties for the detection and targeting of G4 structures in the context of in vivo applications [20]. In addition, it exhibited a good G4 vs. duplex DNA selectivity [20], thus proving to be a promising compound in the design of optimized G4-selective ligands. In this perspective, dimeric naphthalene diimides featuring different substitution patterns and linkers have been then synthesized, showing high activity on different cancer cell lines [21,22]. Notably, these compounds displayed a remarkable affinity for G4 DNA, but, unfortunately, they displayed a low ability to discriminate G4 vs. duplex DNA. This poor selectivity also affected their cytotoxic behavior [21,22]. In fact, the best dimeric NDIs as G4 binders showed high cytotoxicity toward cancer cells, with IC_50_ values in the nM range, but also produced moderate antiproliferative effects toward healthy cells [21].

**Figure 1:**
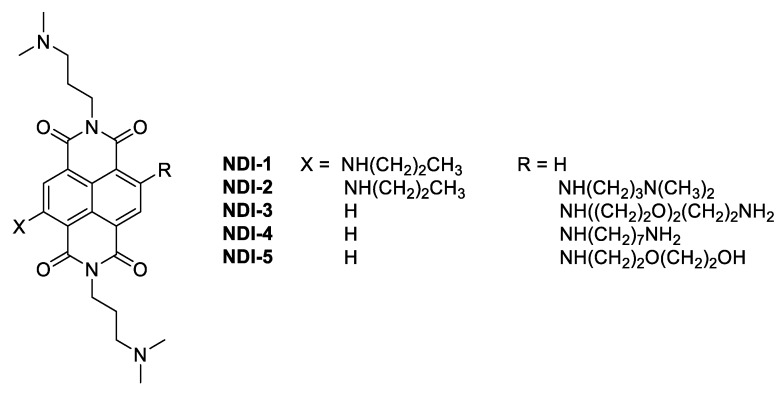
Chemical structures of the monomeric naphthalene diimides here investigated.

**Figure 2:**
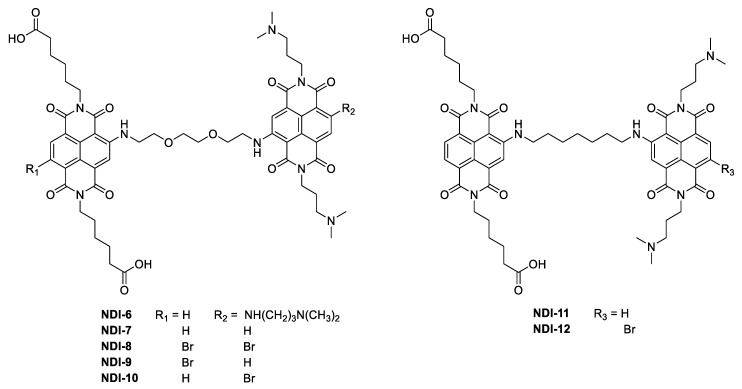
Chemical structures of the dimeric naphthalene diimides here investigated.

Stimulated by the intriguing anticancer properties of the G4-targeting NDIs and aiming at improving their selectivity, novel differently functionalized monomeric (Figure 1) and dimeric (Figure 2) NDIs were here designed, synthesized and evaluated. To enhance the recognition specificity toward the G4 structures, two major modifications were introduced in the NDI core. First of all, in the dimeric series, one of the NDI units was decorated with anionic side chains at the imide positions, and the other was decorated with protonable alkylamines, in order to reduce the net positive charge of the new hetero dyads at physiological pH. This design, with respect to the previously synthesized dimeric NDIs containing only alkylamines in their side chains [21], allows for the reduction of the unspecific interactions toward duplex DNA, typically resulting from the electrostatic attractions between the backbone phosphates and the protonated ligands. In this frame, the new NDIs should lose some affinity degrees toward the G4 targets but, in turn, gain enhanced G4 vs. duplex selectivity [23]. The second structural modification here introduced is related to the side chain (for the monomeric NDIs) or the linker connecting the two NDI units (for the dimeric NDIs). In the latter case, we chose to investigate linkers differing in nature and length, and particularly oligoethyleneglycol chains, following a widely exploited strategy in drug design [24], in parallel with alkyl chains. Although the benefit produced by oligoethyleneglycol chains on NDI solubility should be robust, intramolecular hydrogen bonding and/or cation coordination might induce additional folding and consequent negative steric effects between the two NDI units. This aspect justifies a systematic comparison of the selected oligoethyleneglycol vs. alkyl linkers [25].

This library of brand-new NDIs was then analyzed, to identify G4-selective ligands, using G4-functionalized Controlled Pore Glass solid supports in an affinity chromatography-based screening (G4-CPG assay) recently developed by some of us [26,27,28]. Next, the ligands featuring good affinity for the selected G4s, coupled with significant G4 vs. duplex DNA discrimination ability, were tested in their biological activity. Finally, the most promising compound, in terms of both selective targeting of G4 structures and anticancer activity, was investigated in detail in its interaction with cancer-related G4 and model-duplex structures by CD and fluorescence experiments.

Considering that even tiny chemical differences in the NDI substitution pattern can result in substantial differences in their DNA-binding properties and bioactivity, we here aim at adding a precious piece to the puzzle rationalizing the structure–activity relationships of functionalized NDIs, with particular focus on their interaction with G4/duplex DNA, as well as their cytotoxicity on cancer and normal cells.

## 2. Results and Discussion

### 2.1. Synthesis of the Functionalized Monomeric and Dimeric Naphthalene Diimides (**NDI** 1–12)

**Figure 3:**
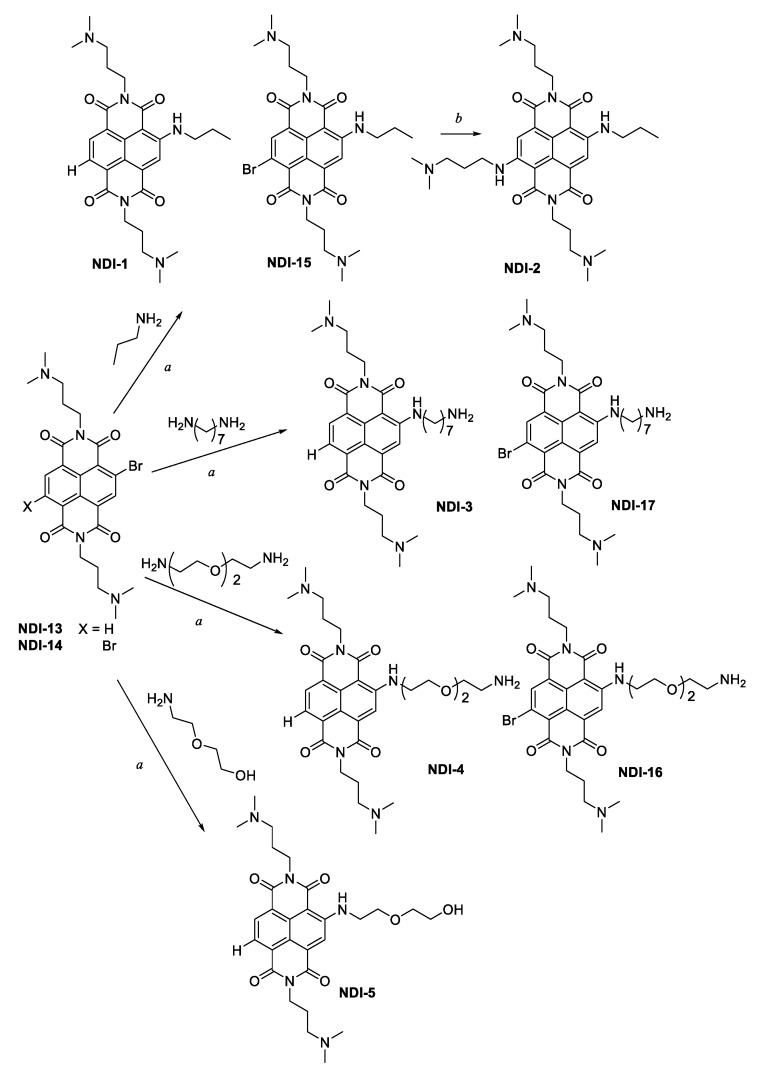
Synthesis of the monomeric NDIs: the letter *a* denotes 2.5 equivalents of the opportune amine in CH_3_CN, 75 °C, 2 h; the letter *b* denotes **NDI-15** was treated in neat *N*^1^,*N*^1^-dimethylpropane-1,3-diamine, 150 °C, 3 min, closed-vessel microwave-assisted.

**Figure 4:**
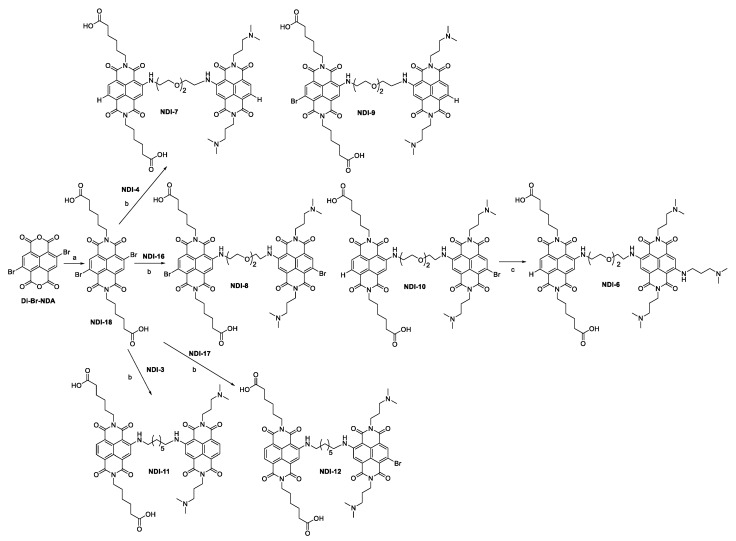
Synthesis of the dimeric NDIs **6**–**12**: letter *a* denotes 6-aminohexanoic acid (four equivalents), glacial acetic acid, 120 °C, 20 min, closed-vessel microwave-assisted; letter *b* denotes **NDI-18** (0.95 equivalent) with one equivalent of **3** or **4** or **16** or **17** and five equivalents of DIPEA in DMF, 110 °C, 20 min, closed-vessel MW-assisted; letter *c* denotes **NDI-10** in neat *N*^1^,*N*^1^-dimethylpropane-1,3-diamine, 180 °C, 5 min, closed-vessel microwave-assisted.

All the new molecular entities were prepared according to the synthetic protocols outlined in Figure 3 and Figure 4. We used a well-established method for the preparation of the mixture of mono- (**NDI-13**) and dibromo-substituted (**NDI-14**) *N*,*N’*-bis((dimethylamino)propylamino) NDIs, directly used for the first step, *a* (Figure 3) [15]. The nucleophilic aromatic substitution (S_N_Ar, step *a*) was carried out in the presence of an excess (2.5 equiv.) of different amines, using already published procedure [22]. In particular, n-propylamine was used to obtain **NDI-1** and **NDI-15**; 1,7-diaminoheptane to **NDI-3** and **NDI-17**, 2,2-(ethylenedioxy)bis(ethylamine) was used to yield **NDI-4** and **NDI-16** and 2-(2-aminoethoxy)ethanol to give **NDI-5**. All these compounds were further purified by preparative HPLC, yielding the corresponding trifluoroacetate salts, characterized by ESI-MS and NMR.

Monomeric **NDI-2** (Figure 3) was synthesized by a second, but harsher, microwave-assisted S_N_Ar (step *b*), dissolving pure **NDI-15** in neat *N^1^,N^1^*-dimethylpropane-1,3-diamine (150 °C, 200 psi, 200 W, 3 min, sealed reaction vessels), thus obtaining the target compound in almost quantitative yield [29].

On the other hand, to obtain **NDI-18** (Figure 4), one of the precursor units of dimeric NDI compounds, we used a previously reported synthetic procedure [30], which required quite harsh conditions to provide the target product in good yield. Starting from synthetic 2,6-dibromo-1,4,5,8-naphtalenetetracarboxylic bisanhydride (Di-Br-**NDA**) [31], four equivalents of 6-aminohexanoic acid were added in acetic acid, and the reaction was performed at 120 °C for 20 min, under microwave assistance.The product did not require a purification step after the work-up reaction.

In order to prepare dimeric NDIs **6**–**12** (Figure 4), we combined two different NDI units: one containing dimethylamino groups on the imide side chains, positively charged at physiological pH, and the second ones functionalized with carboxylic groups, negatively charged at physiological pH.

Therefore, we performed a nucleophilic aromatic substitution on the dicarboxylic-acid-derivatized **NDI-18**, using one equivalent of pure **NDI-3**, or **NDI-4** or **NDI-16**. Considering poor solubility and reactivity of **NDI-18**, we forced the S_N_Ar step (step *c*, Figure 4) under harsher conditions: a slight excess of diisopropylethylamine (DIPEA), combined with microwave-assisted protocol in DMF (110 °C, 200 psi, 200 W, in sealed reaction vessels, 20 min), allowed an increase of the reaction yield.

This procedure, however, induced partial reductive dehalogenation of **NDI-18**, allowing the possibility to obtain two different dimeric compounds from each coupling. Dyads were purified in preparative HPLC, obtaining the corresponding trifluoroacetate salts.

The hetero-dyad **NDI-6** was achieved in quantitative yield, treating **NDI-10** in neat *N*^1^,*N*^1^-dimethylpropane-1,3-diamine for 5 minutes, at 180 °C, under microwave assistance.

### 2.2. Preliminary NDI Screening by the G4-CPG Assay

The affinity chromatography-based G4-CPG assay, recently described by some of us [26,27,28,32,33], consists in incubating putative G4 ligands solutions with Controlled Pore Glass (CPG) solid supports functionalized with different secondary structure-forming oligonucleotides. In this screening, defined volumes of a washing solution first, and then of releasing solutions, were flown through the functionalized CPG supports, and all the eluted fractions were separately analyzed by spectrophotometric measurements. The bound ligand was calculated (i) by subtracting the amount of ligand eluted upon treatment with the washing solution (50 mM KCl, 10% DMSO, 10% CH_3_CH_2_OH), derived by direct spectrophotometric measurements, from the ligand amount initially loaded on the supports, or (ii) by direct spectrophotometric measurements of the bound ligand released after treating the supports with the releasing solutions (2.5 M CaCl_2_, 15% DMSO or pure DMSO).

In detail, the here-investigated NDIs (**1**–**12**, Figure 3 and Figure 4) were in parallel tested on three CPG supports, respectively functionalized with the telomeric sequence tel26 [34] and the oncogenic sequence cmyc [35], both able to form unimolecular G4 structures under proper conditions, as well as with the hairpin duplex-forming sequence ds27, used as control.

The binding assays were performed by adopting previously reported protocols [26] (for details, see the Experimental Section). Ligand stock solutions were prepared by dissolving a weighed amount of the solid compound under investigation in H_2_O or pure DMSO, for monomeric and dimeric NDIs, respectively. All the compounds proved to be well soluble in the washing/releasing solutions of our binding assays and at the concentration chosen for the binding experiments. After these solubility checks, we first verified the absence of unspecific binding on the solid support by incubating the tested compounds with the nude CPG. Then, hese ligands were analyzed on the G4- and hairpin-duplex-functionalized CPG supports. The results of the binding assays are reported in Table 1 and Supplementary Figures S1–S3, showing no significant unspecific interaction with the solid support for the tested NDIs, as well as quantitative recovery of the bound ligands by using the releasing solutions.

All the analyzed ligands exhibited a good affinity for tel26 and cmyc G4s immobilized on the solid supports, with the monomeric NDIs showing, in general, a higher affinity than the dimeric compounds (Table 1 and Supplementary Figures S1–S3). Moreover, the tested NDIs proved to be all effective G4 ligands, also in comparison with known G4 binders [26,27]. Among the investigated compounds, **NDI-9** and **NDI-10** showed the lowest affinity for the G4s. On the other hand, the best G4 ligands proved to be **NDI-3**, **NDI-4** and **NDI-5**, for their ability to tightly interact with both telomeric and extra-telomeric G4s (Table 1). In order to evaluate the G4 vs. duplex DNA selectivity, all the compounds were also tested in their interaction with the ds27 hairpin duplex-forming oligonucleotide. Overall, all the NDIs were able to effectively discriminate G4- vs. duplex-forming oligonucleotides (Table 1 and Supplementary Figure S3). In Table 1, the selectivity indexes are reported, calculated as the ratios between the percentages of ligand bound to G4- and hairpin duplex-functionalized supports. In detail, **NDI-3**, **NDI-4**, **NDI-7**, **NDI-9** and **NDI-10** were retained by the telomeric sequence tel26 and cmyc G4s, with a two-fold higher efficacy than by ds27, while **NDI-5** bound tel26 and cmyc G4s more than three times stronger compared to ds27 (Table 1). On the other hand, the investigated ligands showed no ability to discriminate tel26 from cmyc G4s (Table 1). On the basis of their good affinity for the G4-forming sequences and significant G4 vs. duplex DNA selectivity (selectivity index > 2.0), **NDI-3**, **NDI-4**, **NDI-5**, **NDI-7**, **NDI-9** and **NDI-10** were selected as the most promising ligands, according to the G4-CPG assay. Particularly, **NDI-5** emerged as the ligand with the highest G4 vs. duplex DNA selectivity in the investigated series (selectivity index = 3.3 for both **CPG-tel26**/**CPG-ds27** and **CPG-cmyc**/**CPG-ds27**). These compounds were thus advanced to deeper investigation and evaluated in their cytotoxic activity on both cancer and normal cells.

### 2.3. Biological Assays

In order to evaluate the antitumor potential of the most promising monomeric (**NDI-3**, **NDI-4** and **NDI-5**) and dimeric (**NDI-7**, **NDI-9** and **NDI-10**) compounds, a number of in vitro experiments were carried out.

**Figure 5:**
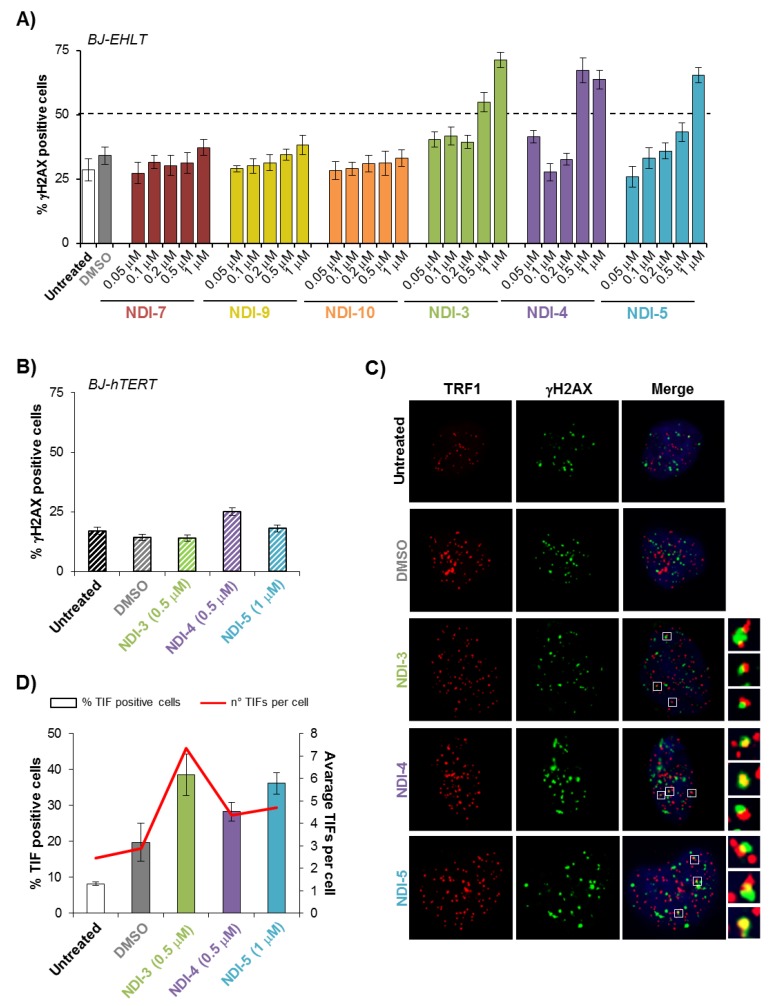
BJ-EHLT and BJ-hTERT fibroblasts were untreated or treated with DMSO or the investigated NDIs at the indicated concentrations for 24 h. Cells were processed for immunofluorescence (IF), using antibodies against γH2AX and TRF1, to visualize the DNA damage and telomeres, respectively. (**A**) Percentages of γH2AX-positive BJ-EHLT cells. (**B**) Percentages of γH2AX-positive BJ-hTERT cells. (**C**) Representative merged images of IF of untreated and treated BJ-EHLT cells; γH2AX spots in green, TRF1 spots in red and nuclei in blue. Enlarged views of Telomere Induced Foci (TIFs) are reported on the right panels of each picture. The images were acquired with a Leica Deconvolution microscope (magnification 63x). (**D**) Quantitative analysis of TIFs. The graph represents the percentages of TIF-positive cells (bars) and the mean number of TIFs per cell (red line) in the indicated samples. Cells with at least four γH2AX/TRF1 foci were scored as TIF positive. Histograms show the mean values ± SD of three independent experiments.

First, human transformed fibroblasts (BJ-EHLT) were treated for 24 h with growing concentrations (ranging from 0.05 to 1 μM) of each NDI, and the DNA damage was evaluated. Analyses on these cells performed by immuno-fluorescence (IF) microscopy showed that, among the tested NDIs, **NDI-3**, **NDI-4** and **NDI-5** produced an increased amount of phosphorylated H2AX (γH2AX), a hallmark of DNA double-strand breaks (Figure 5A). Next, to evaluate the selectivity of the tested molecules toward the transformed cells, DNA damage was also assayed in normal fibroblasts (BJ-hTERT). As reported in Figure 5B, the minimal effective concentration of **NDI-3**, **NDI-4** and **NDI-5** (defined on the basis of their capability to induce damage in at least 50% of treated BJ-EHLT cells) was unable to induce DNA damage in normal cells.

Finally, to evaluate if the DNA damage induced by **NDI-3**, **NDI-4** and **NDI-5** on the transformed cells was located at telomeres, BJ-EHLT cells were treated for 24 h, with each of the three compounds, and the telomeric damage was evaluated by measuring the co-localization spots (telomere-induced foci, TIFs) between γH2AX and TRF1, an effective marker for interphase telomeres (Figure 5C). Interestingly, quantitative analysis showed that the effects of the three compounds were quite similar, and all of them induced a percentage of TIF positive cells (cells with at least four co-localization spots) and average number of TIFs per cell (Figure 5D) comparable with the values recently found for other effective NDI dyads [21]. From these data, it is possible to conclude that the selected NDIs are not only potent and effective inducers of telomere localized DNA damage response, but also show a high grade of specificity against transformed cells.

**Figure 6:**
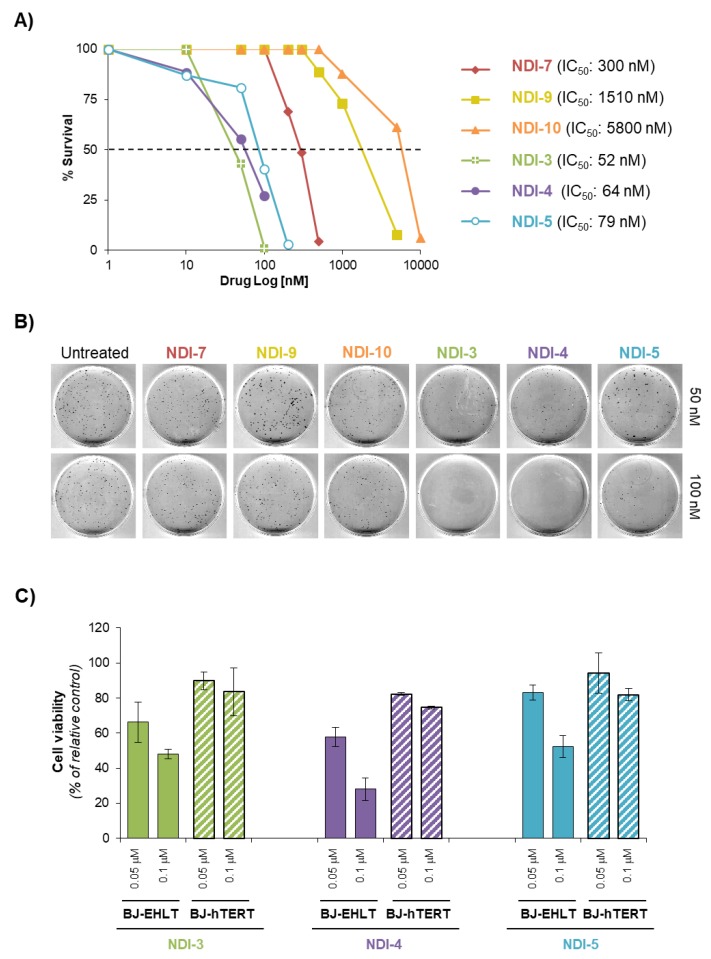
(**A**,**B**) Clonogenic activity of human cervical cancer cells, HeLa, treated with the different NDIs at the indicated doses. (**A**) Surviving fractions were calculated as the ratio of absolute survival of the treated sample/absolute survival of the untreated sample. (**B**) Representative images of the clonogenic assay described in (**A**). (**C**) BJ-EHLT and BJ-hTERT cells were treated with compounds **NDI-3**, **NDI-4** and **NDI-5**, at the indicated doses for 24 h. Viable cell number was determined by colorimetric crystal violet assay. Histograms show the mean values ± SD of three independent experiments.

In addition, the antitumor efficacy of the investigated NDIs was also evaluated in human cervical cancer cells (HeLa) by colony-formation assay (Figure 6A,B). Cells were chronically treated with growing concentrations of **NDI-3**, **NDI-4**, **NDI-5**, **NDI-7**, **NDI-9** or **NDI-10** and the corresponding IC_50_ values were calculated (Figure 6A). Interestingly, **NDI-3**, **NDI-4** and **NDI-5** proved to be the most effective compounds (Figure 6A,B), with IC_50_ values in the low nanomolar range (52, 64 and 79 nM, respectively). In addition, cell survival experiments evidenced a good correlation between antiproliferative activity and ligand ability to induce DNA damage.

To complete the biological screening, the effect of the most active NDIs (**NDI-3**, **NDI-4** and **NDI-5**) on cell viability was evaluated on both normal and transformed cells. Briefly, BJ-hTERT and BJ-EHLT cells were treated for 24 h with two different ligand concentrations (50 and 100 nM) close to their IC_50_, and the number of viable cells was evaluated (Figure 6C). Remarkably, these experiments clearly evidenced that the cytotoxic effects of these ligands on normal cells are significantly lower than those produced on cancer cells, indicating their high selectivity for cancer cells.

### 2.4. Solution Studies on the Interaction of NDI-5 with Oligonucleotide Models by CD and Fluorescence Experiments

**Figure 7:**
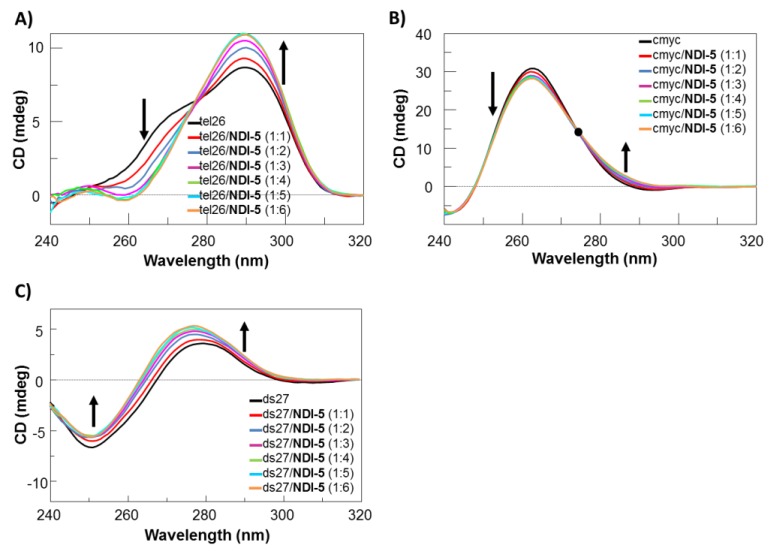
CD spectra of 2 µM solutions of (**A**) tel26, (**B**) cmyc and (**C**) ds27 in 20 mM KCl, 5 mM KH_2_PO_4_ and 10% DMSO buffer (pH 7), in the absence and presence of increasing amount of **NDI-5** (up to six equivalents). Isosbestic point in (B) is shown as black dot; black arrows indicate intensity variations of the specific bands going from 1:1 to 1:6 oligonucleotide/**NDI-5** ratio.

Based on the results of biological and G4-CPG assays, **NDI-5** emerged as the compound that showed very good antitumoral activity, as well as the highest G4 vs. duplex selectivity. Starting from these data, we decided to get a deeper insight into the interaction of **NDI-5** with the human telomeric G4 tel26, the extra-telomeric G4 cmyc and the duplex structure ds27—i.e., the same models used in the G4-CPG assays—by analyzing these systems in solution via CD and fluorescence experiments. In parallel, we also investigated the behavior of one representative dimeric analogue with lower activity, i.e., **NDI-9**. Solutions of all the investigated oligonucleotides in a 20 mM KCl, 5 mM KH_2_PO_4_ and 10% DMSO buffer (pH 7) were titrated with increasing amounts of **NDI-5** or **NDI-9**, and CD spectra were recorded after each addition. As expected in the above buffers, CD spectra showed that tel26 folded into a hybrid G4 structure, featured by a maximum at 290 nm and a shoulder at 270 nm [34], and cmyc formed a parallel G4, with a maximum centered at 263 nm [35,36], while ds27 showed a positive band at 280 nm, with a minimum at 251 nm, characteristic of a B-DNA duplex structure [37] (Figure 7 and Supplementary Figure S4). In the case of tel26 titration with **NDI-5**, a dose-dependent increase of the intensity of the 290 nm band (ΔCD_(DNA/ligand,1:6)-(free DNA)_ = +2.26) and a reduction of the 270 nm shoulder (ΔCD = −2.57) were observed (Figure 7A). In the titration experiments with cmyc, a reduction of the 263 nm band (ΔCD = −2.74), an increase of the 288 nm band (ΔCD = +2.11) and an isosbestic point at 274 nm were observed (Figure 7B). As far as the titration of ds27 is concerned, the addition of **NDI-5** produced a slight reduction of the 251 nm band (ΔCD = −1.07) and an increase of the 280 nm band (ΔCD = +1.75), accompanied by a hypsochromic shift of 3 nm (Figure 7C). In contrast, **NDI-9** caused no evident conformational changes on tel26 G4 and ds27 duplex, and a reduction of the band intensity at 263 nm (ΔCD = −2.03) on cmyc G4 (Supplementary Figure S4). Overall, **NDI-5** proved to affect G4 structures to a greater extent than **NDI-9**, in line with the results evidenced by the G4-CPG and biological assays. Finally, no induced CD signal was observed for all the investigated systems (data not shown).

CD-melting experiments were also performed to evaluate if stabilizing or destabilizing effects on the tested G4s and hairpin duplex structures were obtained upon incubation with **NDI-5** or **NDI-9** (Supplementary Figure S5). CD-melting curves of tel26, cmyc and ds27, in the absence or presence of the ligand (1:6 oligonucleotide/ligand ratio), were recorded by following the CD changes at the wavelength of intensity maximum (290, 263 and 251 nm for tel26, cmyc and ds27, respectively). The results of the CD-melting experiments showed that compound **NDI-5** produced stabilizing effects in all cases, while **NDI-9** was able to stabilize only the cmyc G4, even if to a lower extent than **NDI-5** (Supplementary Figure S5). Only in the case of tel26, tel26/**NDI-5** 1:6 and tel26/**NDI-9** 1:6, the CD melting curves were featured by well-defined sigmoidal profiles and the melting temperatures could be thus determined. **NDI-5** proved to strongly stabilize the hybrid tel26 G4, producing a ΔT_m_ of +15 °C. On the other hand, no difference in terms of melting temperature was found for tel26/**NDI-9** 1:6 system, compared to the free tel26 G4, in full agreement with its lower G4 binding affinity, compared to **NDI-5**, as determined by the G4-CPG assay.

Overall, these results demonstrated the ability of **NDI-5** to affect the structure and stabilize tel26 and cmyc G4s to a greater extent than the model duplex DNA.

Interestingly, **NDI-5** was also able to induce the formation of G4 structures on unstructured tel26 sequences. For these experiments, solutions of tel26 in 10 mM Tris-HCl and 10% DMSO buffer (pH 7) were titrated with increasing amounts of the NDI, and CD spectra were recorded after each addition (Supplementary Figure S6). Remarkably, unfolded tel26, when treated with **NDI-5**, formed a hybrid-type G4 structure [34], featured by T_m_ of 37 °C. On the contrary, no data about the NDI ability to induce structuring of cmyc and ds27 could be gained, since these systems proved to fold into stable secondary structures, i.e., a parallel G4 and a hairpin duplex, respectively, even in the 10 mM Tris-HCl and 10% DMSO buffer (pH 7), lacking of G4- or duplex-stabilizing metal cations (Supplementary Figure S7).

To get information about the binding stoichiometry and constants for the complexes formed between **NDI-5** and the here-studied tel26, cmyc and ds27 secondary structure-forming sequences, fluorescence experiments were carried out.

**Figure 8:**
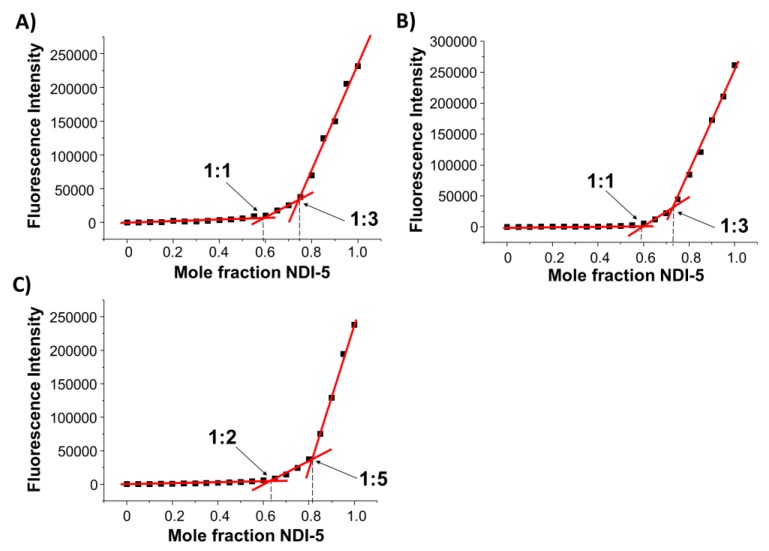
Job plot analyses for the binding of **NDI-5** to (**A**) tel26, (**B**) cmyc and (**C**) ds27. The total molar concentration ([ligand] + [DNA]) was kept constant at 2 µM. The experiments were performed in 20 mM KCl, 5 mM KH_2_PO_4_ and 10% DMSO buffer (pH 7). The excitation wavelength was 526 nm, and the here-reported fluorescence intensity was taken at 589 nm.

Fluorescence spectra were recorded for different DNA/ligand mixtures, prepared by varying the NDI mole fraction from 0 to 1 and keeping the total molar concentration ([ligand] + [DNA]) constant at 2 µM (Figure 8 and Supplementary Figure S8). The analysis of the Job plot for tel26/**NDI-5** mixtures showed changes at NDI mole fractions of 0.59 and 0.75, corresponding to stoichiometry ratios of approximately 1:1 and 1:3 tel26/**NDI-5** (Figure 8A). The stoichiometry of the two binding events suggests the formation of a complex in which, first, one NDI binds the hybrid G4, probably by end-stacking, and then two additional NDI molecules, or one NDI dimer, bind the G4 to the free outer G-quartet by end-stacking or to the G4 grooves/loops [38]. Similar behavior was found for cmyc/**NDI-5** mixtures; indeed, slope changes were found at NDI mole fractions of 0.59 and 0.73, corresponding to stoichiometry ratios of 1:1 and 1:3 cmyc/**NDI-5** (Figure 8B), thus indicating the same peculiar binding mode of **NDI-5** also for parallel G4s. On the other hand, the Job plot analysis for ds27/**NDI-5** mixtures evidenced two different binding events, at NDI mole fractions of 0.63 and 0.82, respectively, corresponding to stoichiometry ratios of approximately 1:2 and 1:5 ds27/**NDI-5** (Figure 8C). In the latter case, the very high stoichiometry of binding suggests that unspecific interactions might also occur between **NDI-5** and the DNA duplex target.

**Figure 9:**
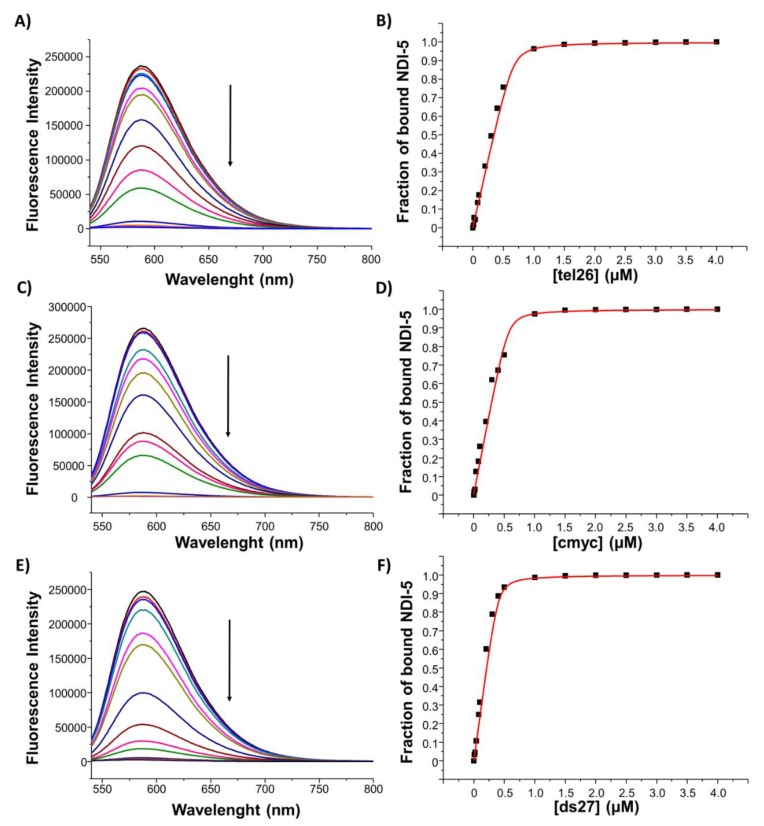
Fluorescence titration experiments for **NDI-5**. Left: fluorescence spectra obtained by adding increasing amounts of (**A**) tel26, (**C**) cmyc and (**E**) ds27 to 2 µM solutions of **NDI-5**. Right: representative binding curves obtained by plotting the fraction of bound ligand **NDI-5** as a function of (**B**) tel26 G4, (**D**) cmyc G4 and (**F**) ds27 hairpin duplex concentration. The black squares represent the experimental data; the red line is the best fit obtained with an independent and equivalent-sites model. The experiments were performed in 20 mM KCl, 5 mM KH_2_PO_4_ and 10% DMSO buffer (pH 7). The excitation wavelength was 526 nm. The arrows indicate increasing DNA concentration.

In addition, fluorescence titration experiments were carried out at a fixed concentration of ligand, by adding increasing amounts of all the investigated oligonucleotides. In detail, 2 μM **NDI-5** solutions were, in parallel, titrated with increasing amounts of tel26, cmyc and ds27 solutions, and the corresponding fluorescence spectra, after each addition, were recorded (Figure 9, left). In all cases, a significant fluorescence quenching was observed, further demonstrating the interaction between the NDI and the oligonucleotides. Then, the fraction of bound NDI was calculated from the obtained fluorescence intensity values and reported as a function of the DNA concentration (Figure 9, right). Finally, data fittings with an independent and equivalent-sites model [32,39] provided stoichiometries of 1:3, 1:3 and 1:5 for tel26/**NDI-5**, cmyc/**NDI-5** and ds27/**NDI-5**, respectively, in perfect agreement with the previously described Job plot analyses. In addition, binding constants of 2.4 (±0.1) × 10^7^, 2.6 (±0.2) × 10^7^ and 1.9 (±0.2) × 10^7^ M^−1^ were determined for tel26/**NDI-5**, cmyc/**NDI-5** and ds27/**NDI-5**, respectively. These results showed that **NDI-5** displayed high affinity for cmyc and tel26 G4s, also indicating a better recognition of G-quadruplex over duplex structures, in agreement with G4-CPG assay and CD data. Conversely, no evidence of tight binding was found for **NDI-9** toward all the investigated oligonucleotides, by either Job plot or titration analyses (Supplementary Figure S9), in line with the results of the G4-CPG assay, the CD experiments and the biological assays.

## 3. Conclusions

A focused library of novel functionalized monomeric and dimeric NDIs was designed, synthesized and evaluated by the G4-CPG assay. All tested compounds proved to be effective G4 ligands. In particular, monomeric **NDI-3**, **NDI-4** and **NDI-5** and dimeric **NDI-7**, **NDI-9** and **NDI-10** emerged as the most promising compounds, due to their good G4 vs. duplex DNA selectivity. The biological assays evidenced **NDI-3**, **NDI-4** and **NDI-5** as the compounds with the highest antitumoral activity, due to their ability to selectively target cancer vs. normal cells by inducing telomeric DNA damage and cytotoxic effects.

Considering that G4-CPG assays’ data indicated **NDI-5** as the best ligand in terms of G4 vs. duplex DNA discrimination ability, this was selected for further investigations by CD and fluorescence spectroscopy analyses. CD titrations and CD-melting experiments clearly demonstrated the capacity of **NDI-5** to preferentially affect G4 structures, stabilizing them, rather than duplex DNA, as well as to induce G4 formation even in the absence of metal cations. Furthermore, fluorescence experiments proved that **NDI-5** was able to form peculiar 1:3 complexes with both hybrid and parallel G4 structures; in contrast, the binding of **NDI-5** to duplex structures seemed to be essentially driven by unspecific interactions.

Our efforts to produce novel hetero-dimers, following the idea to reduce the electrostatic interactions in order to limit the unspecific ligand-DNA binding modes, proved to be only partially successful. In fact, the dimeric NDIs showed good binding properties and selectivity toward the G4s, as indicated by the G4-CPG data, but were less cytotoxic than the related monomers.

Summing up, trifunctionalized monomeric NDIs emerged as the most efficient and selective G4 ligands, as well as potent cytotoxic compounds. In particular, **NDI-5**, decorated with two *N*,*N*-dimethylaminopropyl moieties and one oligoethyleneglycol chain, proved to be a very promising anticancer agent. Its remarkable and selective bioactivity, associated with high water solubility, indicated it as a valid lead compound for the development of effective G4-targeting hits, which can be advanced to more in-depth biological and structural studies.

## 4. Experimental Section

### 4.1. Chemistry

All solvents and reagents were purchased from TCI and Sigma-Aldrich and used without further purification.

HPLC analyses were performed by using an Agilent system SERIES 1260 with XBridge^®^ BEH C18 column (2.5 µm, 4.6 × 50 mm). The following method was used: flow 1.4 mL/min, isocratic gradient over 2 min 95% of H_2_O + 0.1% TFA (5% CH_3_CN), gradually to 40% aqueous solvent over 6 min, then isocratic flow for 4 min (λ = 256 nm).

HPLC purifications were carried by out using an Agilent Technologies 1260 Infinity preparative HPLC equipped with a diode array detector. A Waters XSelect^®^ CSH C18 column (5 μm, 30 x 100 mm) was employed with different gradient methods (solvent: H_2_O + 0.1% TFA and CH_3_CN, flow 30 mL/min). Method A (for NDIs **1-5**, **15-17**): isocratic flow over 4 min 95% of aqueous solution, gradually to 80% over 8 min, to 70% over the following 4 min and then isocratic gradient over 4 min (λ = 254, 500 nm). Method B (for NDIs **7-12**): isocratic flow over 2 min 85% of aqueous solution, gradually to 60% over 8 min, to 40% over the following 10 min and then to 0% over 5 min (λ = 254, 500 nm). Method C (for **NDI-6**): isocratic flow over 2 min 95% of aqueous solution, gradually to 0% over 18 min (λ = 254, 600 nm).

UPLC-MS data were recorded, using a surveyor UPLC system (Thermo Finnigan, San Jose, CA, USA) equipped with a BEH Acquity UPLC column (1.7 µm) 2.1 × 50 mm, and an LCQ ADV MAX ion-trap mass spectrometer, with an ESI ion source.

^1^H-NMR and ^13^C-NMR spectra were recorded on Bruker Avance 300 and 400 MHz and calibrated to the internal standard TMS or the residual solvent peak.

All new compounds characterization spectra are reported in Pages S10–S34.

### 4.2. Synthesis of NDIs 1–5, 15–17 (Figure 3)

The 2,6-dibromo-1,4,5,8-naphthalenetetracarboxylic acid (500 mg, 1.17 mmol), synthesized as previously reported, [15] was suspended in acetic acid at 90 °C. After complete dissolution, 2.5 equivalents of *N*^1^,*N*^1^-dimethylpropane-1,3-diamine (300 mg, 2.93 mmol) were added, and the reaction was refluxed for 30 min at 120 °C. The reaction mixture was then poured into an ice-cooled saturated Na_2_CO_3_ aq. solution, until pH 8, and subsequently extracted with chloroform. The organic layer was dried over anhydrous MgSO_4_, and the solvent was removed under vacuum. The product was obtained as a mixture of 2-bromo (**NDI**-**13**, 35%) and 2,6-dibromo (**NDI**-**14**, 65%) NDIs (Figure 3), and it was used for the successive step, without purification. To obtain **NDI-1** and **NDI-15**, 0.5 mmol of the **NDI**-**13** and **NDI**-**14** mixture product was dissolved in 40 mL of CH_3_CN, and 2.5 equivalents of n-propylamine were added. The mixture was stirred at 75 °C for 2 h, under argon atmosphere. The resulting red solution was concentrated under vacuum and purified by preparative HPLC, according to method A (reported in Section 4.1), obtaining products as trifluoroacetate salts.

The same procedure was used to synthetize **NDI-3** and **NDI-17**, **NDI-4** and **NDI-16**, and **NDI-5**, just replacing n-propylamine with 1,7-diaminoheptane, 2,2-(ethylenedioxy)bis(ethylamine) and 2-(2-aminoethoxy)ethanol respectively (Figure 3).

All the final products were characterized by ESI-MS and ^1^H- and ^13^C-NMR. The characterization data for **NDI-1** [29], **NDI-3** [21], **NDI-15** [29] and **NDI-17** [21] were previously reported.

**NDI-4** (UPLC-MS, positive mode): 189.1 (**NDI-4**+3H)^3+^, 283.2 (**NDI-4**+2H)^2+^, 565.4 (**NDI-4**-H)^+^ *m*/*z*.

**NDI-4•3CF_3_COOH** (yield 40%). ^1^H-NMR (300 MHz, CD_3_OD): 8.28 (d, *J* = 7.7, 1H), 8.01 (d, *J* = 7.8, 1H), 7.97 (s, 1H), 4.28-4.18 (m, 4H), 3.94 (m, 2H), 3.83-3.81 (m, 8H), 3.34-3.29 (m, 4H), 3.21 (m, 2H), 2.97 (s, 6H), 2.95 (s, 6H), 2.25-2.20 (m, 4H). ^13^C-NMR (75 MHz, CD_3_OD): 167.4, 164.0, 163.9, 163.3, 153.6, 138.9, 130.3, 129.1, 125.1, 124.9, 123.3, 122.3, 121.0, 101.2, 71.9, 71.7, 70.9, 68.3, 57.1, 44.2, 43.9, 41.0, 39.2, 38.4, 24.9, 24.8.

**NDI-5** (UPLC-MS, positive mode): 270.7 (**NDI-5**+2H)^2+^, 540.4 (**NDI-5**+H)^+^ m/z.

**NDI-5•2CF_3_COOH** (yield 60%) ^1^H-NMR (300 MHz, D_2_O): 8.13 (d, *J* = 7.9, 1H), 7.90 (d, *J* = 7.9, 1H), 7.77 (s, 1H), 4.08-4.01 (m, 4H), 3.84 (t, *J* = 4.8, 2H), 3.67-3.66 (m, 6H), 3.22-3.12 (m, 4H), 2.82 (s, 12H), 2.11-1.99 (m, 4H). ^13^C-NMR (75 MHz, D_2_O): 165.3, 163.8, 163.5, 163.2, 163.0, 151.9, 130.7, 128.3, 126.6, 125.0, 124.0, 121.9, 120.1, 118.3, 98.4,71.8, 68.6, 60.4, 55.1, 42.6, 42.2, 37.6, 37.0, 32.6, 22.6.

**NDI-16•3CF_3_COOH** (yield 60%). ^1^H-NMR (300 MHz, CD_3_OD): 8.80 (s, 1H), 8.46 (s, 1H), 4.39-4.35 (m, 4H), 4.01-3.97 (m, 2H), 3.90-3.86 (m, 8H), 3.43-3.40 (m, 4H), 3.28-3.25 (m, 2H), 3.04-3.03 (m, 12H), 2.32-2.18 (m, 4H). ^13^C-NMR (75 MHz, CD_3_OD): 167.1, 164.8, 164.6, 164.5, 153.7, 132.2, 130.4, 128.9, 127.3, 125.4, 124.2, 121.3, 120.4, 100.4, 71.9, 71.7, 70.8, 68.3, 57.0, 44.2, 43.8, 41.0, 39.0, 38.5, 24.9.

Starting from pure **NDI-15**, **NDI-2** was obtained with a yield of 68%, exploiting a second harsher microwave-assisted S_N_Ar. Then, 0.32 mmol of **NDI-15** were dissolved in neat *N*^1^,*N*^1^-dimethylpropane-1,3-diamine (1 mL), in a sealed vessel. The microwave-assisted reaction was performed at 150 °C for 5 minutes, under stirring. After cooling down the reaction mixture to room temperature, an acidic aqueous solution was added, and the resulting purple mixture was purified by preparative HPLC, using method A (reported in Section 4.1). The characterization of **NDI-2** was previously reported [29].

### 4.3. Synthesis of NDI 18 and Hetero-Dyads 6–12 (Figure 4)

The 2,6-dibromo-1,4,5,8-naphthalenetetracarboxylic acid (500 mg, 1.17 mmol) and 6-aminohexanoic acid (630 mg, 4.78 mmol) were suspended in glacial acetic acid in a sealed vessel. After sonication, the microwave-assisted reaction was performed under stirring at 120 °C, 250 psi, for 20 min. After cooling down the system to room temperature, the solution was diluted with water, and few drops of concentrated HCl were added, to obtain **NDI-18** as a precipitate (yield 52%). The suspension was then filtered, obtaining **NDI-18** as a pure solid, which was characterized by ESI-MS and ^1^H- and ^13^C-NMR.

**NDI-18** (UPLC-MS, negative mode): 651.1 (**NDI-18** -H)^-^ *m*/*z*.

**NDI-18**^1^H-NMR (300 MHz, DMSO-d^6^): 12.01 (s, 2H), 8.63 (s, 2H), 4.03-4.01 (m, 4H), 2.25-2.21 (m, 4H), 1.65-1.56 (m, 8H), 1.39-1.37 (m, 4H). ^13^C-NMR (75 MHz, DMSO-d^6^): 174.4, 162.5, 162.3, 161.6, 161.5, 160.6, 160.5, 137.0, 136.4, 131.1, 130.4, 130.2, 127.4, 126.4, 126.2, 126.0, 125.7, 125.5, 125.2, 33.4, 27.1, 27.0, 27.0, 26.9, 26.0, 24.2.

In order to obtain the hetero dyads **7-12**, 0.95 equivalent of **NDI-18** (111 mg, 0.17 mmol), 5 equivalents of DIPEA (155 µL, 0.9 mmol) and 1 equivalent of opportune positive charged monomeric NDI unit were dissolved in 5 mL of DMF. In particular, monomeric **NDI-4** (105 mg, 0.18 mmol) was used to obtain dimeric **NDI-7** and **NDI-9**; **NDI-16** (119 mg, 0.18 mmol) to **NDI-8** and **NDI-10**; **NDI-3** (102 mg, 0.18 mmol) to **NDI-11**; and **NDI-17** (119 mg, 0.18 mmol) to **NDI-12**.

The microwave-assisted reaction was performed under stirring, at 110 °C, 200 psi, for 20 min, in a sealed vessel. After cooling down the system to room temperature, acidic aq. solution and DMSO were added to dissolve the precipitate. The solution was then purified by preparative HPLC, using method B (reported in Section 4.1). The products were obtained as trifluoroacetate salts.

**NDI-7** (UPLC-MS, positive mode): 538.3 (**NDI-7**+2H)^2+^, 1075.6 (**NDI-7**+H)^+^ *m*/*z*.

**NDI-7•2CF_3_COOH** (yield 13%). ^1^H-NMR (300 MHz, CD_3_OD): 7.87 (d, *J* = 8, 1H), 7.82 (d, *J* = 8, 1H), 7.79 (s, 1H), 7.73 (d, *J* = 8, 1H), 7.68 (s, 1H), 7.67 (d, *J* = 8, 1H), 4.18 (m, 4H), 4.07-4.03 (m, 8H), 3.94-3.90 (m, 4H), 3.63-3.62 (m, 4H), 3.43-3.40 (m, 2H), 3.29-3.23 (m, 2H), 3.05 (s, 6H), 2.96 (s, 6H), 2.28 (m, 2H), 2.11 (m, 1H), 1.82-1.30 (m, 16H). ^13^C-NMR (75 MHz, CD_3_OD): 177.8, 177.7, 166.5, 166.3, 164.5, 164.1, 163.9, 163.9, 163.7, 153.1, 152.9, 131.8, 131.6, 130.0, 129.7, 128.6, 128.5, 127.0, 125.3, 125.1, 123.4, 123.2, 120.8, 120.4, 119.8, 119.6, 100.0, 99.9, 72.3, 72.2, 70.2, 57.2, 57.0, 44.1, 44.0, 43.9, 42.1, 41.4, 39.1, 38.4, 35.0, 34.9, 28.9, 28.7, 27.9, 27.8, 26.1, 25.9, 25.0.

**NDI-8** (UPLC-MS, positive mode): 617.3 (**NDI-8**+2H)^2+^, 1233.2 (**NDI-8**+H)^+^ *m*/*z*.

**NDI-8•2CF_3_COOH** (yield 10%). ^1^H-NMR (300 MHz, D_2_O): 7.65 (s, 2H), 7.46 (fd, 2H), 7.32 (s, 1H), 3.97 (m, 4H), 3.85 (m, 8H), 3.67-3.65 (m, 4H), 3.42 (m, 4H), 3.27-3.22 (m, 2H), 3.17-3.03 (m, 2H), 2.90 (s, 6H), 2.81 (s, 6H), 2.06 (m, 2H), 1.89 (m, 2H), 1.70-1.45 (m, 16H). ^13^C-NMR (75 MHz, D_2_O): 178.5, 178.4, 164.3, 163.7, 162.3, 160.4, 160.4, 159.9, 159.9, 151.1, 150.1,136.2, 130.6, 130.4, 127.5, 126.1, 125.9, 124.7, 123.6, 121.0, 120.7, 120.4, 119.5, 97.7, 97.4, 69.9, 68.8, 67.9, 55.1, 54.9, 42.7, 42.6, 37.6, 35.5, 33.4, 28.0, 26.9, 26.7, 25.7, 25.6, 23.8, 23.8, 22.7.

**NDI-9** (UPLC-MS, positive mode): 578.3 (**NDI-9**+2H)^2+^, 1155.4 (**NDI-9**+H)^+^ *m*/*z*.

**NDI-9•2CF_3_COOH** (yield 12%). ^1^H-NMR (300 MHz, D_2_O): 7.50 (s, 1H), 7.44-7.33 (m, 3H), 7.03 (s, 1H), 3.89-3.80 (m, 12H), 3.69-3.64 (m, 4H), 3.48-3.41 (m, 2H), 3.24-3.10 (m, 6H), 2.88 (s, 6H), 2.83 (s, 6H), 2.42-2.38 (m, 4H), 1.63-1.25 (m, 16H). ^13^C-NMR (75 MHz, D_2_O): 178.4, 178.3, 163.7, 163.0, 161.9, 161.4, 160.9, 160.8, 160.5, 150.3, 150.2, 130.4, 126.6, 125.4, 125.0, 123.8, 123.4, 122.2, 121.2, 120.6, 120.4, 120.2, 119.3, 119.2, 118.5, 118.1, 116.4, 97.7, 96.9, 70.1, 69.4, 67.8, 66.2, 54.9, 42.6, 38.9, 38.6, 33.4, 33.3, 26.8, 26.5, 25.7, 25.5, 23.8, 23.7, 22.6, 22.4.

**NDI-10** (UPLC-MS, positive mode): 578.3 (**NDI-9**+2H)^2+^, 1155.5 (**NDI-9**+H)^+^ *m*/*z*.

**NDI-10•2CF_3_COOH** (yield 10%). ^1^H-NMR (300 MHz, D_2_O): 7.50 (s, 1H), 7.44-7.33 (m, 3H), 7.03 (s, 1H), 3.89-3.80 (m, 12H), 3.69-3.64 (m, 4H), 3.41 (m, 2H), 3.24-3.10 (m, 6H), 2.88 (s, 6H), 2.83 (s, 6H), 1.96-1.92 (m, 4H), 1.63-1.30 (m, 16H). ^13^C-NMR (75 MHz, D_2_O): 178.4, 178.3, 163.7, 163.0, 161.9, 161.4, 160.9, 160.8, 160.5, 150.4, 150.2, 126.6, 125.4, 125.0, 123.8, 123.4, 121.2, 120.6, 120.4, 120.2, 119.3, 119.2, 118.5, 118.1, 116.4, 97.7, 96.9, 70.1, 69.4, 67.8, 66.2, 54.9, 42.6, 38.8, 38.6, 33.4, 33.3, 26.7, 26.5, 25.7, 25.5, 23.8, 23.7, 22.6, 22.4.

**NDI-11** (UPLC-MS, positive mode): 529.4 (**NDI-11**+2H)^2+^, 1057.6 (**NDI-11**+H)^+^ *m*/*z*.

**NDI-11•2CF_3_COOH** (yield 13%). ^1^H-NMR (400 MHz, DMSO-d^6^): 8.28 (d, *J* = 7.7, 1H), 8.16 (d, *J* = 6.8, 1H), 8.00 (d, *J* = 7.7, 1H), 7.89 (m, 2H), 7.83 (bs, 1H), 4.05-4.01 (m, 4H), 4.0-3.8 (m, 4H), 3.57-3.56 (m, 8H), 3.17 (bs, 4H), 2.78 (s, 12H), 2.23 (t, *J* = 7.3, 4H), 2.02 (bs, 4H), 1.81 (bs, 4H), 1.59-1.54 (m, 12H), 1.35-1.34 (m 4H). ^13^C-NMR (100 MHz, DMSO-d^6^): 174.6, 165.5, 165.3, 162.9, 162.6, 162.4, 162.3, 162.1, 161.9, 158.4, 158.0, 151.7, 151.6, 128.7, 128.5, 127.6, 127.3, 125.7, 125.5, 122.5, 122.3, 119.2, 118.7, 118.5, 98.6, 98.4,54.7, 54.6, 42.4, 42.3, 37.5, 34.5, 33.7, 33.6, 28.3, 27.2, 26.3, 26.2, 26.1, 24.4, 24.4, 23.1, 23.0, 13.7.

**NDI-12** (UPLC-MS, positive mode): 569.3 (**NDI-12**+2H)^2+^, 1137.6 (**NDI-12**+H)^+^ *m*/*z*.

**NDI-12•2CF_3_COOH** (yield 15%). ^1^H-NMR (400 MHz, DMSO-d^6^): 8.23 (s, 1H), 8.20 (d, *J* = 7.8, 1H), 7.96 (m, 2H), 7.83 (s, 1H), 4.11 (bs, 10H), 4.03-3.91 (m, 8H), 3.17 (s, 12H), 2.23 (t, *J* = 7.3, 4H), 2.01 (m, 4H), 1.81 (bs, 4H), 1.59-1.53 (m, 14H), 1.37-1.33 (m, 4H). ^13^C-NMR (100 MHz, DMSO-d^6^): 174.6, 165.2, 162.5, 162.1, 161.9, 161.4, 161.2, 160.8, 151.6, 151.2, 149.4, 137.0, 136.4, 130.5, 128.4, 127.9, 127.3, 127.1, 125.5, 123.6, 122.9, 122.5, 122.3, 120.8, 118.7, 118.4, 98.7, 98.4, 54.7, 48.8, 42.4, 38.0, 33.7, 33.6, 28.1, 27.5, 27.3, 26.3, 26.1, 26.0, 24.4, 23.0, 22.9.

To obtain **NDI-6**, **NDI-10** (10 mg, 0.009 mmol) was dissolved in 2 mL of *N*,*N*-dimethylpropane-1,3-diamine, in a sealed vessel, and the reaction was stirred at 180 °C for 5 min, with the assistance of the microwaves (yield 37%). The solution was then diluted with acidic aqueous solution and purified by preparative HPLC, according to method C (reported in Section 4.1), yielding **NDI-6** as trifluoroacetate salt.

**NDI-6** (UPLC-MS, positive mode): 386.7 (**NDI-6**+3H)^3+^, 579.5 (**NDI-6**+2H)^2+^, 1157.7 (**NDI-6**+H)^+^ *m*/*z*.

**NDI-6•3CF_3_COOH**. ^1^H-NMR (300 MHz, D_2_O): 7.26 (m, 2H), 6.94-6.87 (m, 3H), 3.75-3.66 (m, 8H), 3.43-2.98 (m, 18H), 2.90-2.84 (m, 18H), 2.02-1.34 (m, 22H). ^13^C-NMR (75 MHz, D_2_O): 178.2, 164.0, 163.5, 163.0, 162.6, 162.2, 161.6, 161.4, 161.2, 150.0, 147.5, 147.2, 129.5, 126.5, 125.0, 123.4, 123.4, 123.0, 122.8, 122.1, 120.4, 118.6, 118.2, 116.6, 116.2, 114.3, 100.4, 99.2, 97.0, 69.9, 67.7, 55.1, 55.0, 54.9, 44.4, 43.2, 42.6, 39.5, 37.1, 33.4, 33.3, 26.8, 26.5, 25.6, 25.5, 23.9, 23.8, 23.7, 22.6, 22.1.

### 4.4. G4-CPG Assay

Long-chain AlkylAmine-CPG 1000 Å was functionalized with 3’-*O*-acetyl-5’-*O*-(4,4’-dimethoxytrityl)thymidine through a hexaethylene glycol spacer, as previously described [26,27,28]. Oligonucleotide-functionalized CPG supports were obtained by solid-phase synthesis, using standard phosphoramidite chemistry on an automated Applied Biosystem 394 DNA/RNA synthesizer. By using a 1 µmol-scale and a “DMT-ON” protocol, the following oligonucleotides were elongated on the CPG supports: d[(TTAGGG)_4_TT] (tel26), d(TGGGGAGGGTGGGGAGGGTGGGGAAGGTGGGGA) (cmyc) and d(CGCGAATTCGCGTTTCGCGAATTCGCG) (ds27). The efficiency of each coupling cycle was monitored by spectrophotometric measurements of the DMT cation, released from the support by acidic treatment with 3% TCA in CH_2_Cl_2_ before the subsequent coupling step. Considering the number of couplings and the average yield per cycle of 99.8%, 99.7% and 99.2%, respectively, for tel26, cmyc and ds27, the overall yield was determined to be 95%, 91% and 80%. Stock solutions of each tested NDI were prepared by dissolving a weighed amount of the solid compound in H_2_O or pure DMSO. Before performing binding assays with the here-investigated NDIs, all the oligonucleotide-functionalized supports were tested in their ability to bind a set of known G4 ligands with different affinity for G4s [40,41,42,43,44,45,46]. G4-CPG binding assays were carried out as previously described [26,27,28]. The UV measurements were performed on a JASCO V-550 UV-vis spectrophotometer equipped with a Peltier Thermostat JASCO ETC-505T. The UV quantification of the ligands was determined by measuring the absorbance relative to the λ_max_ characteristic of each ligand and referring it to the corresponding calibration curves. A quartz cuvette with a path length of 1 cm was used.

### 4.5. Biological Experiments

*Cells and culture condition*. Human fibroblasts (BJ) and human cervical cancer cells (HeLa) were used in this study. BJ-hTERT cells were obtained infecting primary BJ cells with a retrovirus carrying hTERT (Addgene plasmid #1773); BJ-EHLT derived from the transformation of BJ fibroblasts with hTERT and SV40 [47] early region (BJ-EHLT). BJ-hTERT, BJ-EHLT and HeLa were grown in Dulbecco Modified Eagle Medium (DMEM, Invitrogen Carlsbad, CA, USA) supplemented with 10% Fetal Bovine Serum (FBS), 2 mM l-glutamine and antibiotics, at 37 °C, in a 5% CO_2_-95% air atmosphere.

*Immunofluorescence.* Cells were fixed in 2% formaldehyde in phosphate buffered saline (PBS) for 10 min at room temperature (rt) and permeabilized in 0.25% Triton X-100 in PBS for 5 min at RT. For immune-labeling, cells were incubated with primary antibody for 2 h, at rt, washed twice in PBS and finally incubated with the secondary antibodies for 1 h. The following primary antibodies were used: Mouse mAb anti-γH2AX (Millipore, Billerica, MA, USA) and Rabbit pAb anti-TRF1 N19 (Santa Cruz Biotechnologies, Santa Cruz, CA, USA). The following secondary antibodies were used: Anti-Mouse IgG (H+L), F(ab’)_2_ Fragment (Alexa Fluor 488 Conjugate) (Cell Signaling) and Anti-rabbit IgG (H+L), F(ab’)_2_ Fragment (Alexa Fluor 555 Conjugate) (Cell Signaling). Nuclei were stained with 4′,6-diamidino-2-phenylindole (DAPI, Sigma). Fluorescence signals were recorded by using a Leica DMIRE2 microscope equipped with a Leica DFC 350FX camera and elaborated by Leica FW4000 deconvolution software (Leica, Solms, Germany). For quantitative analysis of γH2AX positivity, 300 cells on triplicate slices were scored, and for TIF analysis, 30 γH2AX-positive cells were scored. Cells with at least four co-localizations (γH2AX/TRF1) were considered as TIF-positive.

*Clonogenic assay.* Human cervical cancer cells, HeLa, were seeded in 60 mm Petri dishes, at the clonogenic density of 500 cells/plate, in DMEM medium with 10% FBS. After 24 h, cells were treated with compounds **NDI-3, NDI-4, NDI-5, NDI-7, NDI-9** and **NDI-10**. After 10 days, the cells were stained with 2% methylene blue in 50% ethanol, and the number of colonies was counted. Surviving fractions were calculated as the ratio of absolute survival of the treated sample/absolute survival of the untreated sample.

*Viability assay (crystal violet).* BJ EHLT and BJ hTERT fibroblasts were seeded in a 24-well plate, at a density of 1 × 10^4^ and 3 × 10^4^ for well, respectively. Then, the cells were treated with compounds **NDI-3, NDI-4, NDI-5, NDI-7, NDI-9** and **NDI-10** for 24 h and successively washed twice in PBS and fixed with 4% formaldehyde for 15 min at room temperature. After washing, 300 μL of crystal violet staining solution (Sigma) was added to each well and incubated for 30 min at room temperature. Finally, the plates were rinsed twice with water and air-dried, and the cell pellets were dissolved in 400 μL of acetic acid. The optical density of each well in triplicate was measured at 570 nm (OD_570_), with a 96-well plate in an ELISA reader (Thermo Scientific). The average absorbance in each condition was used to calculate the survival expressed as percent of treated vs. untreated condition. IC_50_ (the dose necessary to reduce the survival of 50%) was calculated by Calcusyn software.

### 4.6. CD Experiments

CD spectra were recorded in a quartz cuvette with a path length of 1 cm, on a Jasco J-715 spectropolarimeter equipped with a Peltier-type temperature control system (model PTC-348WI). The spectra were recorded at 20 °C in the range 240–800 nm, with 2 s response, 200 nm/min scanning speed and 2.0 nm bandwidth, and they were corrected by subtraction of the background scan with buffer. All the spectra were averaged over 3 scans. The oligonucleotides d[(TTAGGG)_4_TT] (tel26), d(TGGGGAGGGTGGGGAGGGTGGGGAAGGTGGGGA) (cmyc) and d(CGCGAATTCGCGTTTCGCGAATTCGCG) (ds27) were synthesized by standard automated solid phase oligonucleotide synthesis on an Applied Biosystem 394 DNA/RNA synthesizer. After ammonia treatment (55 °C, 16 h), allowing both deprotection and detachment from the solid support, the crude oligonucleotides were purified by HPLC on a SAX analytical column and then dialyzed against water, using a Float-A-Lyzer G2 dialysis device (MWCO 0.5-1.0 kDa, three H_2_O changes over 24 h). After lyophilization, the oligonucleotides were dissolved in a 20 mM KCl, 5 mM KH_2_PO_4_, 10% DMSO buffer (pH 7), to obtain 2 μM solutions, and then annealed by heating at 95 °C for 5 min, followed by slow cooling to room temperature. The ligand stock solution was 4 mM in H_2_O or DMSO, respectively, for **NDI-5** and **NDI-9**. CD titrations were obtained by adding increasing amounts of the ligands (up to 6 molar equivalents, corresponding to a 12 μM solution in ligand) to tel26, cmyc and ds27. After each ligand addition, the system was allowed equilibrating before recording the spectra. For the CD-melting experiments, the ellipticity was recorded at 290, 263 and 251 nm for tel26, cmyc and ds27, respectively, with a temperature scan rate of 0.5 °C/min, in the range 20–90 °C. For the CD-monitored experiments evaluating the ability of **NDI-5** to induce G-quadruplex structuring, 20 µM solutions of tel26 were prepared in 10 mM Tris-HCl, 10% DMSO buffer (pH 7), and titrated with increasing amounts of NDI (up to 6 molar equivalents, corresponding to a 120 μM solution in ligand). Then the melting curves of these solutions were recorded at 290 nm for tel26/**NDI-5** 1:6 ratio mixtures, with a temperature scan rate of 0.5 °C/min, in the range 10–90 °C.

### 4.7. Fluorescence Experiments

Fluorescence spectra were recorded at 20 °C on HORIBA Jobin Yvon Inc. FluoroMax^®^-4 spectrofluorometer equipped with F-3004 Sample Heater/Cooler Peltier Thermocouple Drive, by using a quartz cuvette with a 1 cm path length. **NDI-5** was excited at 526 nm, and emission spectra were recorded between 540 and 800 nm, while **NDI-9** was excited at 518 nm, and emission spectra were recorded between 530 and 800 nm. Both excitation and emission slits were set at 5 nm. The experiments were performed in 20 mM KCl, 5 mM KH_2_PO_4_, 10% DMSO buffer (pH 7). For the construction of the Job plot, the mole fraction of each ligand was varied from 0 to 1, and the total molar concentration ([ligand] + [DNA]) was kept constant at 2 µM. For fluorescence titration experiments, 2 μM ligands solutions were prepared. Increasing amounts of tel26, cmyc and ds27 (up to 10 μM concentration) were added from 120 μM stock solutions of each DNA sample annealed in 20 mM KCl, 5 mM KH_2_PO_4_, 10% DMSO buffer (pH 7). After each addition, the system was allowed equilibrating 10–15 min before recording the spectra. The fraction of bound **NDI-5** was calculated from the fluorescence intensity at 589 nm and reported in graph as a function of the DNA concentration. The obtained points were fitted with an independent and equivalent-sites model [39], using the Origin 8.0 program, with the following equation:

(1) $$\alpha =\left(\frac{1}{2{\left[L\right]}_{0}}\right)\left\{\left({\left[L\right]}_{0}+n\left[Qu\right]+\frac{1}{K_{b}}\right)-\sqrt{{\left({\left[L\right]}_{0}+n\left[Qu\right]+\frac{1}{K_{b}}\right)}^{2}-4{\left[L\right]}_{0}n\left[Qu\right]}\right\}$$

where *α* is the mole fraction of ligand in the bound form, [*L*]_0_ is the total ligand concentration, [*Qu*] is the added DNA concentration, *n* is the number of the equivalent and independent sites on the DNA structure and *K_b_* is the binding constant. The fraction of the bound ligand was determined by using the following equation:

(2) $$\alpha =\frac{Y-Y_{0}}{Y_{b}-Y_{0}}$$

where *Y*, *Y*_0_ and *Y_b_* are the values of fluorescence emission intensity at the maximum, respectively, at each titrant concentration, at the initial state and the final state of the titration.

## Figures and Tables

**Table 1:** Summary of the binding assays’ data for monomeric and dimeric NDIs on nude and functionalized CPG supports, and selectivity indexes calculated as the ratio between the percentages of ligand bound to the indicated G4- and hairpin duplex-functionalized supports.

Compound	Bound Ligand (%)^a^				Selectivity Index		
	Nude CPG	CPG-tel26	CPG-cmyc	CPG-ds27	CPG-tel26/ CPG-ds27	CPG-cmyc/ CPG-ds27	CPG-tel26/ CPG-cmyc
**NDI-1**	4	85	95	50	1.7	1.9	0.9
**NDI-2**	0	92	94	46	2.0	2.0	0.9
**NDI-3**	0	100	97	48	2.1	2.0	1.0
**NDI-4**	7	97	95	41	2.4	2.3	1.0
**NDI-5**	3	99	99	30	3.3	3.3	1.0
**NDI-6**	5	90	87	71	1.3	1.2	1.0
**NDI-7**	15	85	79	34	2.5	2.3	1.1
**NDI-8**	11	73	78	38	1.9	2.0	0.9
**NDI-9**	0	68	79	33	2.1	2.4	0.9
**NDI-10**	0	67	64	29	2.3	2.2	1.0
**NDI-11**	14	77	75	61	1.3	1.2	1.0
**NDI-12**	14	75	79	71	1.1	1.1	0.9

^a^ Bound ligand calculated as a difference from the unbound ligand, recovered with 50 mM KCl, 10% DMSO, 10% CH_3_CH_2_OH washing solution, and expressed as % of the amount initially loaded on the support. The errors associated with the % are within ± 2%.

## Supplementary Materials

Supplementary materials can be found at https://www.mdpi.com/1422-0067/21/6/1964/s1. Figure S1. Amount of the released ligands NDI-1-NDI-5 as a function of the volume of washing/releasing solutions. Figure S2. Amount of the released ligands NDI-6-NDI-12 as a function of the volume of washing/releasing solutions. Figure S3. Amount of the released ligands NDI-1-NDI-12 as a function of the volume of washing/releasing solutions. Figure S4. CD spectra of tel26, cmyc and ds27 in the absence and presence of increasing amount of NDI-9. Figure S5. Melting curves for tel26, cmyc and ds27 in the absence and presence of 6 equivalents of NDI-5 or NDI-9. Figure S6. CD spectra of tel26 in the absence and presence of increasing amounts of NDI-5 and melting curve for tel26/NDI-5 mixture (1:6). Figure S7. CD spectra of cmyc and ds27 in 10 mM Tris-HCl, 10% DMSO buffer (pH 7). Figure S8. Fluorescence spectra of NDI-5 in the absence and presence of tel26, cmyc and ds27. Figure S9. Fluorescence experiments for NDI-9. Additional data, i.e., HPLC purity data, ESI-MS spectra and ^1^H- and ^13^C-NMR characterization for the here synthesized NDIs, are reported in pages S10–S34.

## References

[1] Hänsel-Hertsch R., Spiegel J., Marsico G., Tannahill D., Balasubramanian S. (2018). Genome-wide mapping of endogenous G-quadruplex DNA structures by chromatin immunoprecipitation and high-throughput sequencing. Nat. Protoc..

[2] Balasubramanian S., Hurley L.H., Neidle S. (2011). Targeting G-quadruplexes in gene promoters: A novel anticancer strategy?. Nat. Rev. Drug Discov..

[3] Hänsel-Hertsch R., Di Antonio M., Balasubramanian S. (2017). DNA G-quadruplexes in the human genome: Detection, functions and therapeutic potential. Nat. Rev. Mol. Cell Biol..

[4] Biffi G., Tannahill D., Miller J., Howat W.J., Balasubramanian S. (2014). Elevated levels of G-quadruplex formation in human stomach and liver cancer tissues. PLoS ONE.

[5] Platella C., Riccardi C., Montesarchio D., Roviello G.N., Musumeci D. (2017). G-quadruplex-based aptamers against protein targets in therapy and diagnostics. Biochim. Biophys. Acta Gen. Subj..

[6] Musumeci D., Platella C., Riccardi C., Merlino A., Marzo T., Massai L., Messori L., Montesarchio D. (2016). A first-in-class and a fished out anticancer platinum compound: Cis-[PtCl_2_(NH_3_)_2_] and *cis*-[PtI_2_(NH_3_)_2_] compared for their reactivity towards DNA model systems. Dalt. Trans..

[7] Doria F., Nadai M., Costa G., Sattin G., Gallati C., Bergamaschi G., Moraca F., Alcaro S., Freccero M., Richter S.N. (2016). Extended naphthalene diimides with donor/acceptor hydrogen-bonding properties targeting G-quadruplex nucleic acids. Eur. J. Org. Chem..

[8] Arévalo-Ruiz M., Doria F., Belmonte-Reche E., De Rache A., Campos-Salinas J., Lucas R., Falomir E., Carda M., Pérez-Victoria J.M., Mergny J.L. (2017). Synthesis, binding properties, and differences in cell uptake of G-quadruplex ligands based on carbohydrate naphthalene diimide conjugates. Chem. Eur. J..

[9] Nadai M., Doria F., Di Antonio M., Sattin G., Germani L., Percivalle C., Palumbo M., Richter S.N., Freccero M. (2011). Naphthalene diimide scaffolds with dual reversible and covalent interaction properties towards G-quadruplex. Biochimie.

[10] Collie G.W., Promontorio R., Hampel S.M., Micco M., Neidle S., Parkinson G.N. (2012). Structural basis for telomeric G-quadruplex targeting by naphthalene diimide ligands. J. Am. Chem. Soc..

[11] Doria F., Nadai M., Zuffo M., Perrone R., Freccero M., Richter S.N. (2017). A red-NIR fluorescent dye detecting nuclear DNA G-quadruplexes: In vitro analysis and cell imaging. Chem. Commun..

[12] Street S.T.G., Chin D.N., Hollingworth G.J., Berry M., Morales J.C., Galan M.C. (2017). Divalent naphthalene diimide ligands display high selectivity for the human telomeric G-quadruplex in K^+^ buffer. Chem. Eur. J..

[13] Cuenca F., Greciano O., Gunaratnam M., Haider S., Munnur D., Nanjunda R., Wilson W.D., Neidle S. (2008). Tri-and tetra-substituted naphthalene diimides as potent G-quadruplex ligands. Bioorg. Med. Chem. Lett..

[14] Doria F., Nadai M., Sattin G., Pasotti L., Richter S.N., Freccero M. (2012). Water soluble extended naphthalene diimides as pH fluorescent sensors and G-quadruplex ligands. Org. Biomol. Chem..

[15] Salvati E., Doria F., Manoli F., D’Angelo C., Biroccio A., Freccero M., Manet I. (2016). A bimodal fluorescent and photocytotoxic naphthalene diimide for theranostic applications. Org. Biomol. Chem..

[16] Micco M., Collie G.W., Dale A.G., Ohnmacht S.A., Pazitna I., Gunaratnam M., Reszka A.P., Neidle S. (2013). Structure-based design and evaluation of naphthalene diimide G-quadruplex ligands as telomere targeting agents in pancreatic cancer cells. J. Med. Chem..

[17] Pirota V., Nadai M., Doria F., Richter S.N. (2019). Naphthalene diimides as multimodal G-quadruplex-selective ligands. Molecules.

[18] Nadai M., Cimino-Reale G., Sattin G., Doria F., Butovskaya E., Zaffaroni N., Freccero M., Palumbo M., Richter S.N., Folini M. (2015). Assessment of gene promoter G-quadruplex binding and modulation by a naphthalene diimide derivative in tumor cells. Int. J. Oncol..

[19] Marchetti C., Zyner K.G., Ohnmacht S.A., Robson M., Haider S.M., Morton J.P., Marsico G., Vo T., Laughlin-Toth S., Ahmed A.A. (2018). Targeting multiple effector pathways in pancreatic ductal adenocarcinoma with a G-quadruplex-binding small molecule. J. Med. Chem..

[20] Doria F., Oppi A., Manoli F., Botti S., Kandoth N., Grande V., Manet I., Freccero M. (2015). A naphthalene diimide dyad for fluorescence switch-on detection of G-quadruplexes. Chem. Commun..

[21] Doria F., Salvati E., Pompili L., Pirota V., D’Angelo C., Manoli F., Nadai M., Richter S.N., Biroccio A., Manet I. (2019). Dyads of G-quadruplex ligands triggering DNA damage response and tumour cell growth inhibition at sub-nM concentration. Chem. Eur. J..

[22] Tassinari M., Cimino-Reale G., Nadai M., Doria F., Butovskaya E., Recagni M., Freccero M., Zaffaroni N., Richter S.N., Folini M. (2018). Down-regulation of the androgen receptor by G-quadruplex ligands sensitizes castration-resistant prostate cancer cells to enzalutamide. J. Med. Chem..

[23] Zuffo M., Guedin A., Leriche E., Doria F., Pirota V., Gabelica V., Mergny J.L., Freccero M. (2018). More is not always better: Finding the right trade-off between affinity and selectivity of a G-quadruplex ligand. Nucleic Acids Res..

[24] Giorgi M.E., Agusti R., De Lederkremer R.M. (2014). Carbohydrate PEGylation, an approach to improve pharmacological potency. Beilstein J. Org. Chem..

[25] Zuffo M., Ladame S., Doria F., Freccero M. (2017). Tuneable coumarin-NDI dyads as G-quadruplex specific light-up probes. Sens. Actuators B Chem..

[26] Platella C., Musumeci D., Arciello A., Doria F., Freccero M., Randazzo A., Amato J., Pagano B., Montesarchio D. (2018). Controlled Pore Glass-based oligonucleotide affinity support: Towards High Throughput Screening methods for the identification of conformation-selective G-quadruplex ligands. Anal. Chim. Acta.

[27] Platella C., Musumeci D., Amato J., Randazzo A., Pagano B., Montesarchio D. (2019). Method for the Preparation of a Low Unspecific Binding-Support for Affinity Chromatography and/or on-Line Synthesis of Oligonucleotides. Italian Patent.

[28] Amato J., Platella C., Iachettini S., Zizza P., Musumeci D., Cosconati S., Pagano A., Novellino E., Biroccio A., Randazzo A. (2019). Tailoring a lead-like compound targeting multiple G-quadruplex structures. Eur. J. Med. Chem..

[29] Zuffo M., Stucchi A., Campos-Salinas J., Cabello-Donayre M., Martínez-García M., Belmonte-Reche E., Pérez-Victoria J.M., Mergny J.L., Freccero M., Morales J.C. (2019). Carbohydrate-naphthalene diimide conjugates as potential antiparasitic drugs: Synthesis, evaluation and structure-activity studies. Eur. J. Med. Chem..

[30] Thompson A.C., Grimm H.M., Gray Bé A., McKnight K.J., Reczek J.J. (2015). Efficient bromination of naphthalene dianhydride and microwave-assisted synthesis of core-brominated naphthalene diimides. Synth. Commun..

[31] Doria F., Manet I., Grande V., Monti S., Freccero M. (2013). Water-soluble naphthalene diimides as singlet oxygen sensitizers. J. Org. Chem..

[32] Musumeci D., Amato J., Zizza P., Platella C., Cosconati S., Cingolani C., Biroccio A., Novellino E., Randazzo A., Giancola C. (2017). Tandem application of ligand-based virtual screening and G4-OAS assay to identify novel G-quadruplex-targeting chemotypes. Biochim. Biophys. Acta Gen. Subj..

[33] Musumeci D., Amato J., Randazzo A., Novellino E., Giancola C., Montesarchio D., Pagano B. (2014). G-quadruplex on Oligo Affinity Support (G4-OAS): An easy affinity chromatography-based assay for the screening of G-quadruplex ligands. Anal. Chem..

[34] Petraccone L., Spink C., Trent J.O., Garbett N.C., Mekmaysy C.S., Giancola C., Chaires J.B. (2011). Structure and stability of higher-order human telomeric quadruplexes. J. Am. Chem. Soc..

[35] Sun D., Hurley L.H. (2009). The importance of negative superhelicity in inducing the formation of G-quadruplex and i-motif structures in the c-Myc promoter: Implications for drug targeting and control of gene expression. J. Med. Chem..

[36] Mathad R.I., Hatzakis E., Dai J., Yang D. (2011). C-MYC promoter G-quadruplex formed at the 5′-end of NHE III1 element: Insights into biological relevance and parallel-stranded G-quadruplex stability. Nucleic Acids Res..

[37] Kypr J., Kejnovská I., Renčiuk D., Vorlíčková M. (2009). Circular dichroism and conformational polymorphism of DNA. Nucleic Acids Res..

[38] Parkinson G.N., Cuenca F., Neidle S. (2008). Topology conservation and loop flexibility in quadruplex-drug recognition: Crystal structures of inter-and intramolecular telomeric DNA quadruplex-drug complexes. J. Mol. Biol..

[39] Giancola C., Pagano B. (2013). Energetics of ligand binding to G-quadruplexes. Top Curr. Chem..

[40] Parkinson G.N., Ghosh R., Neidle S. (2007). Structural basis for binding of porphyrin to human telomeres. Biochemistry.

[41] Monchaud D., Allain C., Bertrand H., Smargiasso N., Rosu F., Gabelica V., De Cian A., Mergny J.L., Teulade-Fichou M.P. (2008). Ligands playing musical chairs with G-quadruplex DNA: A rapid and simple displacement assay for identifying selective G-quadruplex binders. Biochimie.

[42] Platella C., Guida S., Bonmassar L., Aquino A., Bonmassar E., Ravagnan G., Montesarchio D., Roviello G.N., Musumeci D., Fuggetta M.P. (2017). Antitumour activity of resveratrol on human melanoma cells: A possible mechanism related to its interaction with malignant cell telomerase. Biochim. Biophys. Acta Gen. Subj..

[43] Cheng M., Modi C., Cookson J.C., Hutchinson I., Heald R.A., McCarroll A.J., Missailidis S., Tanious F., Wilson W.D., Mergny J. (2008). Antitumor polycyclic acridines. 20.1 Search for DNA quadruplex binding selectivity in a series of 8,13-dimethylquino [4,3,2-kl] acridinium salts: Telomere-targeted agents. J. Med. Chem..

[44] Ferreira R., Aviñó A., Mazzini S., Eritja R. (2012). Synthesis, DNA-binding and antiproliferative properties of acridine and 5-methylacridine derivatives. Molecules.

[45] Pagano B., Fotticchia I., De Tito S., Mattia C.A., Mayol L., Novellino E., Randazzo A., Giancola C. (2010). Selective binding of distamycin A derivative to G-quadruplex structure [d(TGGGGT)]_4_. J. Nucleic Acids.

[46] Pagano B., Virno A., Mattia C.A., Mayol L., Randazzo A., Giancola C. (2008). Targeting DNA quadruplexes with distamycin A and its derivatives: An ITC and NMR study. Biochimie.

[47] Chen W., Hahn W.C. (2003). SV40 early region oncoproteins and human cell transformation. Histol. Histopathol..

