# Supplementary material for: Trifunctionalized Naphthalene Diimides and Dimeric Analogues as G-Quadruplex-Targeting Anticancer Agents Selected by Affinity Chromatography
Source: Int J Mol Sci. 2020 Mar 13;21(6):1964. doi: 10.3390/ijms21061964 (PMC7139804; doi:10.3390/ijms21061964)

Supplementary Information

to the manuscript:

Trifunctionalized naphthalene diimides and dimeric analogues as G-quadruplex-targeting anticancer agents selected by affinity chromatography

Chiara Platella,[‡,a] Valentina Pirota,[‡,b] Domenica Musumeci,[a] Federica Rizzi,[b] Sara Iachettini,[c] Pasquale Zizza,[c] Annamaria Biroccio,[c] Mauro Freccero,[b] Daniela Montesarchio,[a,*] Filippo Doria[b,*]

[a]*Department of Chemical Sciences, University of Naples Federico II, 80126 Naples, Italy*

[b]*Department of Chemistry, University of Pavia, 27100 Pavia, Italy*

[c]*Oncogenomic and Epigenetic Unit, IRCCS - Regina Elena National Cancer Institute, 00144 Rome, Italy*

Contents **Page**

Figure S1. Amount of the released ligands **NDI-1**-**NDI-5** as a function of the volume of washing/releasing solutions. S1

Figure S2. Amount of the released ligands **NDI-6**-**NDI-12** as a function of the volume of washing/releasing solutions. S2

Figure S3. Amount of the released ligands **NDI-1**-**NDI-12** as a function of the volume of washing/releasing solutions. S3

Figure S4. CD spectra of tel26, cmyc and ds27 in the absence and presence of increasing amount of **NDI-9**. S4

Figure S5. Melting curves for tel26, cmyc and ds27 in the absence and presence of 6 equivalents of **NDI-5** or **NDI-9**. S5

Figure S6. CD spectra of tel26 in the absence and presence of increasing amounts of **NDI-5** and melting curve for tel26/**NDI-5** mixture (1:6). S6

Figure S7. CD spectra of cmyc and ds27 in 10 mM Tris-HCl, 10% DMSO buffer (pH 7). S7

Figure S8. Fluorescence spectra of **NDI-5** in the absence and presence of tel26, cmyc and ds27. S8

Figure S9. Fluorescence experiments for **NDI-9**. S9

HPLC purity data for the here synthesized NDIs. S10-S13

ESI-MS data (m/z) for the here synthesized NDIs S14-S23

^{1}H- and ^{13}C-NMR characterization for the here synthesized NDIs. S24-S34

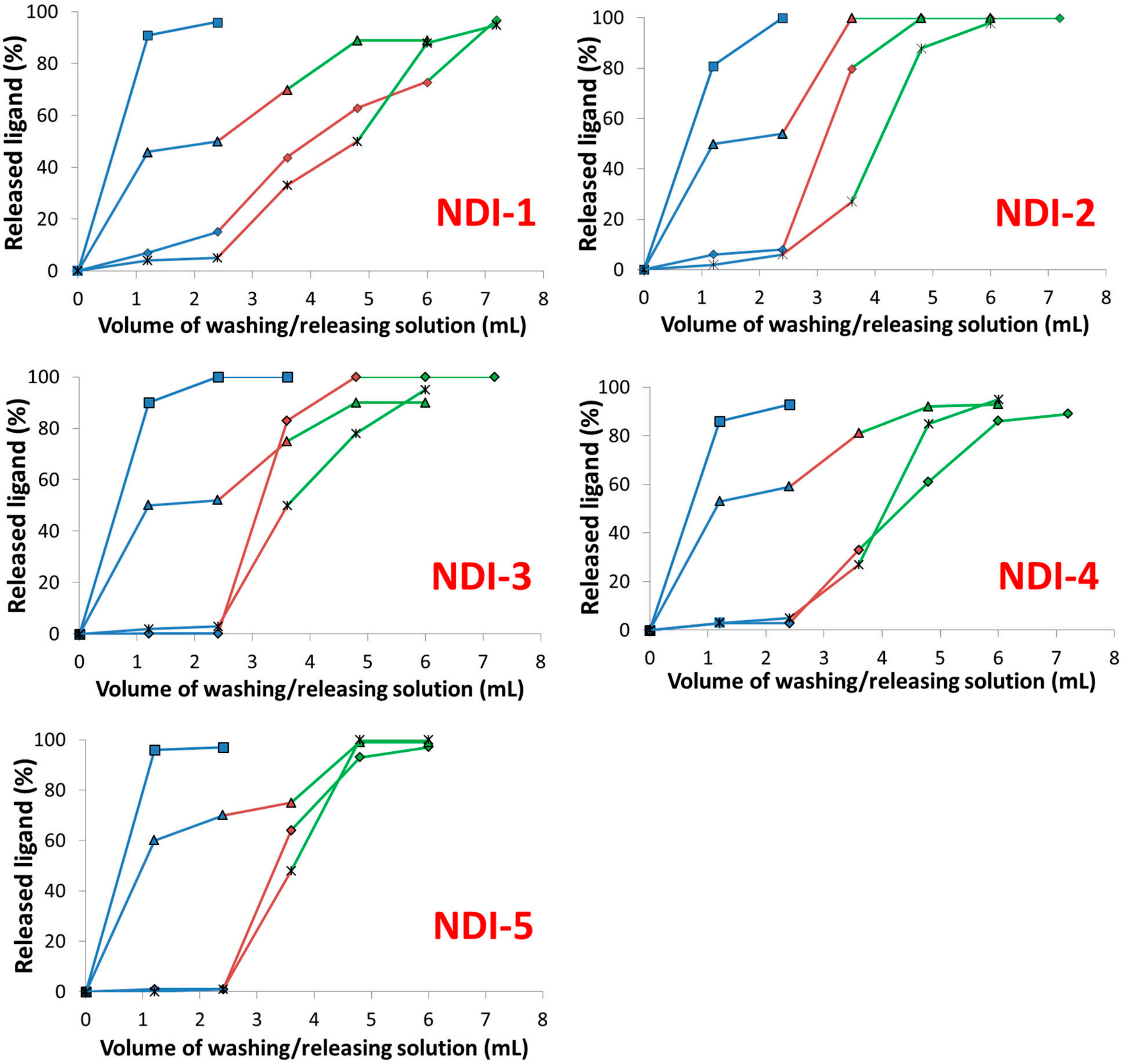

Figure S1. Amount of the released ligands **NDI-1**-**NDI-5**, expressed as percentage of the quantity loaded on nude CPG (-■-), CPG-tel26 (-♦-), CPG-cmyc (-∗-) and CPG-ds27 (-▲-) as a function of the volume of the washing solution 50 mM KCl, 10% DMSO, 10% CH_3CH_2OH (blue line) and the releasing solutions 2.5 M $CaCl_2$, 15% DMSO (red line) or pure DMSO (green line). The errors associated with the % are within ± 2%.

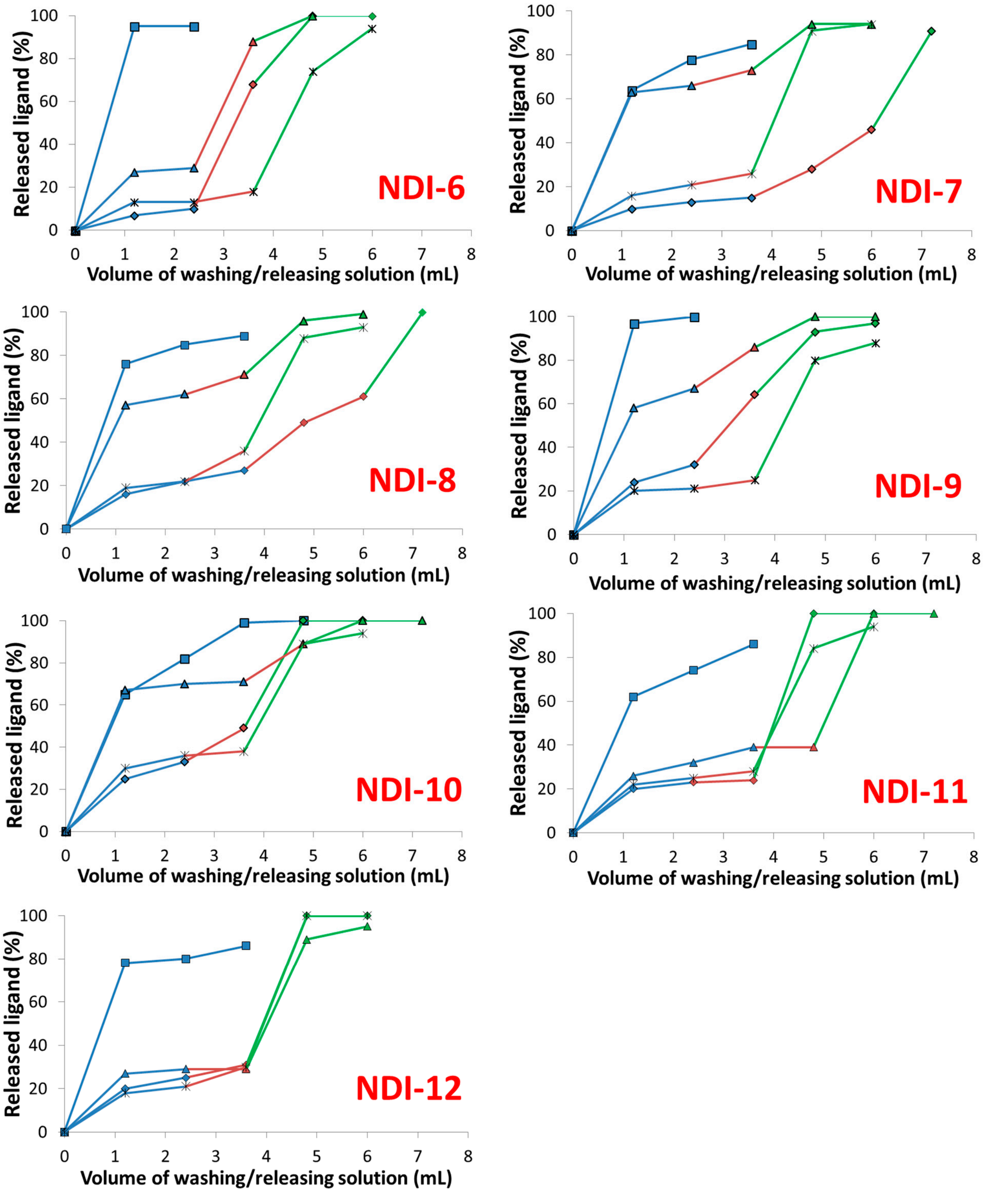

Figure S2. Amount of the released ligands **NDI-6-NDI-12**, expressed as percentage of the quantity loaded on nude CPG (-■-), CPG-tel26 (-♦-), CPG-cmyc (-∗-) and CPG-ds27 (-▲-) as a function of the volume of the washing solution 50 mM KCl, 10% DMSO, 10% CH_3CH_2OH (blue line) and the releasing solutions 2.5 M $CaCl_2$, 15% DMSO (red line) or pure DMSO (green line). The errors associated with the % are within ± 2%.

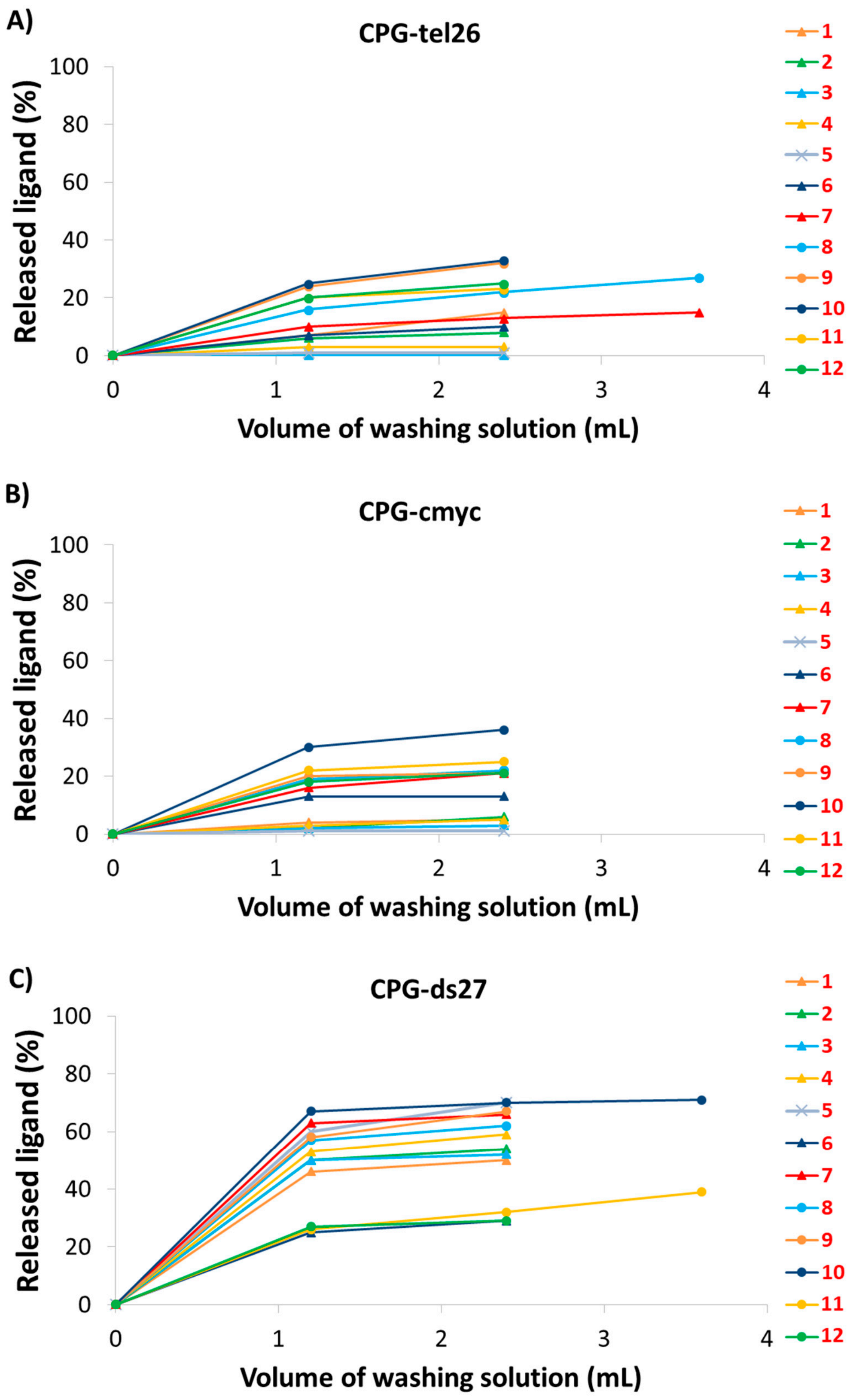

Figure S3. Amount of the released ligands **NDI-1-NDI-12**, expressed as percentage of the quantity loaded on A) CPG-tel26, B) CPG-cmyc and C) CPG-ds27 as a function of the volume of the washing solution (50 mM KCl, 10% DMSO, 10% CH_3CH_2OH). The errors associated with the % are within ± 2%.

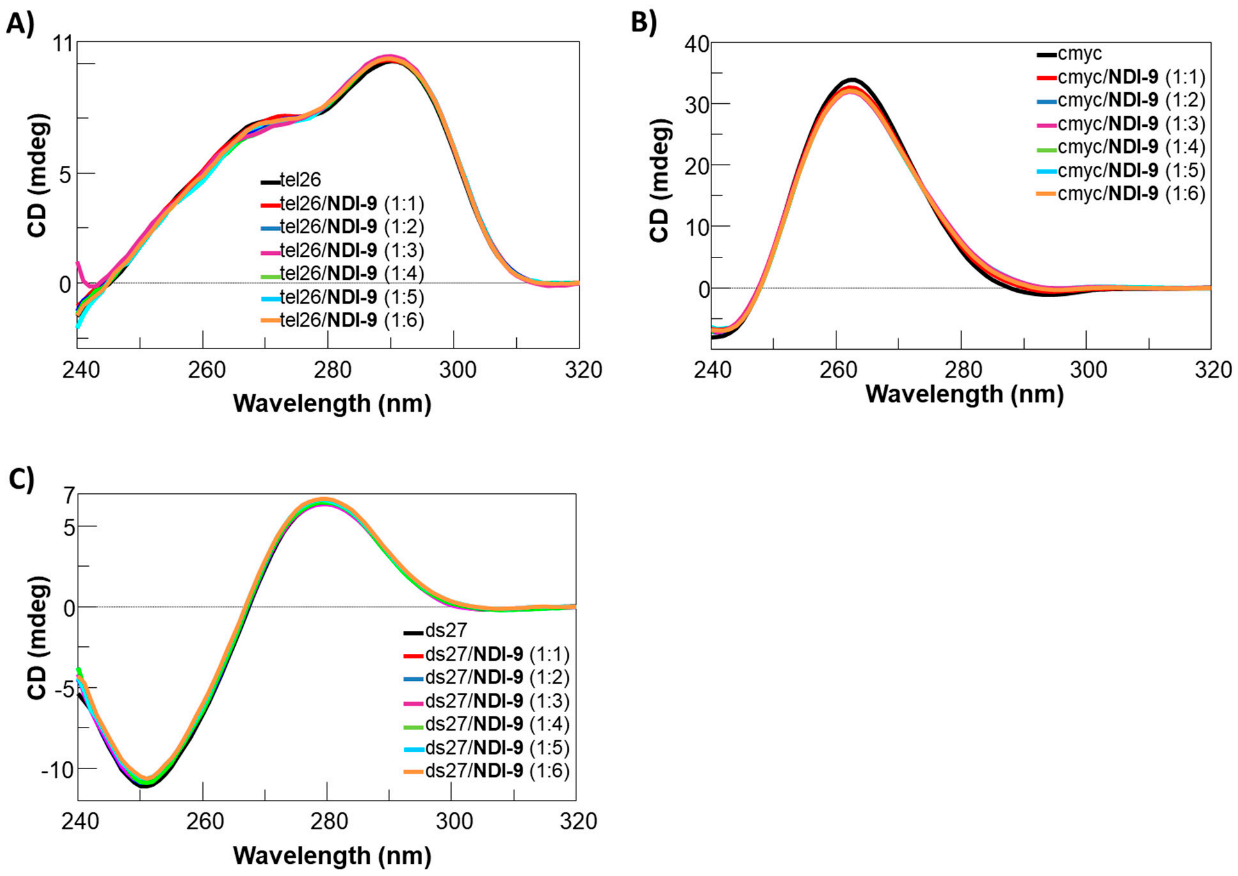

Figure S4. CD spectra of 2 μM solutions of: A) tel26, B) cmyc and C) ds27 in 20 mM KCl, 5 mM KH_2PO_4, 10% DMSO buffer (pH 7) in the absence and presence of increasing amount of **NDI-9** (up to 6 equivalents).

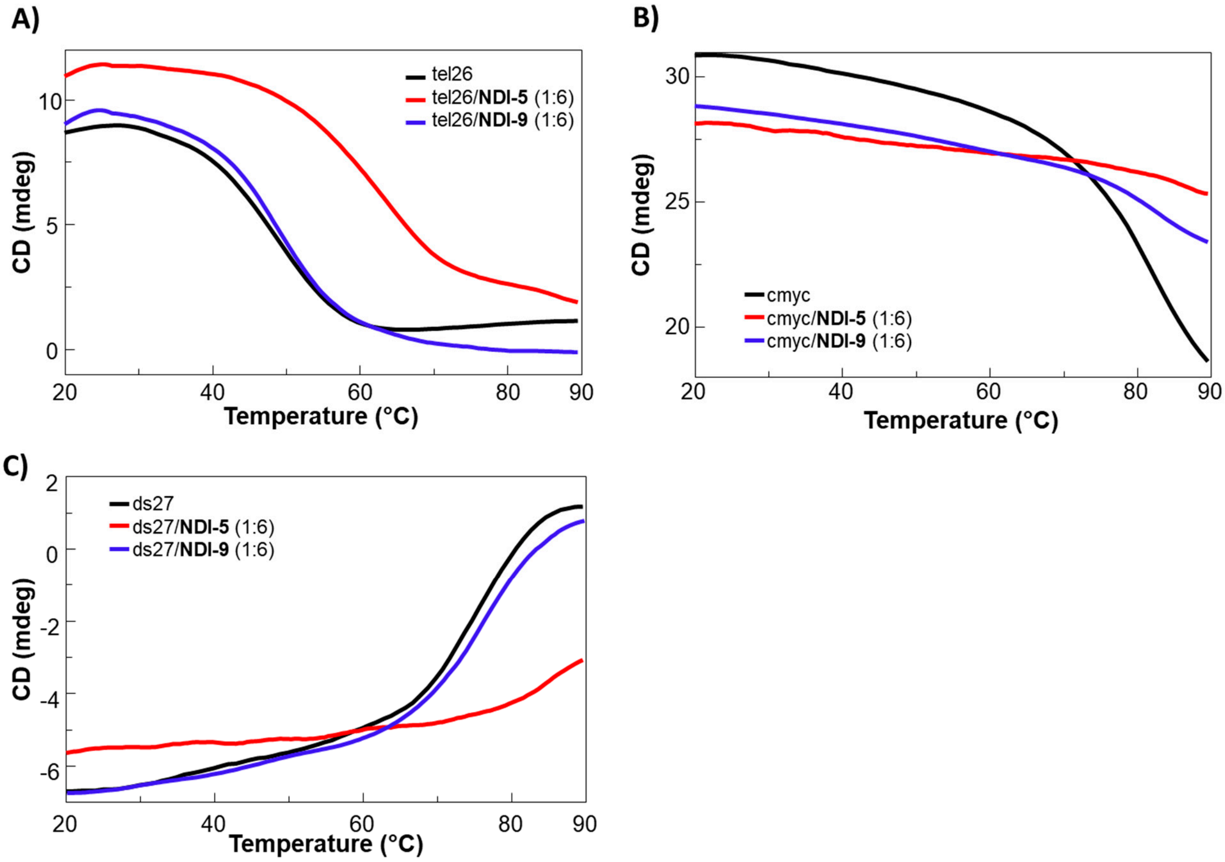

Figure S5. Melting curves for A) tel26, B) cmyc and C) ds27 in 20 mM KCl, 5 mM KH_2PO_4, 10% DMSO buffer (pH 7) in the absence (black lines) and presence of 6 equivalents of **NDI-5** (red lines) or **NDI-9** (blue lines) recorded at 290, 263 and 251 nm, respectively.

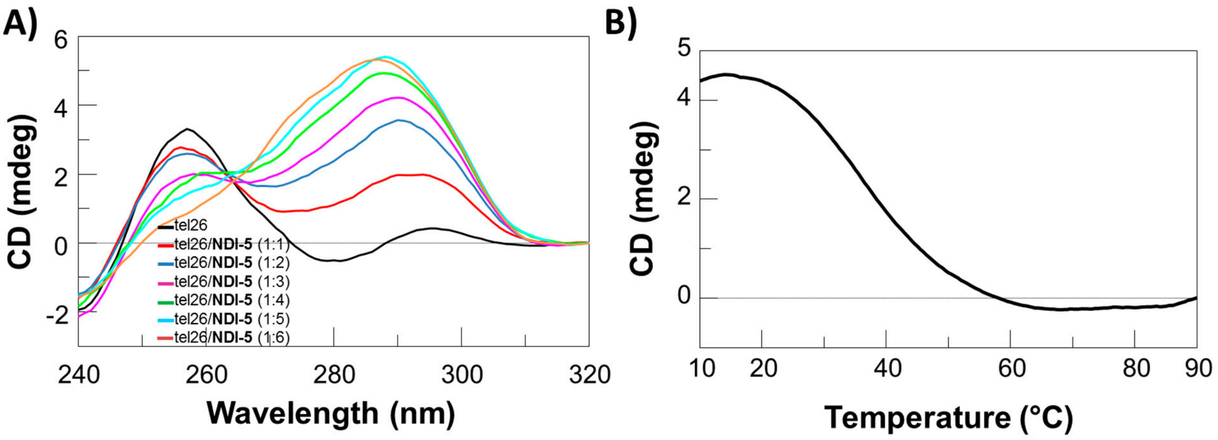

Figure S6. CD spectra of A) tel26 20 µM solution in 10 mM Tris-HCl, 10% DMSO buffer (pH 7), in the absence and presence of increasing amounts of **NDI-5** (up to 6 equivalents), and related melting curve B) for tel26/**NDI-5** mixture (1:6) recorded at 290 nm.

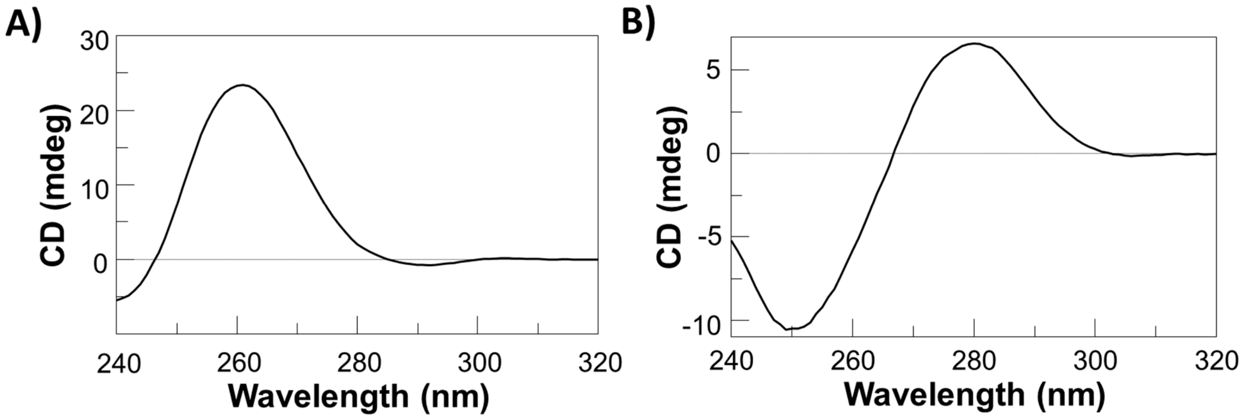

Figure S7. CD spectra of: A) cmyc and B) ds27, each 20 μM solutions in 10 mM Tris-HCl, 10% DMSO buffer (pH 7).

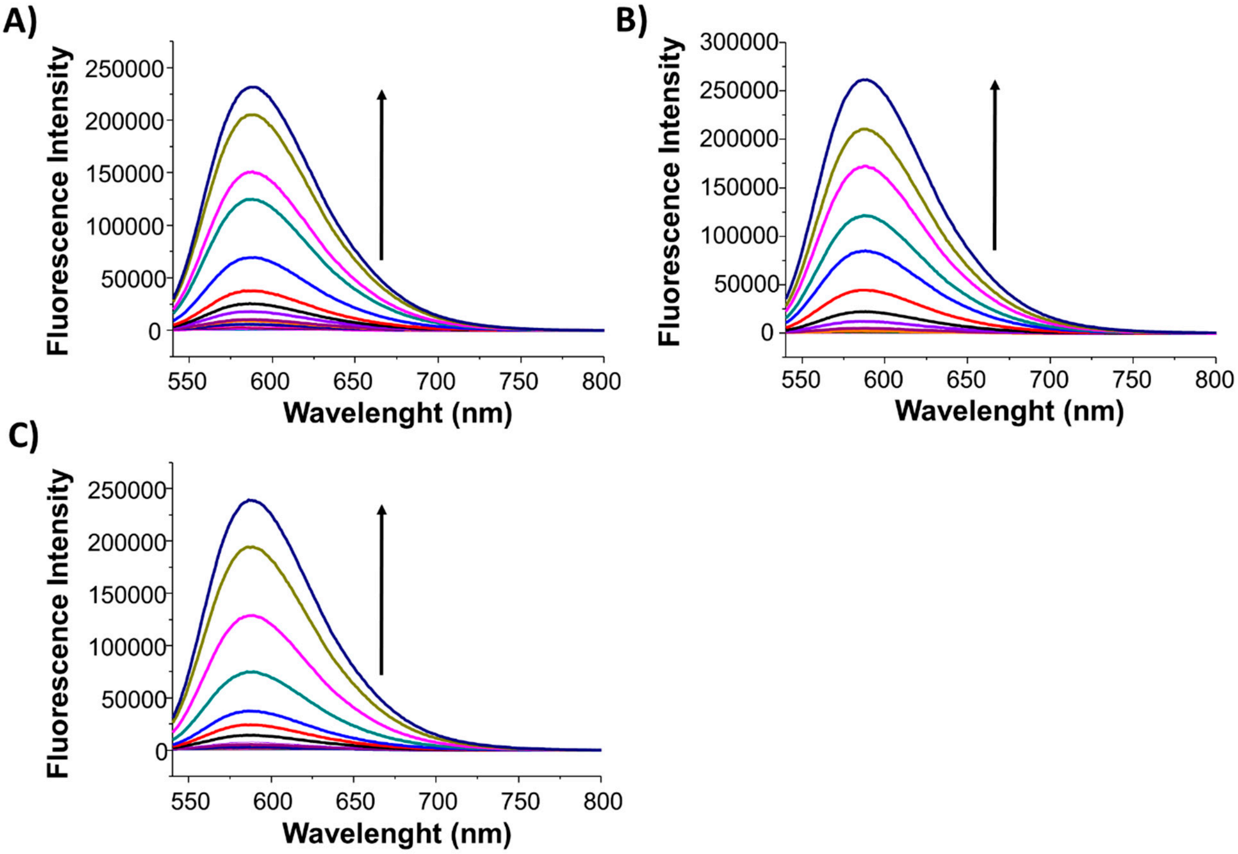

Figure S8. Fluorescence spectra of **NDI-5** in the absence and presence of: A) tel26, B) cmyc and C) ds27. The total molar concentration ([ligand] + [DNA]) was kept constant at 2 μM. The experiments were performed in 20 mM KCl, 5 mM KH_2PO_4, 10% DMSO buffer (pH 7). The excitation wavelength was 526 nm. The arrows indicate the increasing NDI concentration.

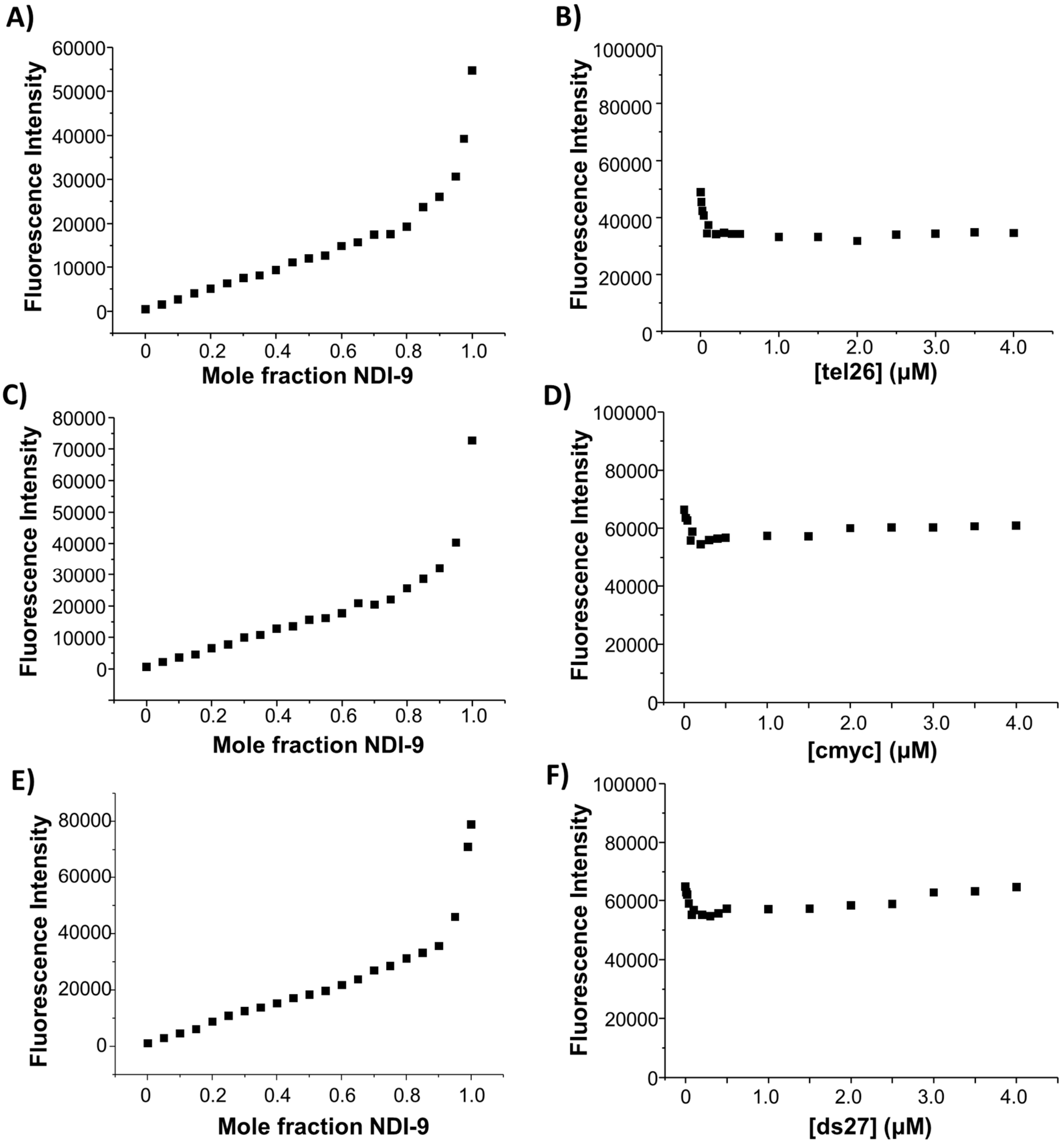

Figure S9. Fluorescence experiments for **NDI-9**. Left: Job plot analyses for **NDI-9** incubated with: A) tel26, C) cmyc and E) ds27. The total molar concentration ([ligand] + [DNA]) was kept constant at 2 μM. Right: Dependence of the fluorescence intensity for **NDI-9** as a function of: B) tel26 G4, D) cmyc G4 and F) ds27 hairpin duplex concentration. The experiments were performed in 20 mM KCl, 5 mM KH_2PO_4, 10% DMSO buffer (pH 7). The excitation wavelength was 518 nm and the here reported fluorescence intensity was taken at 584 nm.

HPLC purity data for the here synthesized NDIs

HPLC analyses were performed using an Agilent system SERIES 1260 with XBridge® BEH C18 column (2.5 µm, 4.6 x 50 mm). The method used to analyse the pure compounds is reported in the experimental section.

NDI-4 (t_R = 4.71 min, Area = 99.9%)

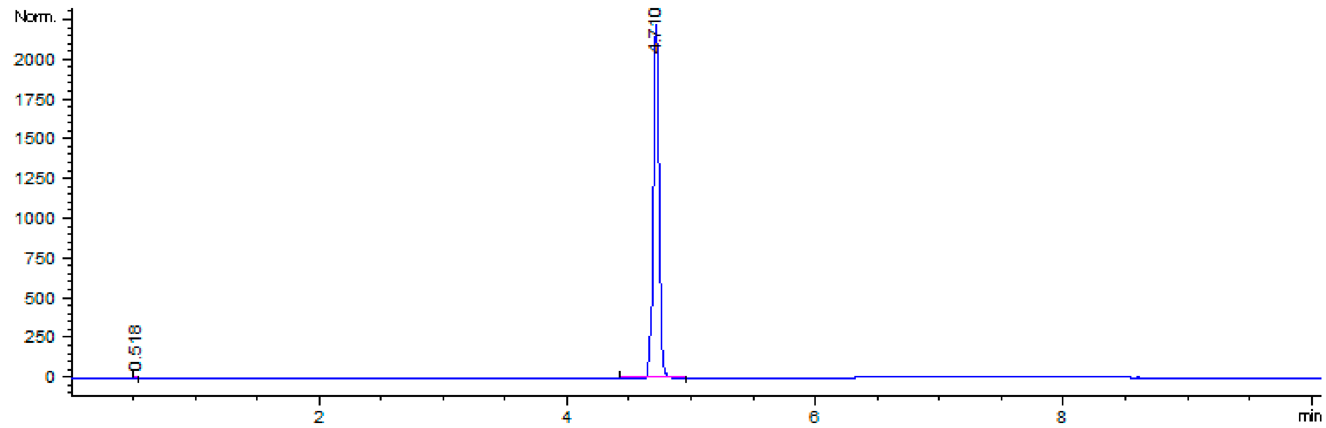

NDI-5 (t_R = 5.25 min, Area = 97.2%)

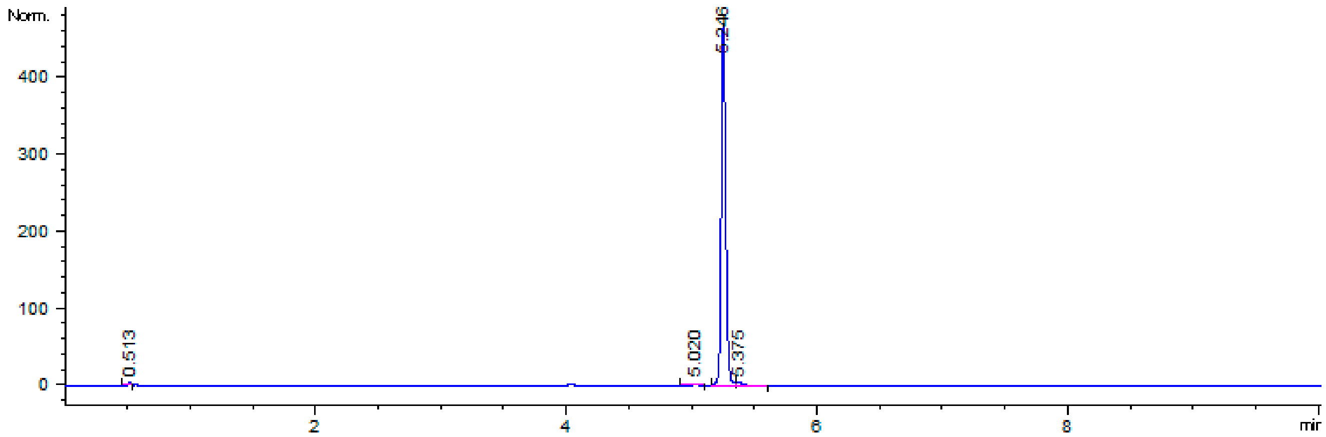

NDI-16 (t_R = 5.27 min, Area = 99.0%)

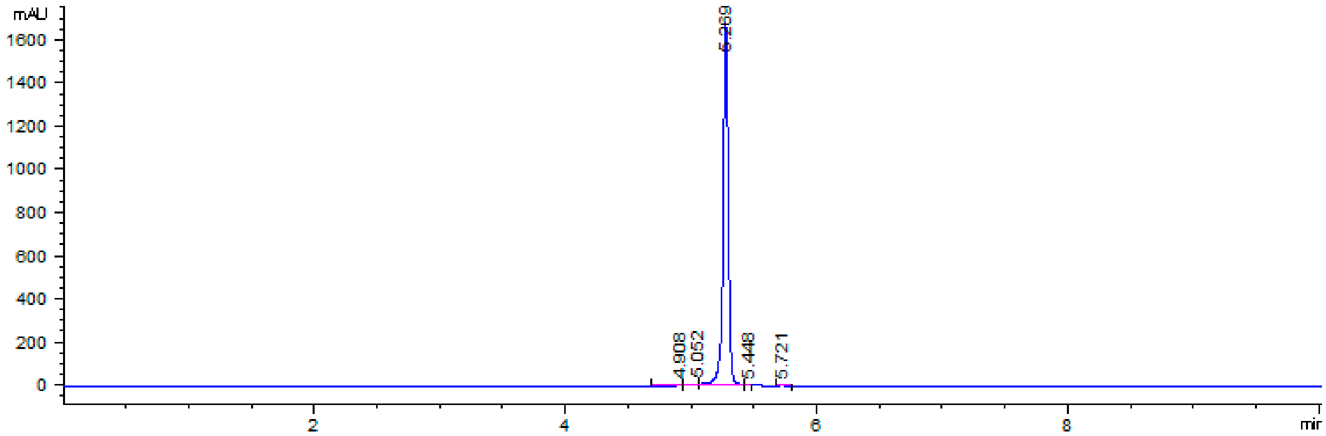

NDI-18 (t_R = 8.08 min, Area = 94.4%)

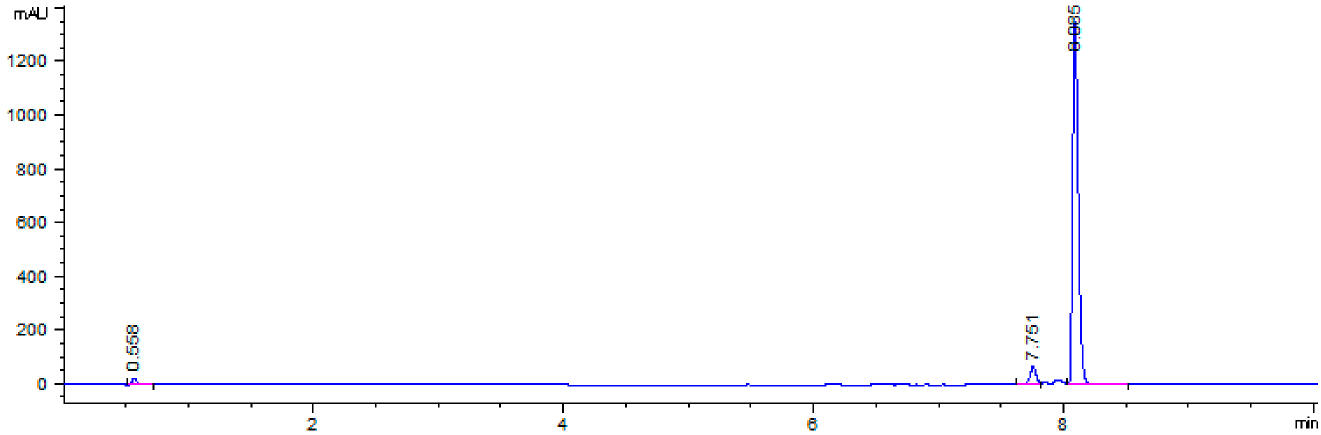

NDI-7 (t_R = 6.49 min, Area = 97.4%)

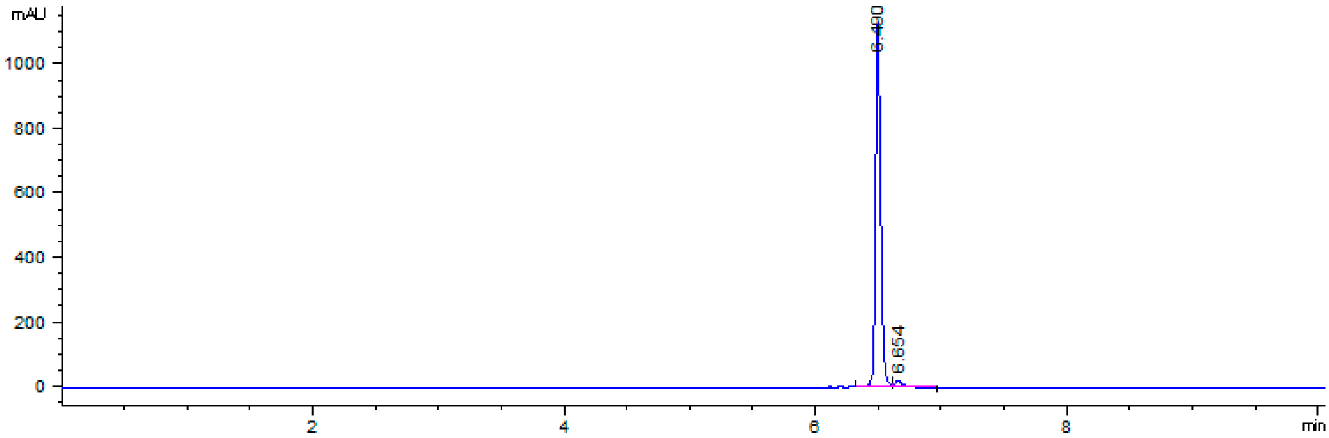

NDI-8 (t_R = 6.57 min, Area = 94.6%)

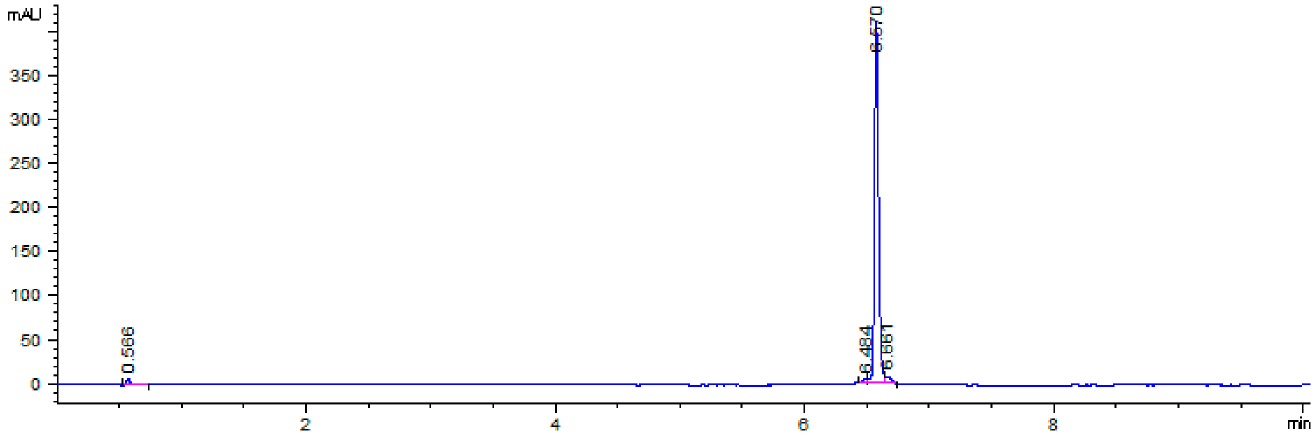

NDI-9 (t_R = 6.66 min, Area = 95.1%)

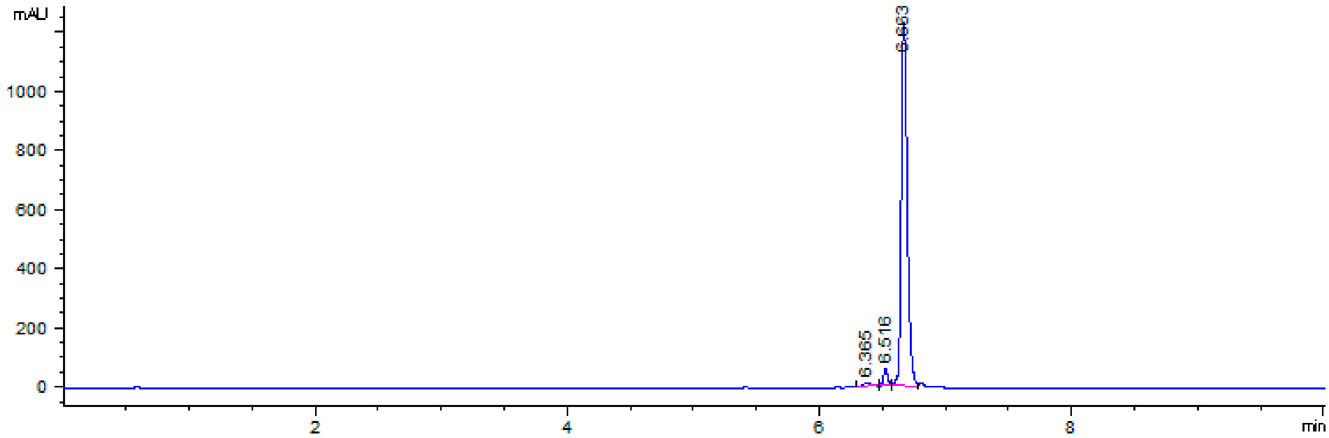

NDI-10 (t_R = 6.50 min, Area = 97.1%)

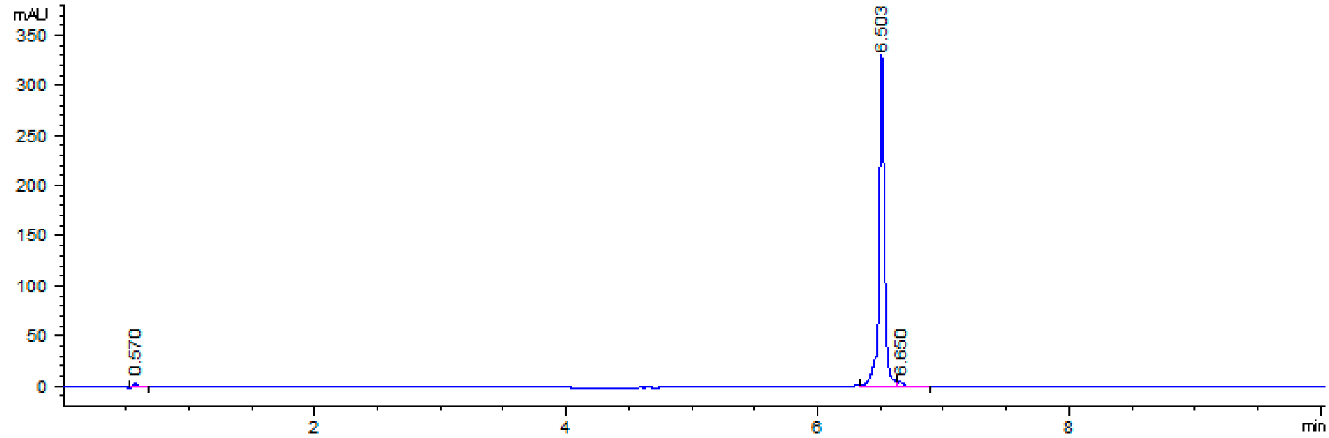

NDI-11 (t_R = 7.13 min, Area = 96.6%)

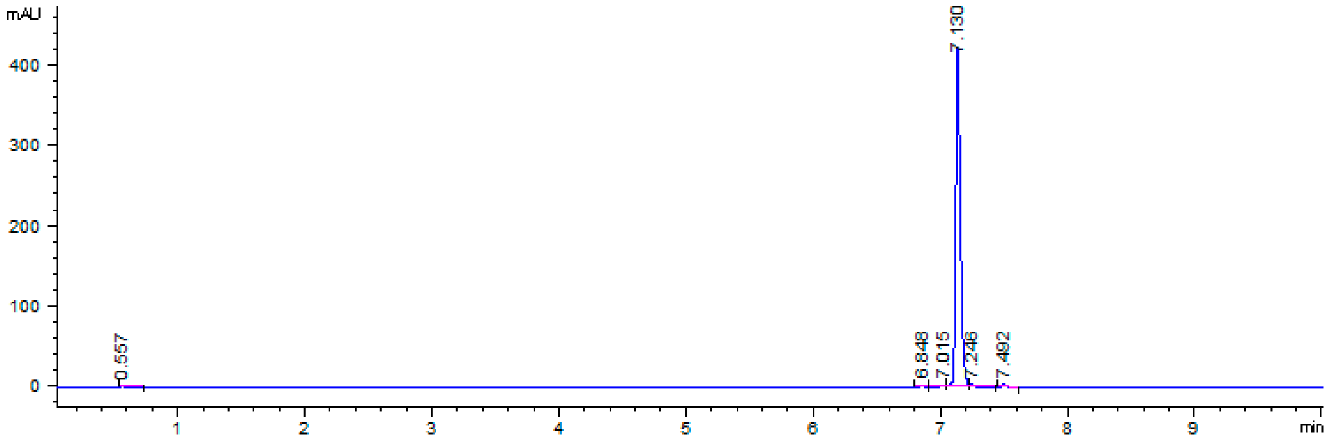

NDI-12 (t_R = 7.17 min, Area = 95.1%)

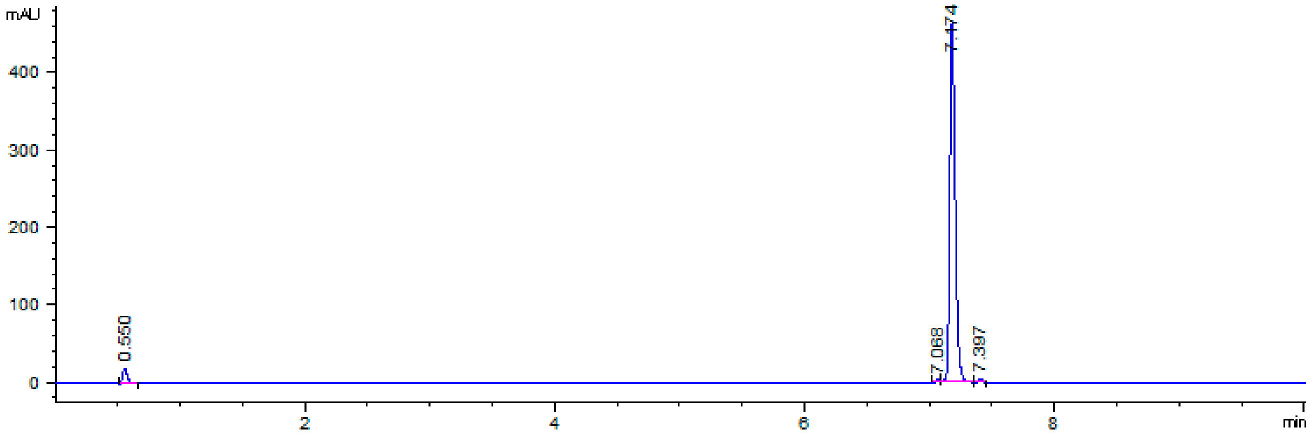

NDI-6 (t_R = 5.47 min, Area = 95.4%)

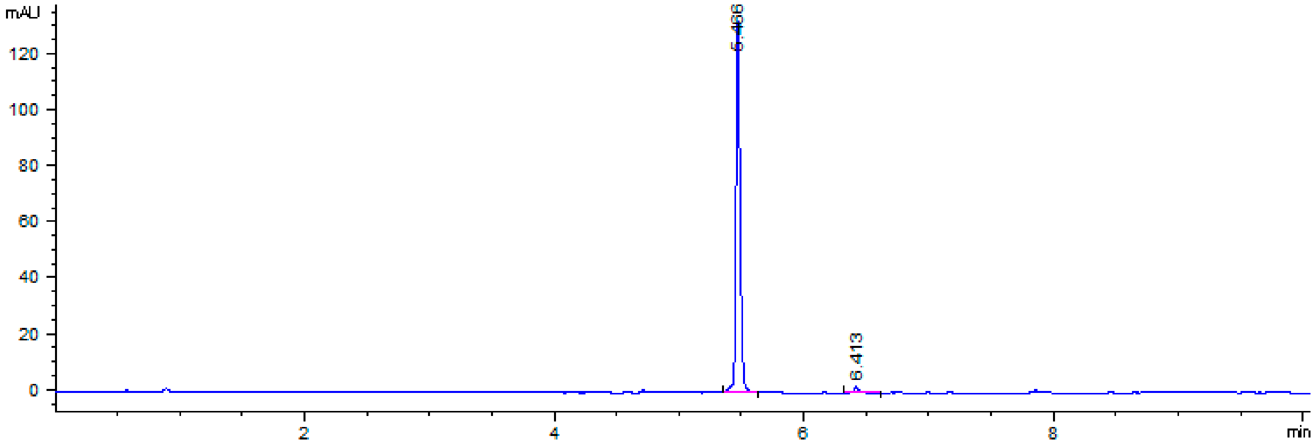

ESI-MS data (m/z) for the here synthesized NDIs

NDI-4

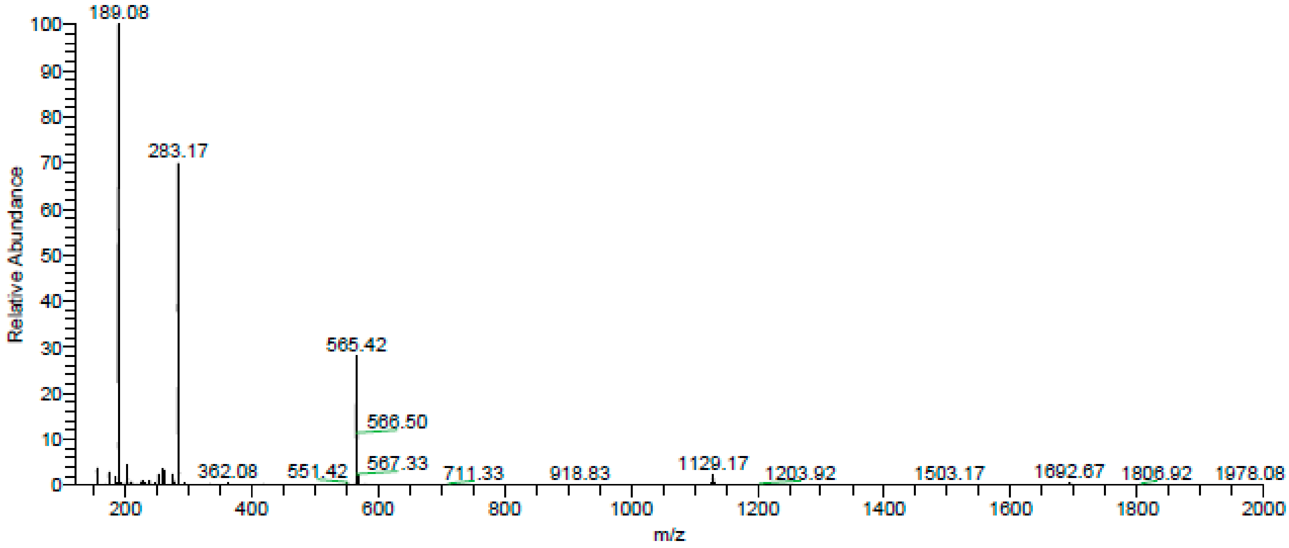

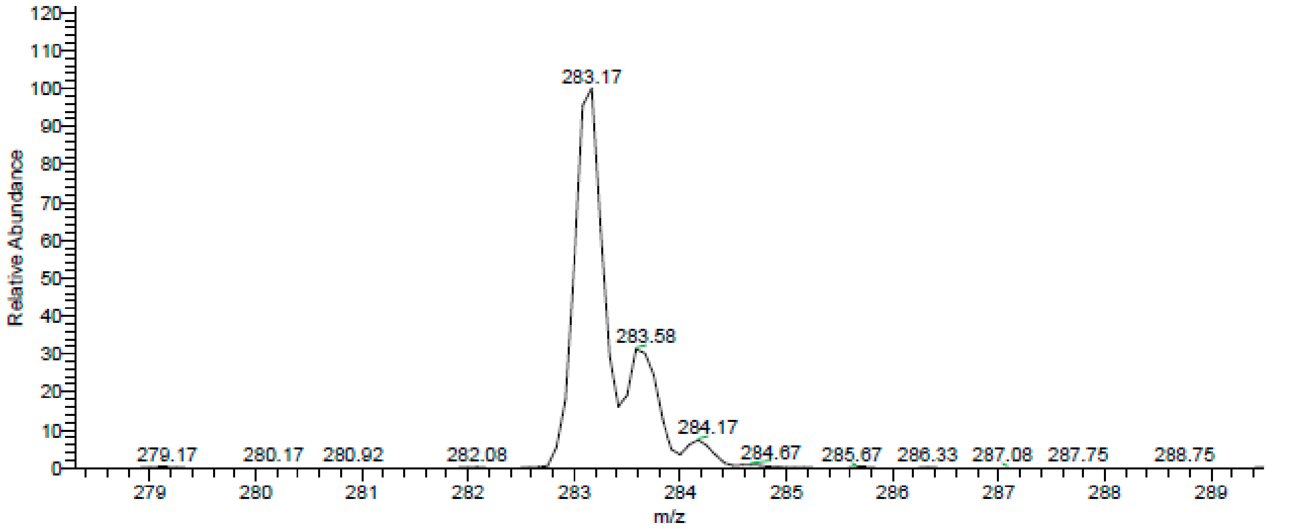

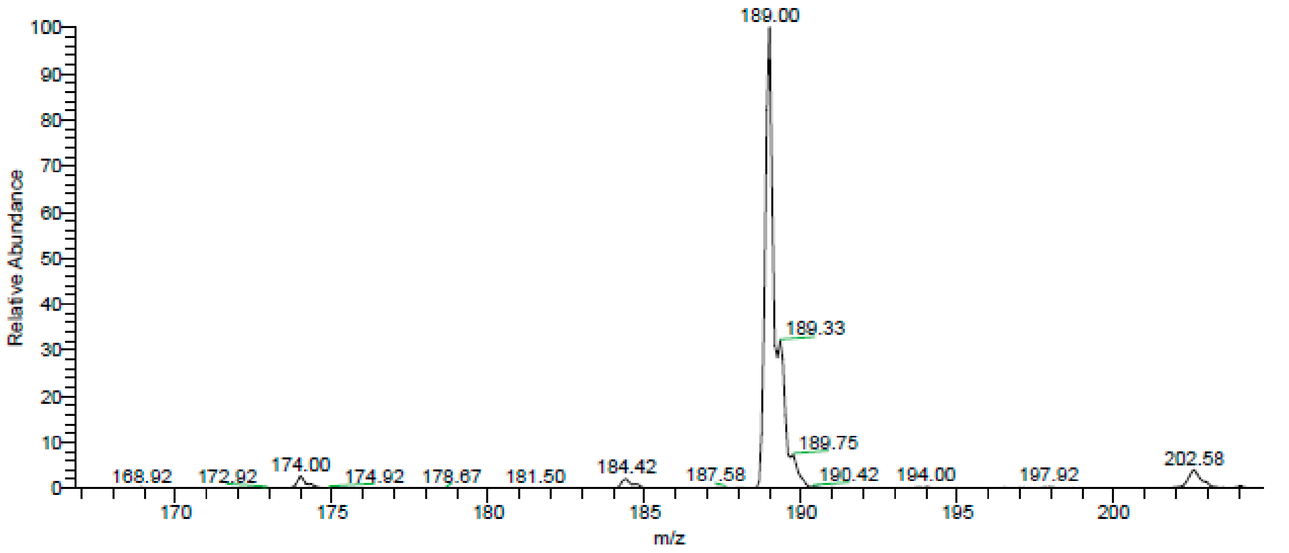

NDI-5

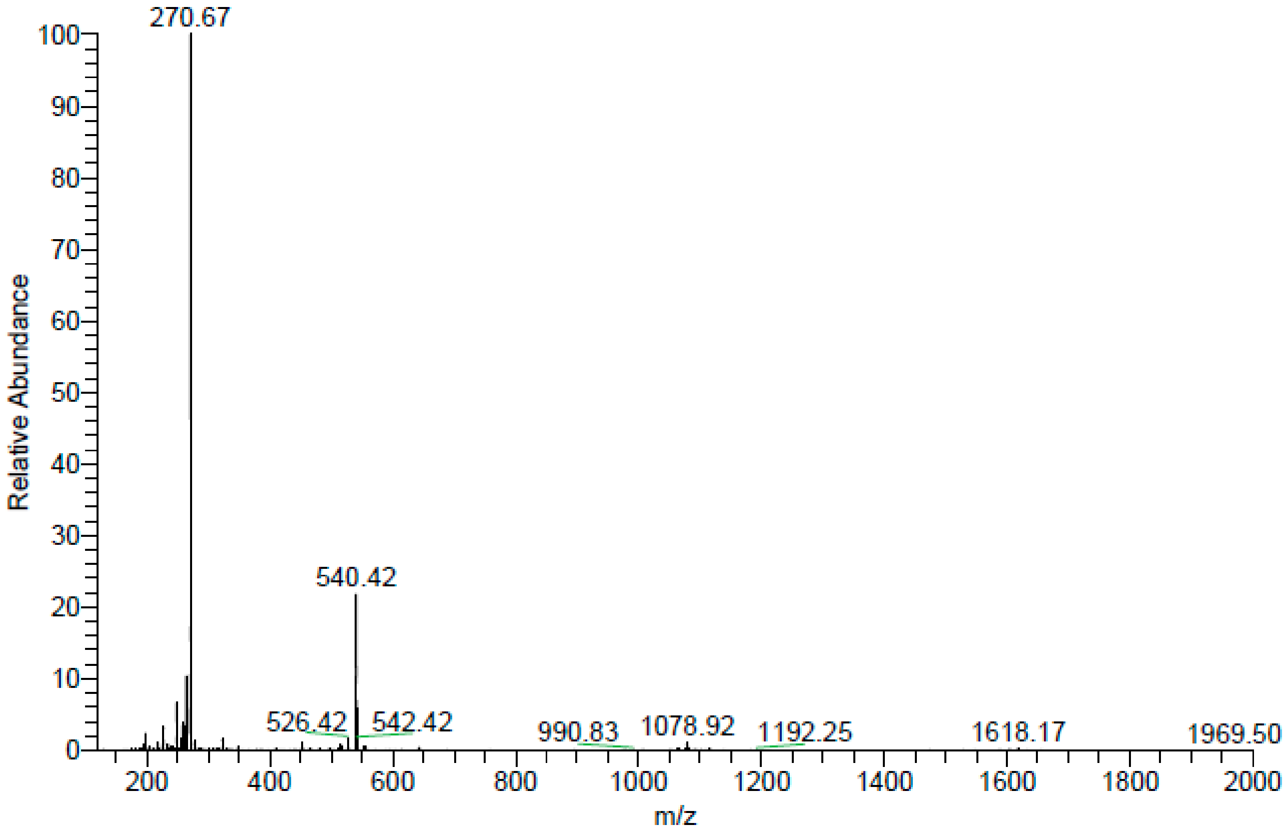

270.67
540.42
526.42
542.42
990.83
1078.92
1192.25
1618.17
1969.50
Relative Abundance
m/z

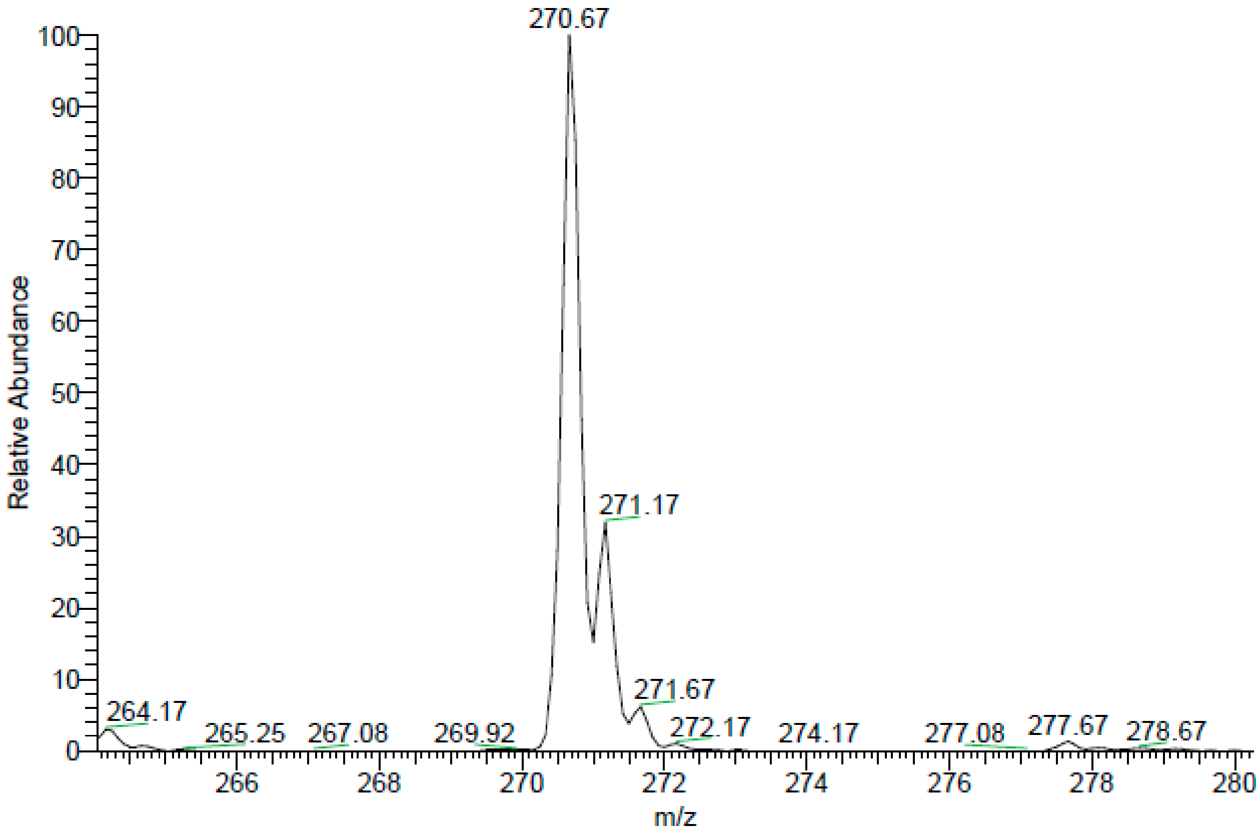

270.67
271.17
271.67
272.17
264.17
265.25
267.08
269.92
274.17
277.08
277.67
278.67
Relative Abundance
m/z

NDI-18

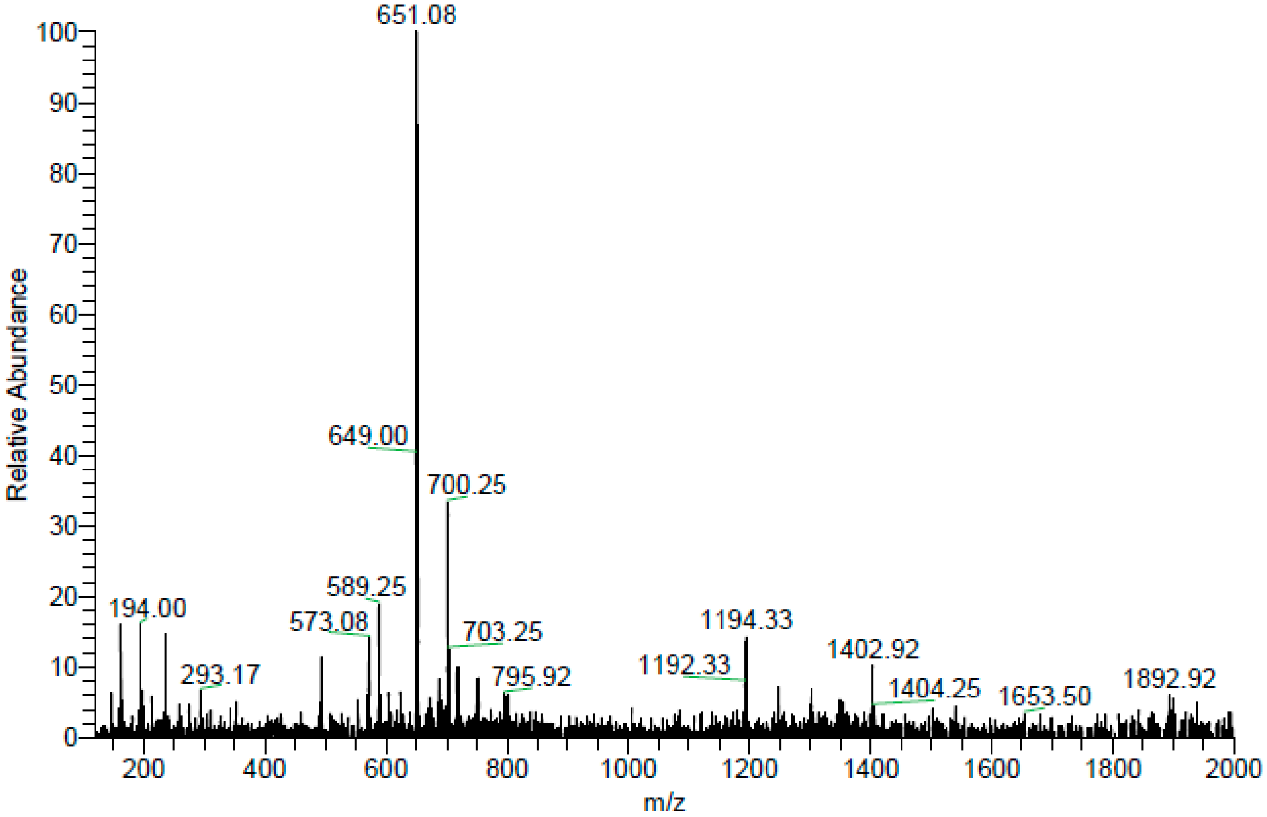
651.08
649.00
700.25
589.25
573.08
703.25
194.00
293.17
795.92
1194.33
1192.33
1402.92
1404.25
1653.50
1892.92
Relative Abundance
m/z

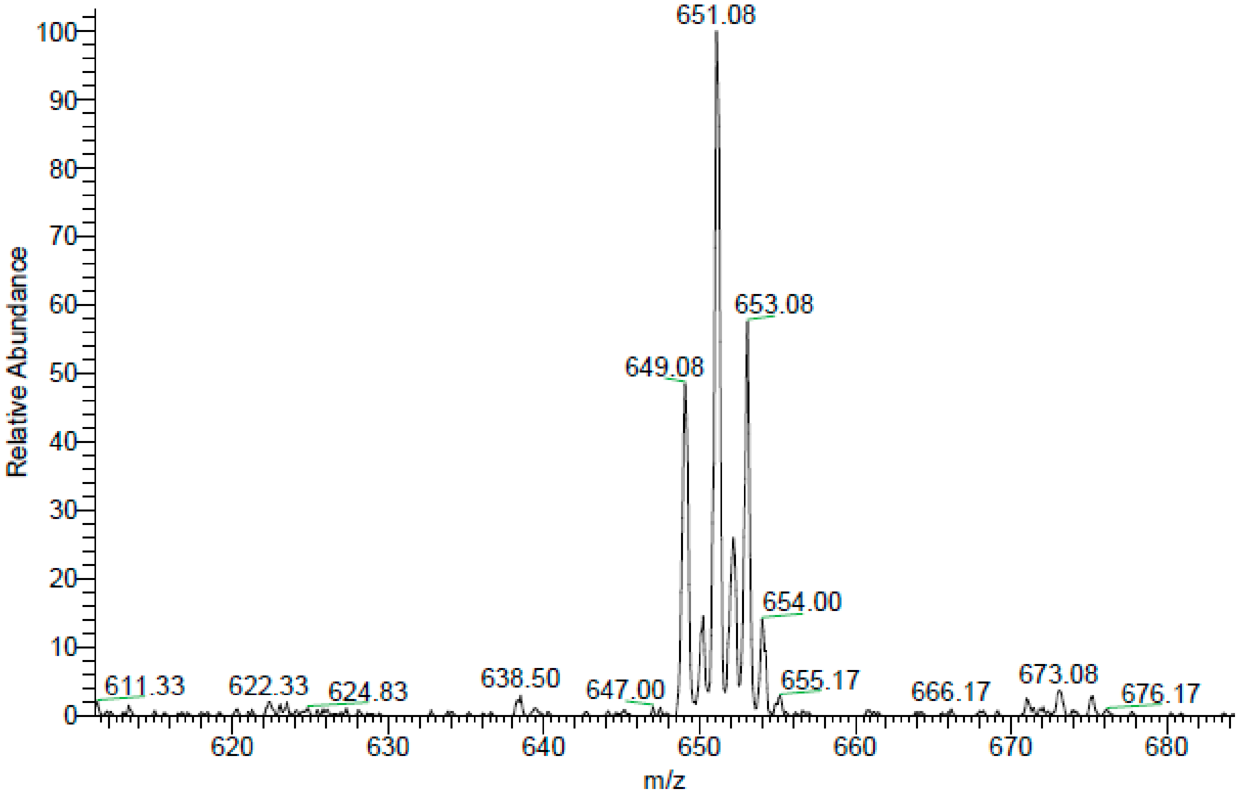
651.08
653.08
649.08
654.00
655.17
611.33
622.33
624.83
638.50
647.00
666.17
673.08
676.17
Relative Abundance
m/z

NDI-7

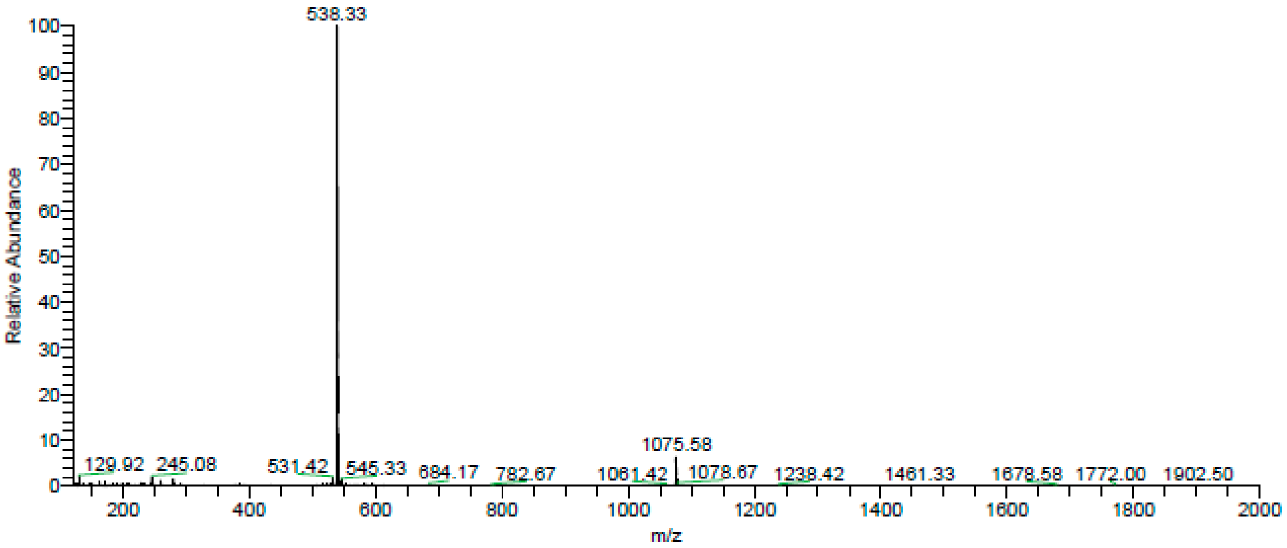
538.33
1075.58
129.92 245.08 531.42 545.33 684.17 782.67 1061.42 1078.67 1238.42 1461.33 1678.58 1772.00 1902.50
Relative Abundance
m/z

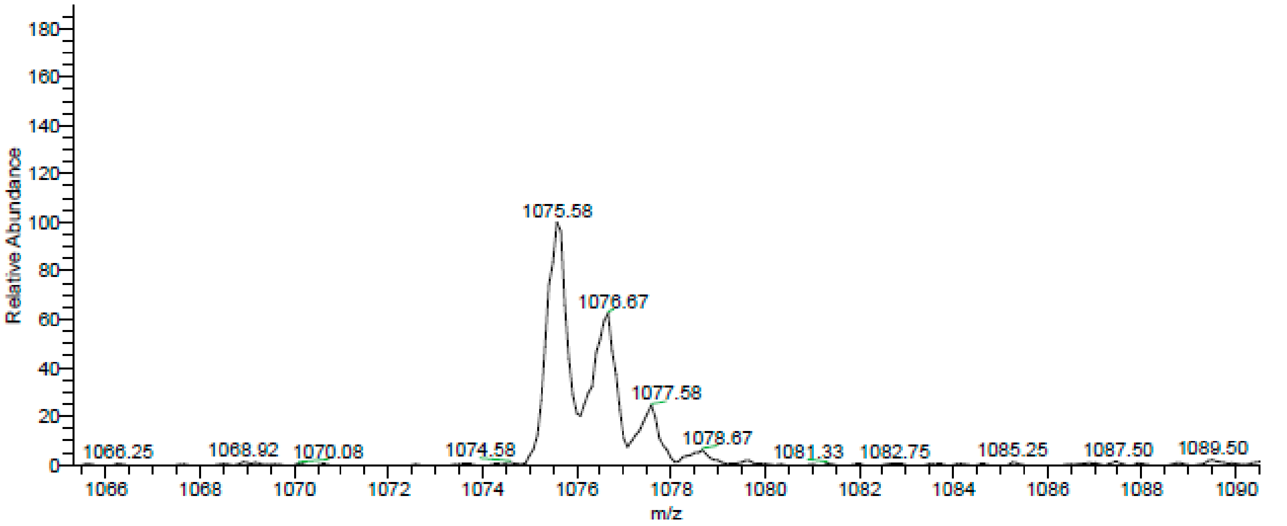
1075.58
1076.67
1077.58
1078.67
1066.25 1068.92 1070.08 1074.58 1081.33 1082.75 1085.25 1087.50 1089.50
Relative Abundance
m/z

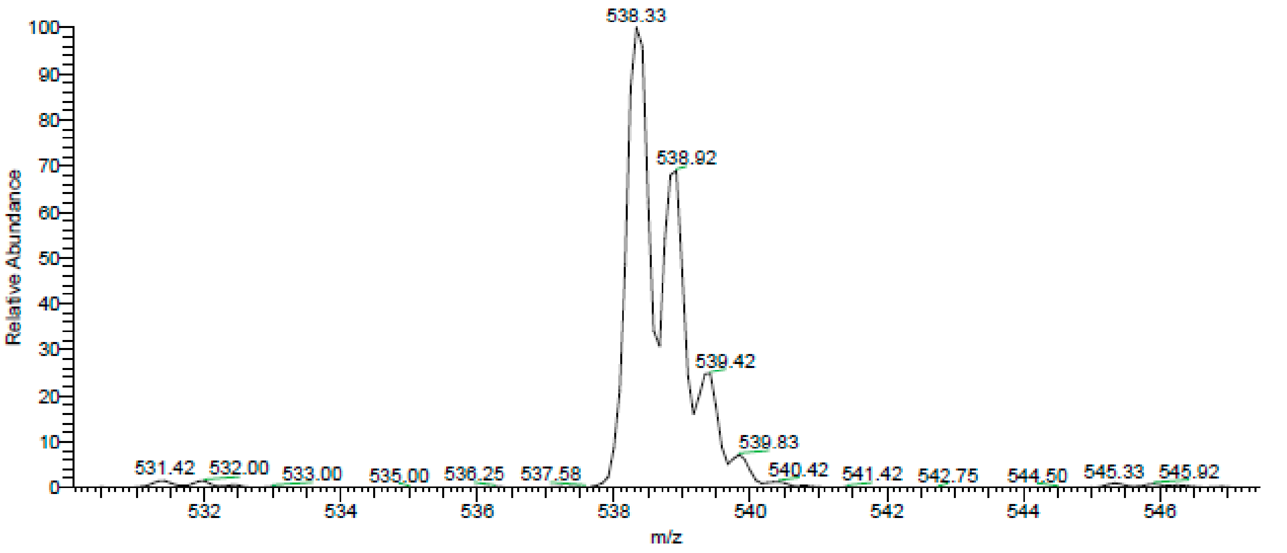
538.33
538.92
539.42
539.83
531.42 532.00 533.00 535.00 536.25 537.58 540.42 541.42 542.75 544.50 545.33 545.92
Relative Abundance
m/z

NDI-8

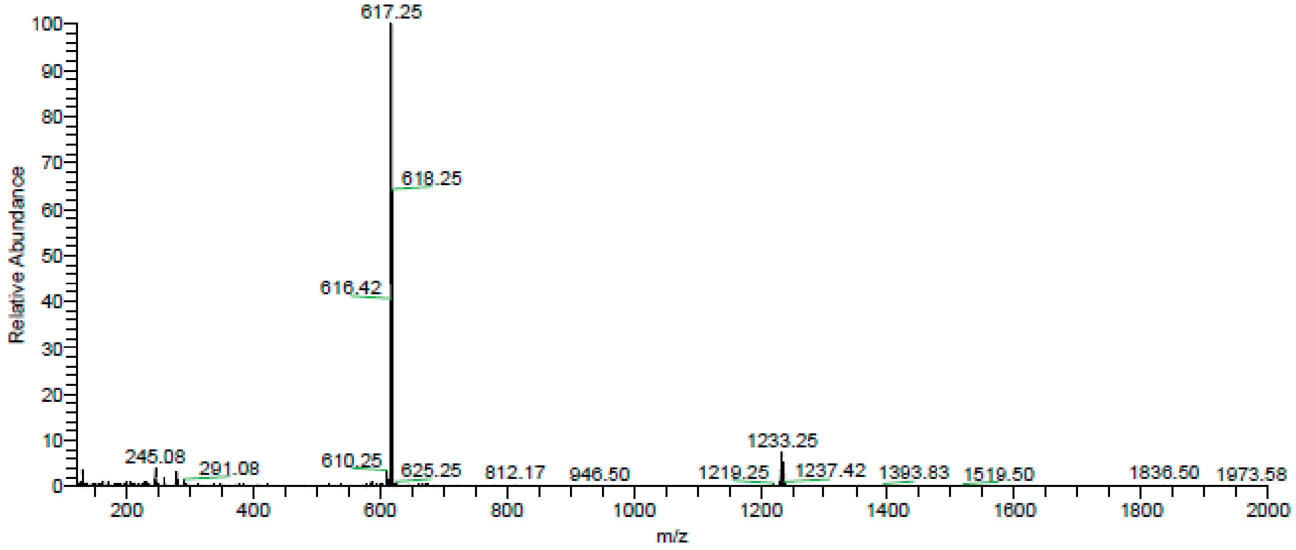

617.25
618.25
616.42
245.08
291.08
610.25
625.25
812.17
946.50
1219.25
1233.25
1237.42
1393.83
1519.50
1836.50
1973.58
Relative Abundance
m/z

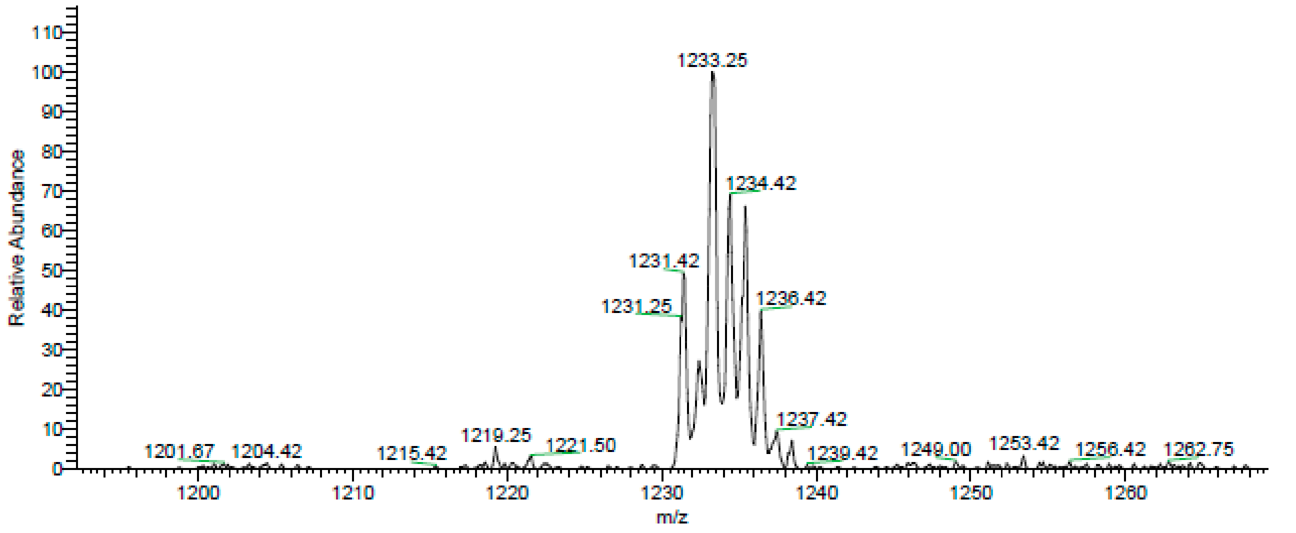

1233.25
1234.42
1231.42
1231.25
1236.42
1237.42
1219.25
1221.50
1201.67
1204.42
1215.42
1239.42
1249.00
1253.42
1256.42
1262.75
Relative Abundance
m/z

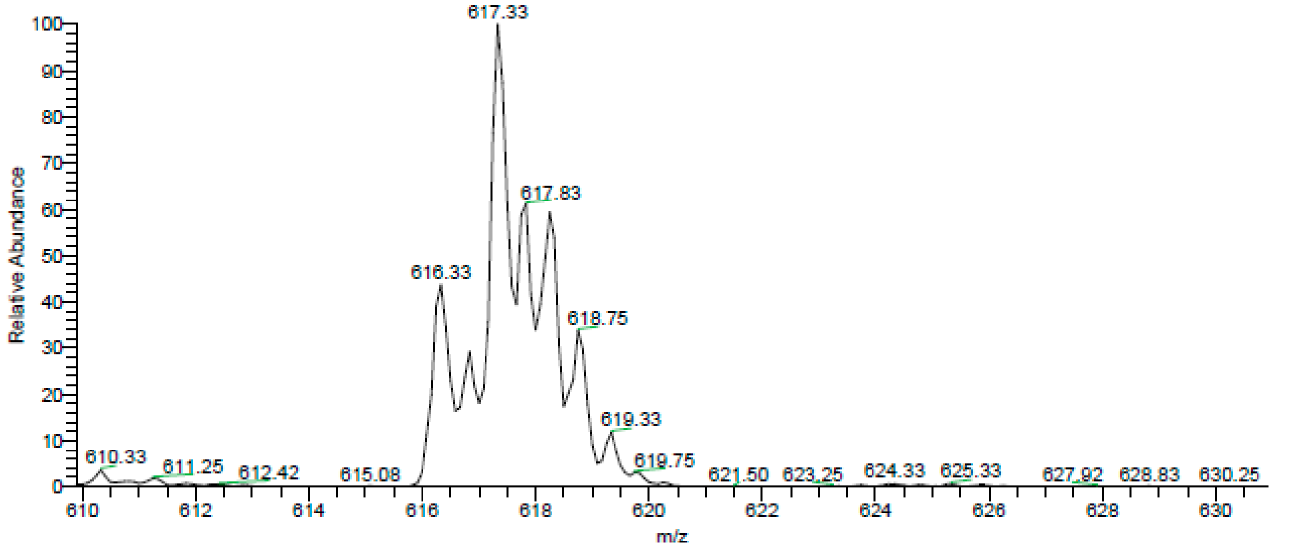

617.33
617.83
616.33
618.75
619.33
619.75
610.33
611.25
612.42
615.08
621.50
623.25
624.33
625.33
627.92
628.83
630.25
Relative Abundance
m/z

NDI-9

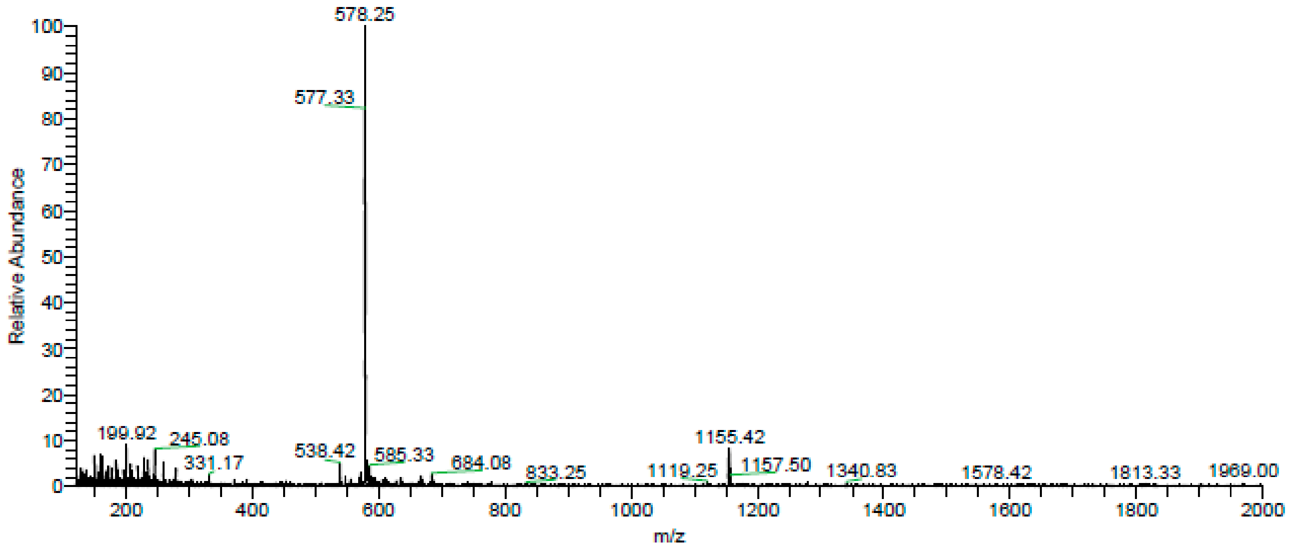
578.25
577.33
199.92
245.08
331.17
538.42
585.33
684.08
833.25
1119.25
1155.42
1157.50
1340.83
1578.42
1813.33
1969.00
Relative Abundance
m/z

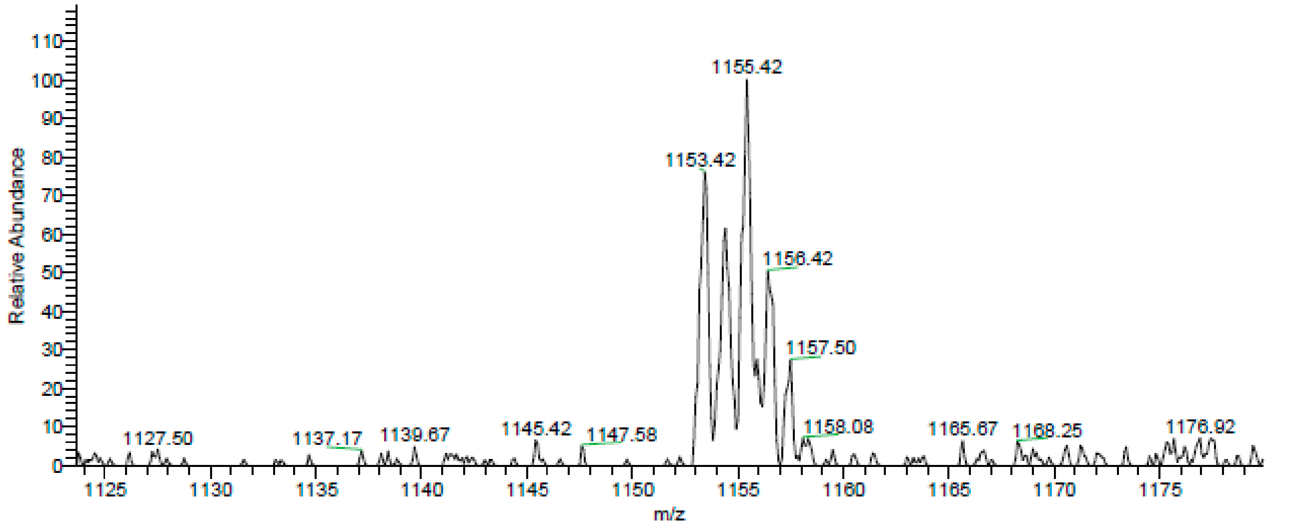
1155.42
1153.42
1156.42
1157.50
1127.50
1137.17
1139.67
1145.42
1147.58
1158.08
1165.67
1168.25
1176.92
Relative Abundance
m/z

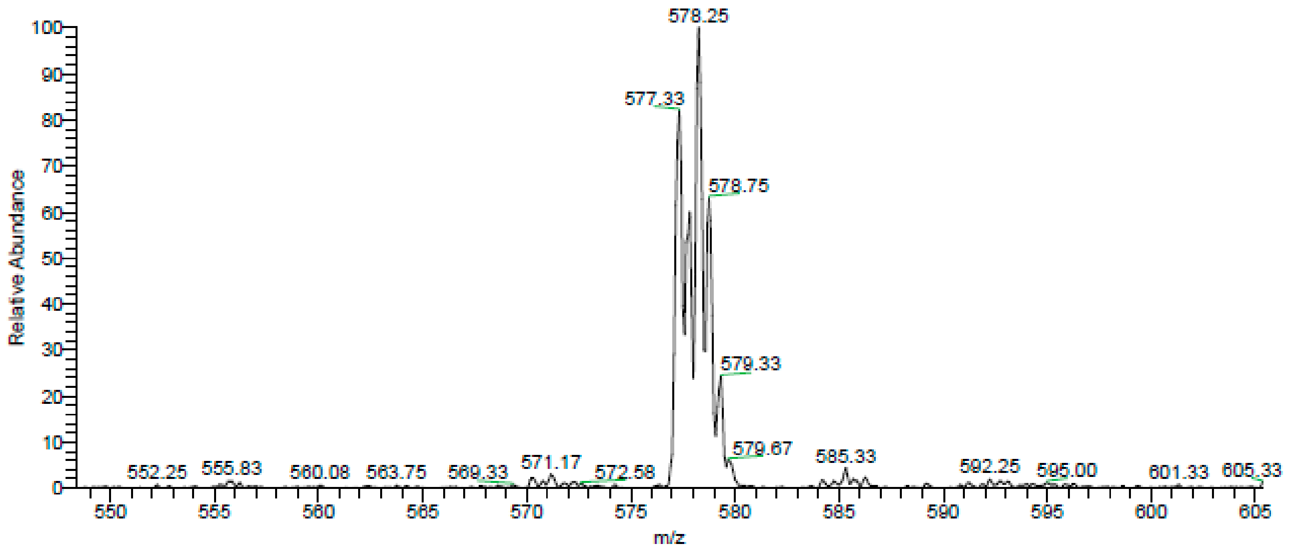
578.25
577.33
578.75
579.33
579.67
552.25
555.83
560.08
563.75
569.33
571.17
572.58
585.33
592.25
595.00
601.33
605.33
Relative Abundance
m/z

NDI-10

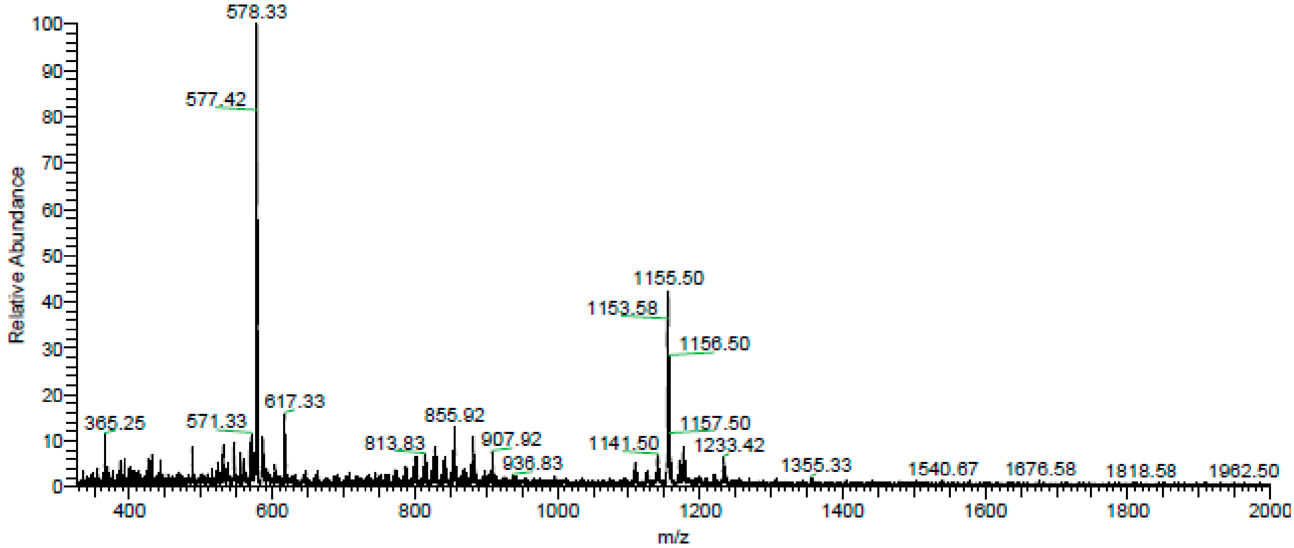
578.33
577.42
1155.50
1153.58
1156.50
617.33
365.25
571.33
855.92
813.83
907.92
936.83
1141.50
1157.50
1233.42
1355.33
1540.67
1676.58
1818.58
1962.50
Relative Abundance
m/z

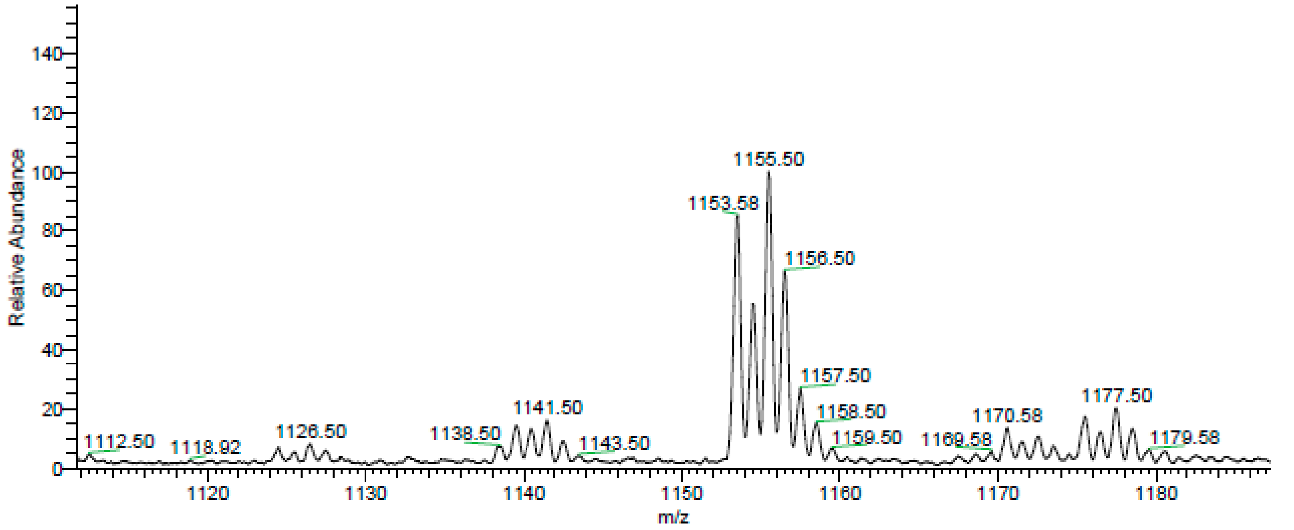
1155.50
1153.58
1156.50
1157.50
1158.50
1159.50
1141.50
1138.50
1143.50
1126.50
1112.50
1118.92
1170.58
1169.58
1177.50
1179.58
Relative Abundance
m/z

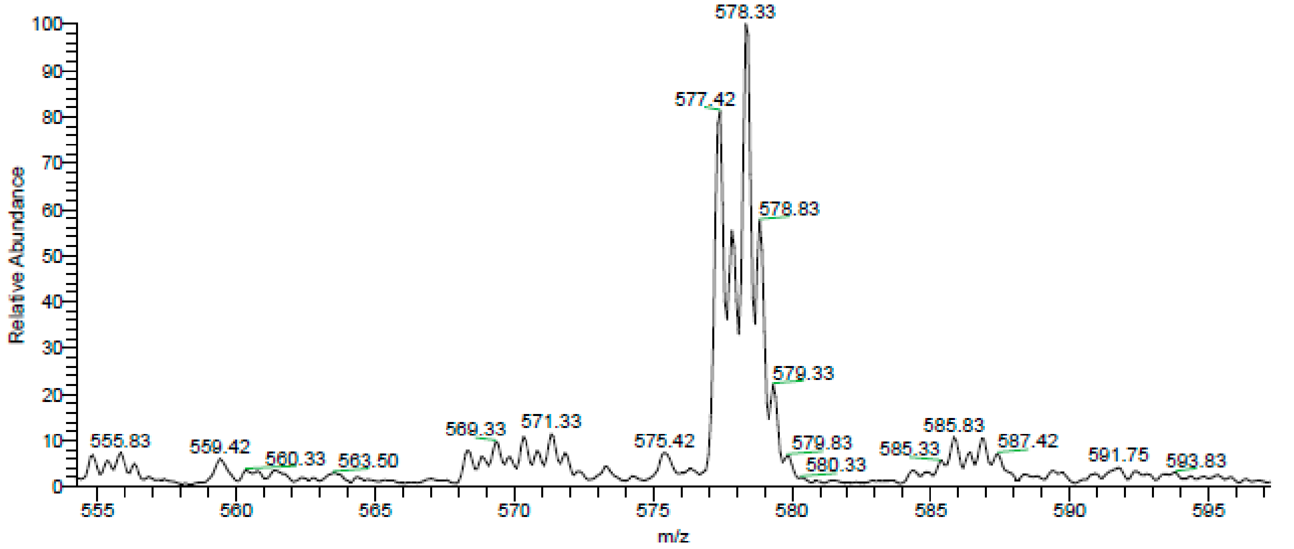
578.33
577.42
578.83
579.33
579.83
580.33
555.83
559.42
560.33
563.50
569.33
571.33
575.42
585.33
585.83
587.42
591.75
593.83
Relative Abundance
m/z

NDI-11

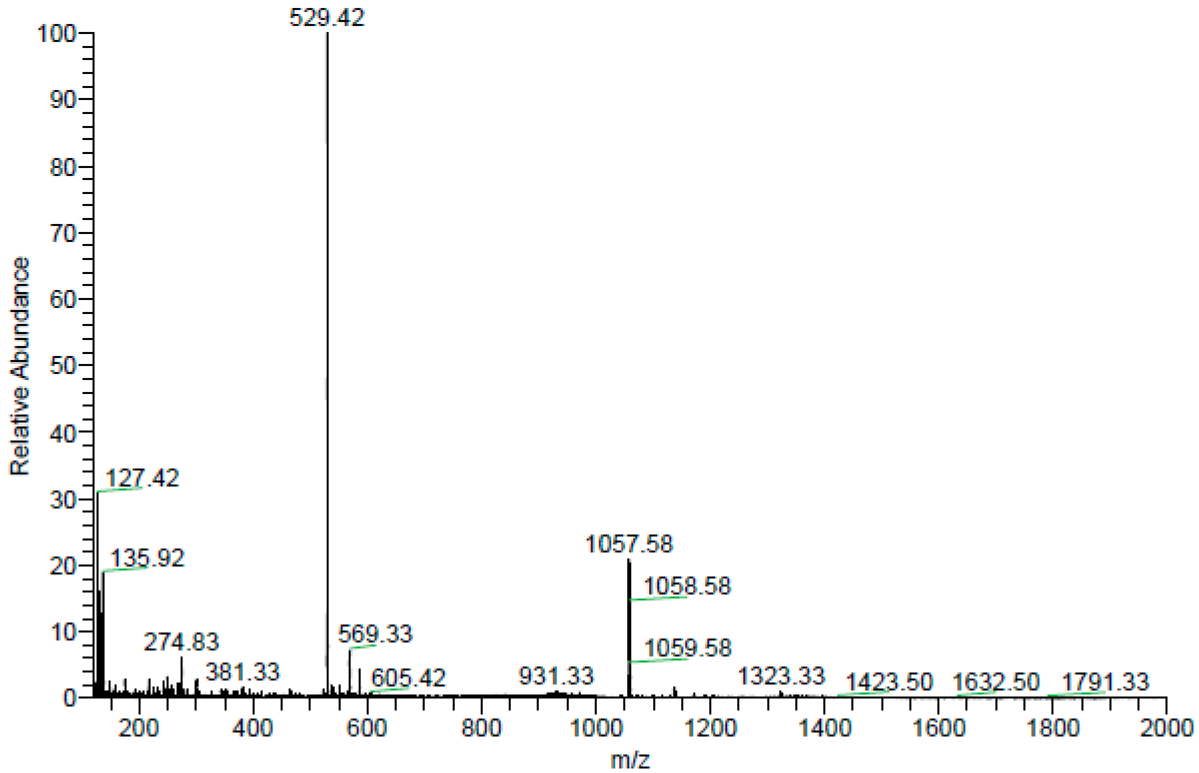
529.42
127.42
135.92
274.83
381.33
569.33
605.42
931.33
1057.58
1058.58
1059.58
1323.33
1423.50
1632.50
1791.33
Relative Abundance
m/z

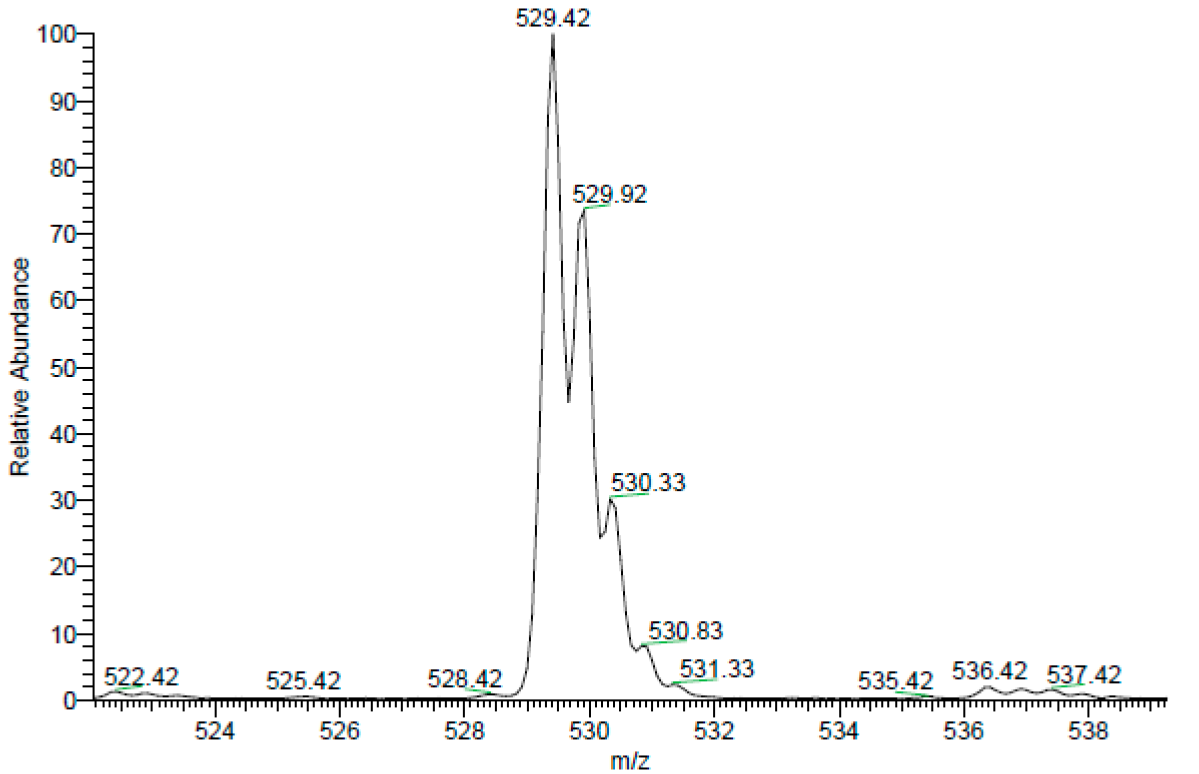
529.42
529.92
530.33
530.83
531.33
522.42
525.42
528.42
535.42
536.42
537.42
Relative Abundance
m/z

NDI-12

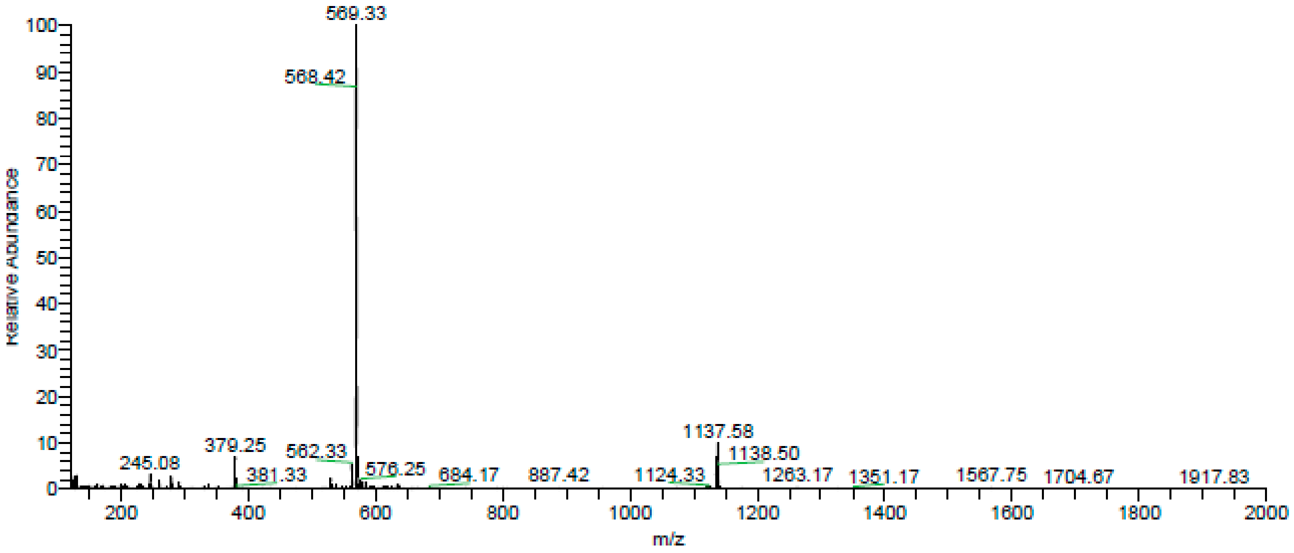
569.33
568.42
379.25
245.08
381.33
562.33
576.25
684.17
887.42
1124.33
1137.58
1138.50
1263.17
1351.17
1567.75
1704.67
1917.83
Relative Abundance
m/z

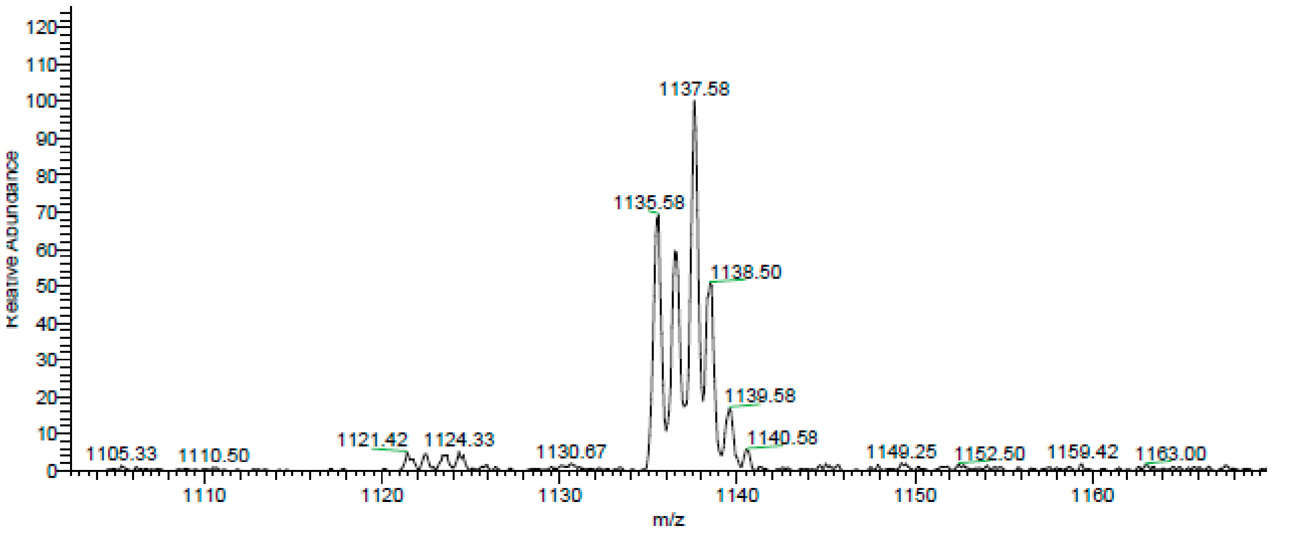
1137.58
1135.58
1138.50
1139.58
1140.58
1105.33
1110.50
1121.42
1124.33
1130.67
1149.25
1152.50
1159.42
1163.00
Relative Abundance
m/z

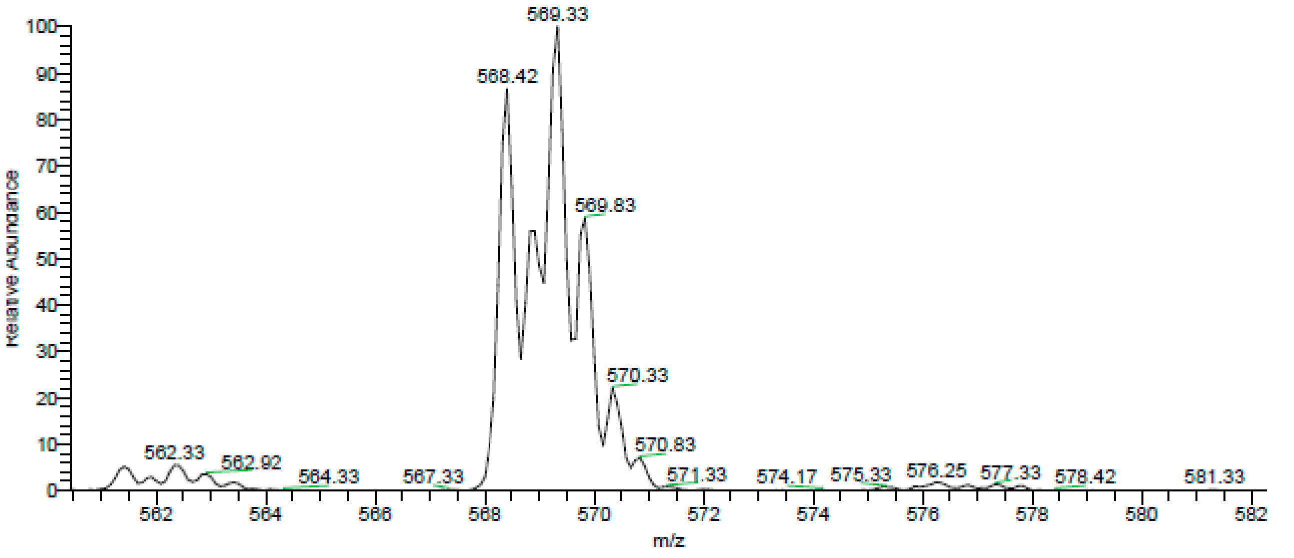
569.33
568.42
569.83
570.33
570.83
562.33
562.92
564.33
567.33
571.33
574.17
575.33
576.25
577.33
578.42
581.33
Relative Abundance
m/z

NDI-6

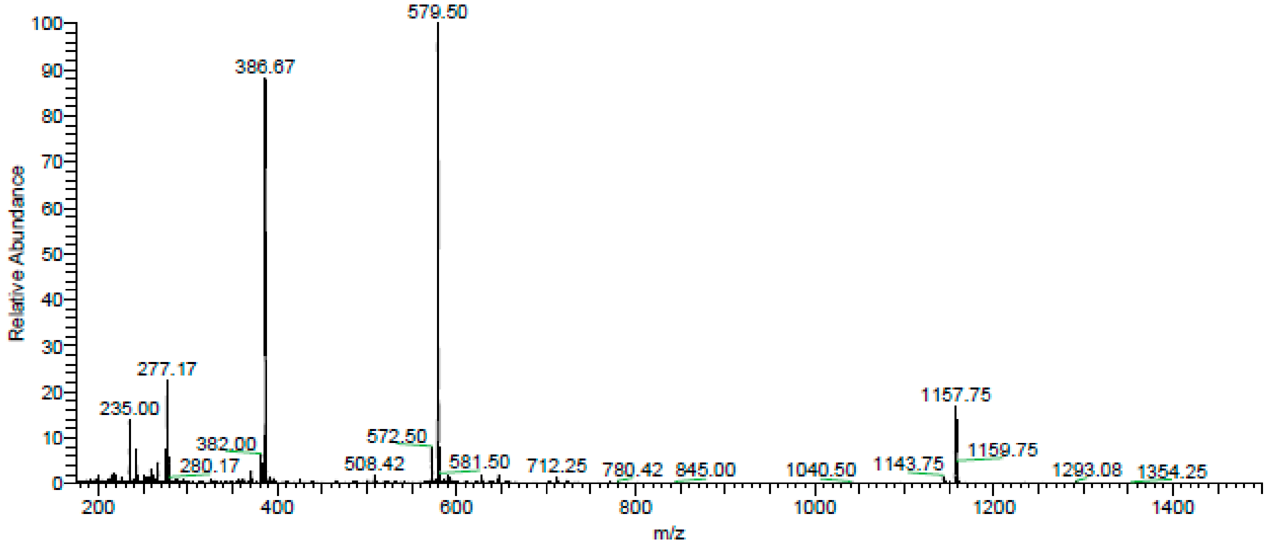
579.50
386.67
277.17
235.00
382.00
280.17
572.50
508.42
581.50
712.25
780.42
845.00
1040.50
1143.75
1157.75
1159.75
1293.08
1354.25
Relative Abundance
m/z

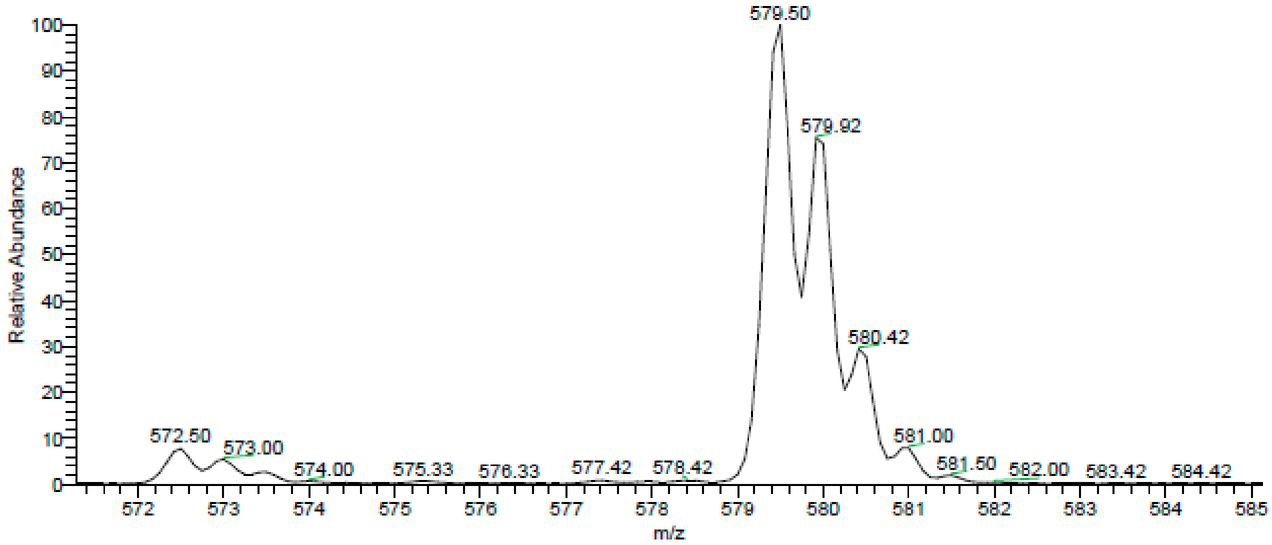
579.50
579.92
580.42
581.00
581.50
582.00
583.42
584.42
572.50
573.00
574.00
575.33
576.33
577.42
578.42
Relative Abundance
m/z

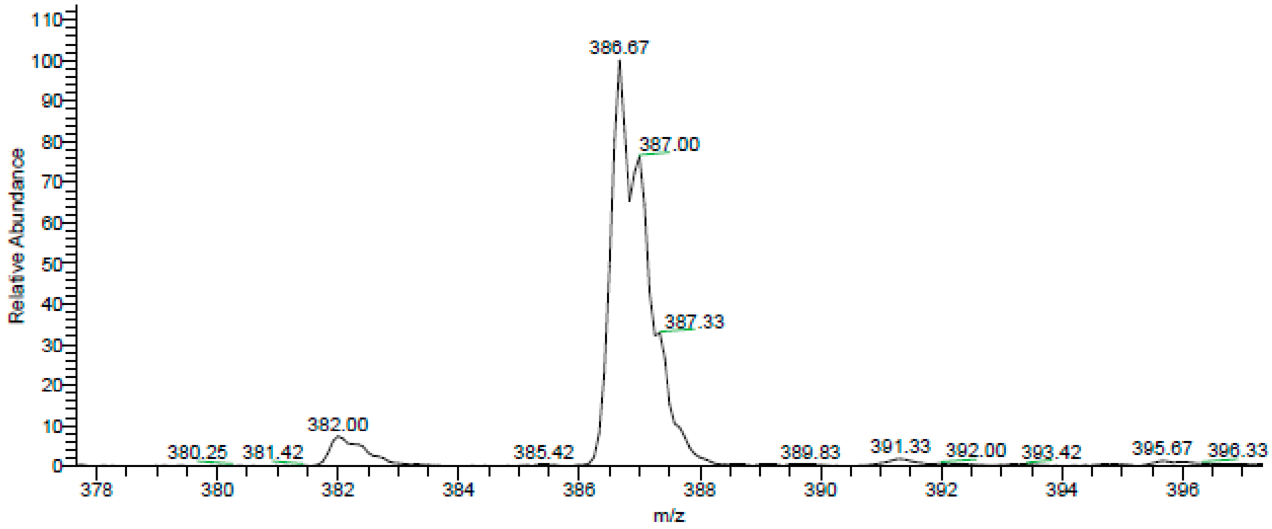
386.67
387.00
387.33
382.00
380.25
381.42
385.42
389.83
391.33
392.00
393.42
395.67
396.33
Relative Abundance
m/z

^{1}H- and ^{13}C-NMR characterization for the here synthesized NDIs

NDI-4•3CF$_3$COOH. ^{1}H-NMR (300 MHz, CD$_3$OD)

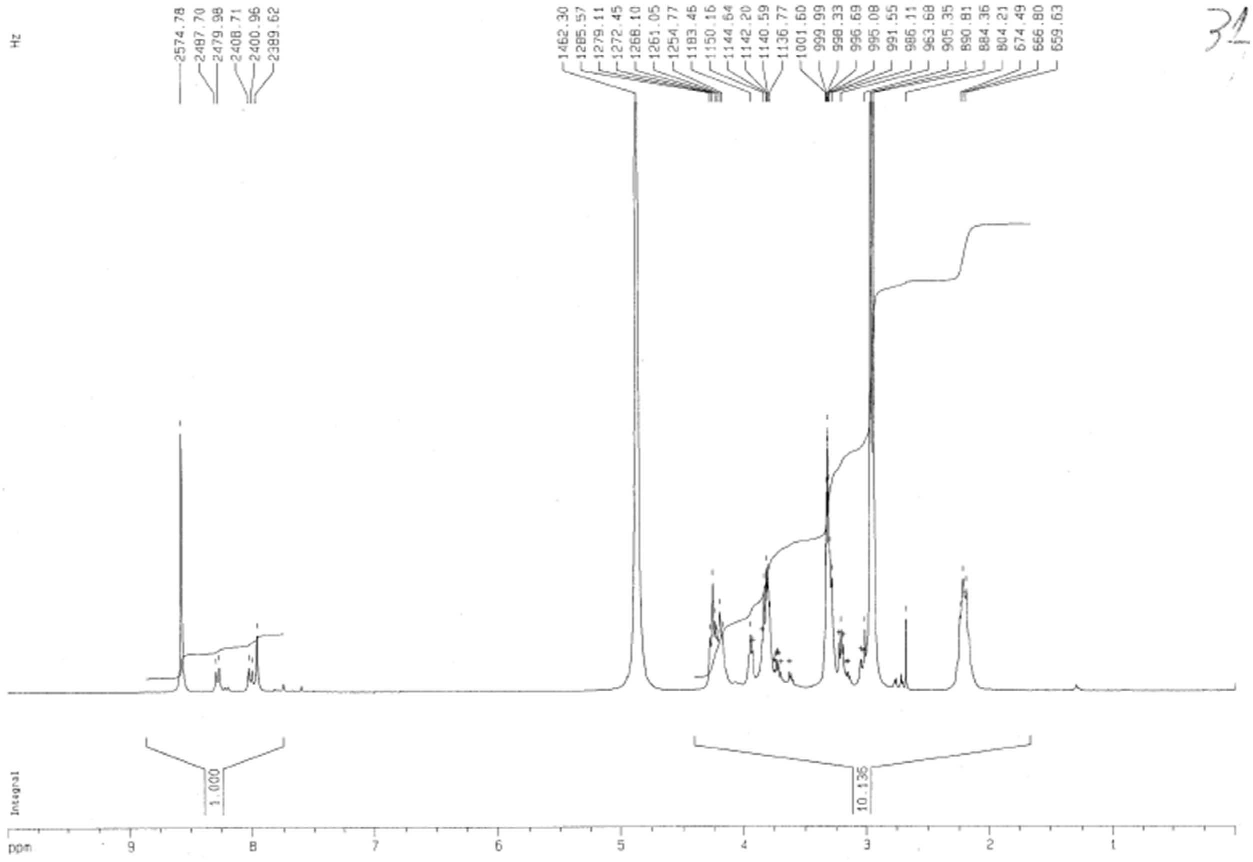

NDI-4•3CF$_3$COOH. ^{13}C-NMR (75 MHz, CD$_3$OD)

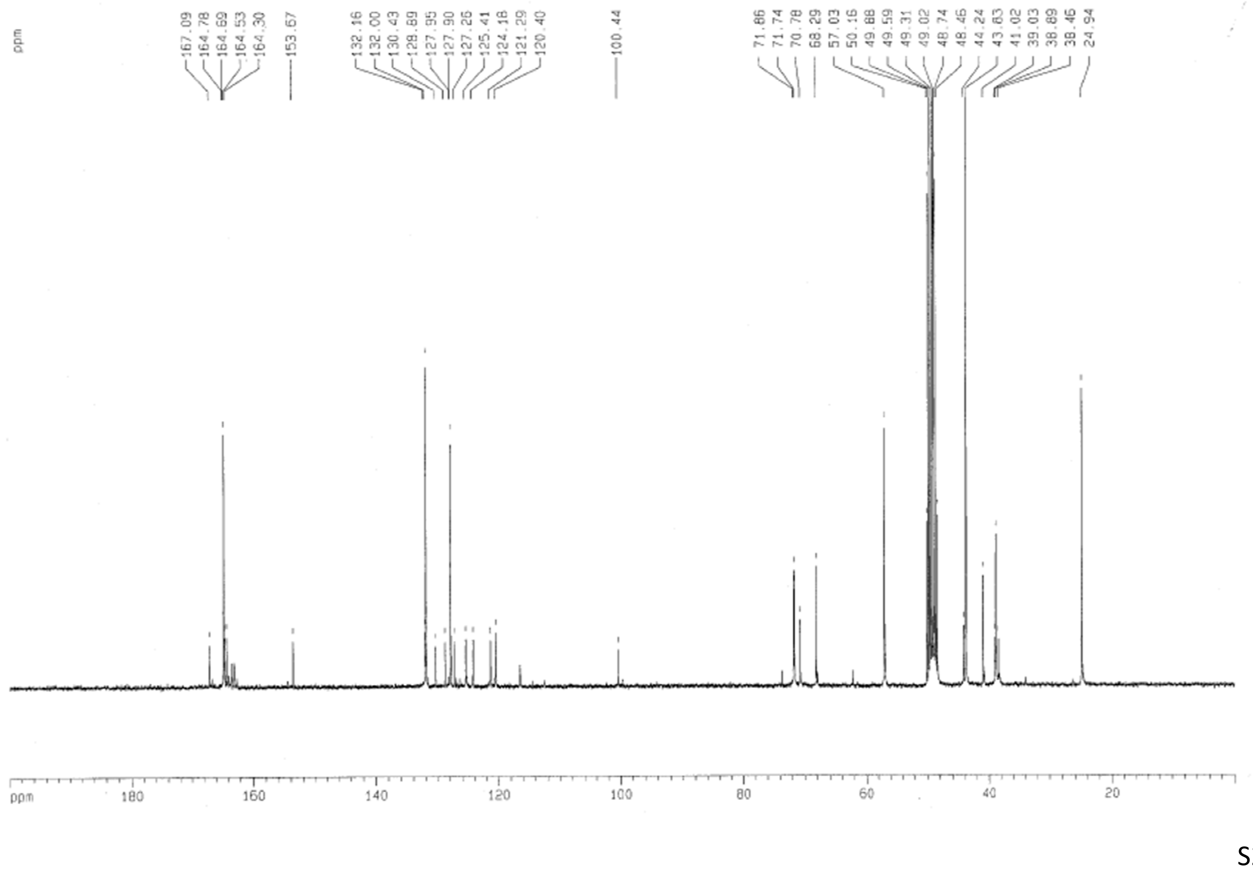

NDI-5•2CF$_3$COOH. ^{1}H-NMR (300 MHz, D$_2$O)

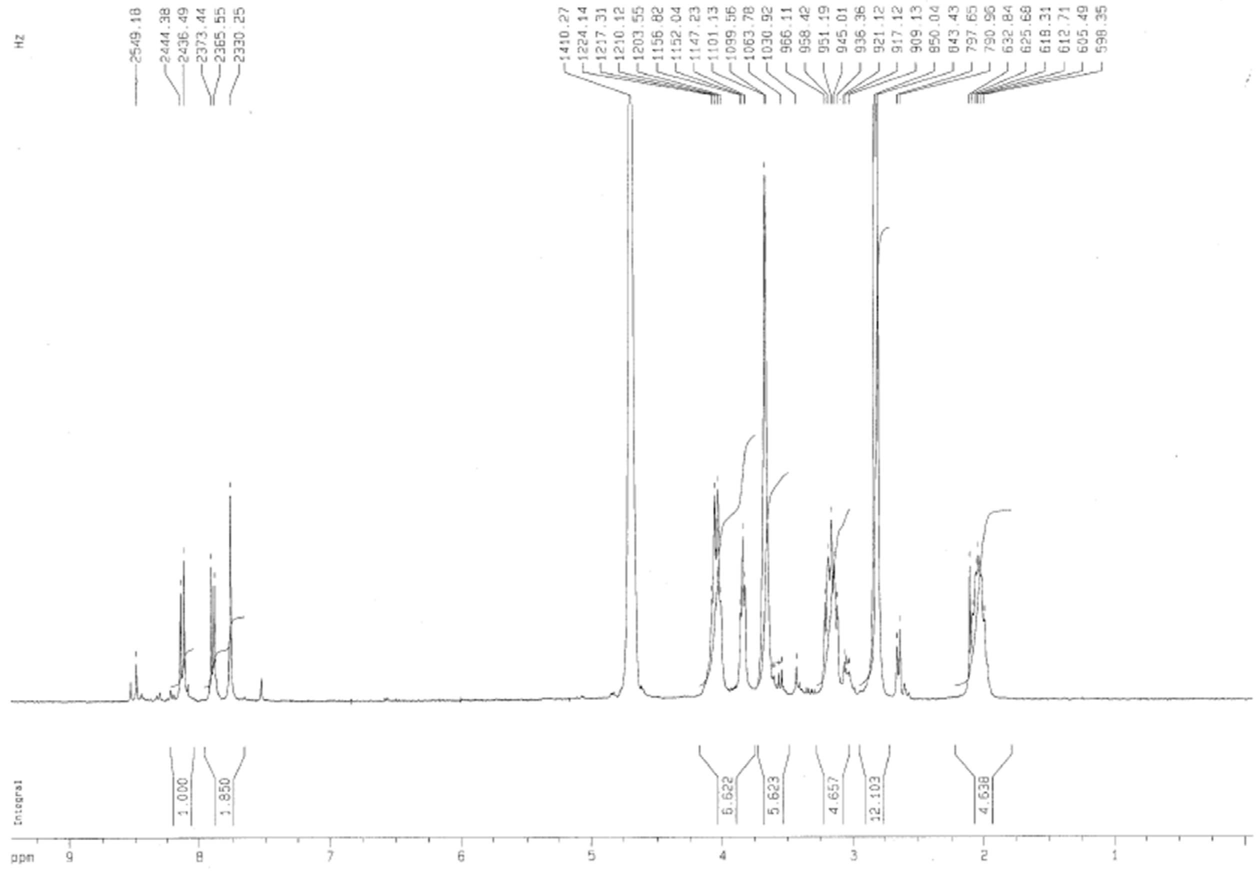

NDI-5•2CF$_3$COOH. ^{13}C-NMR (75 MHz, D$_2$O)

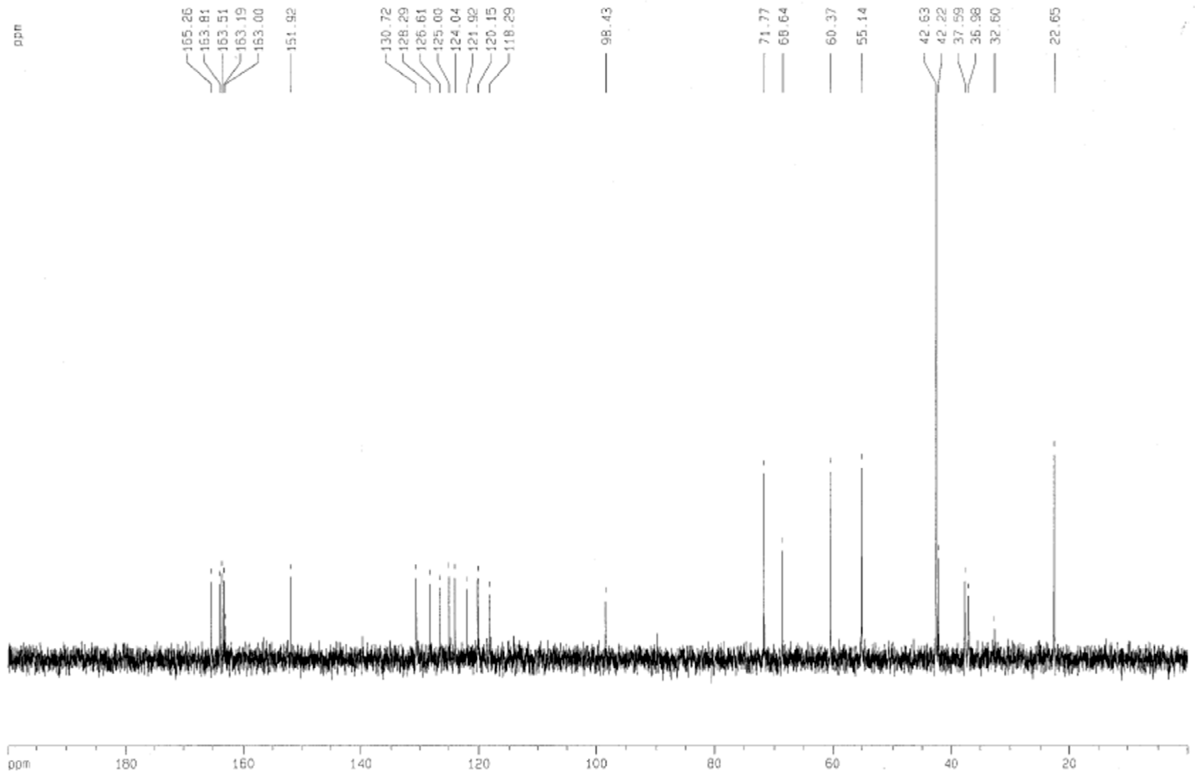

NDI-16•3CF$_3$COOH. ^{1}H-NMR (300 MHz, CD$_3$OD)

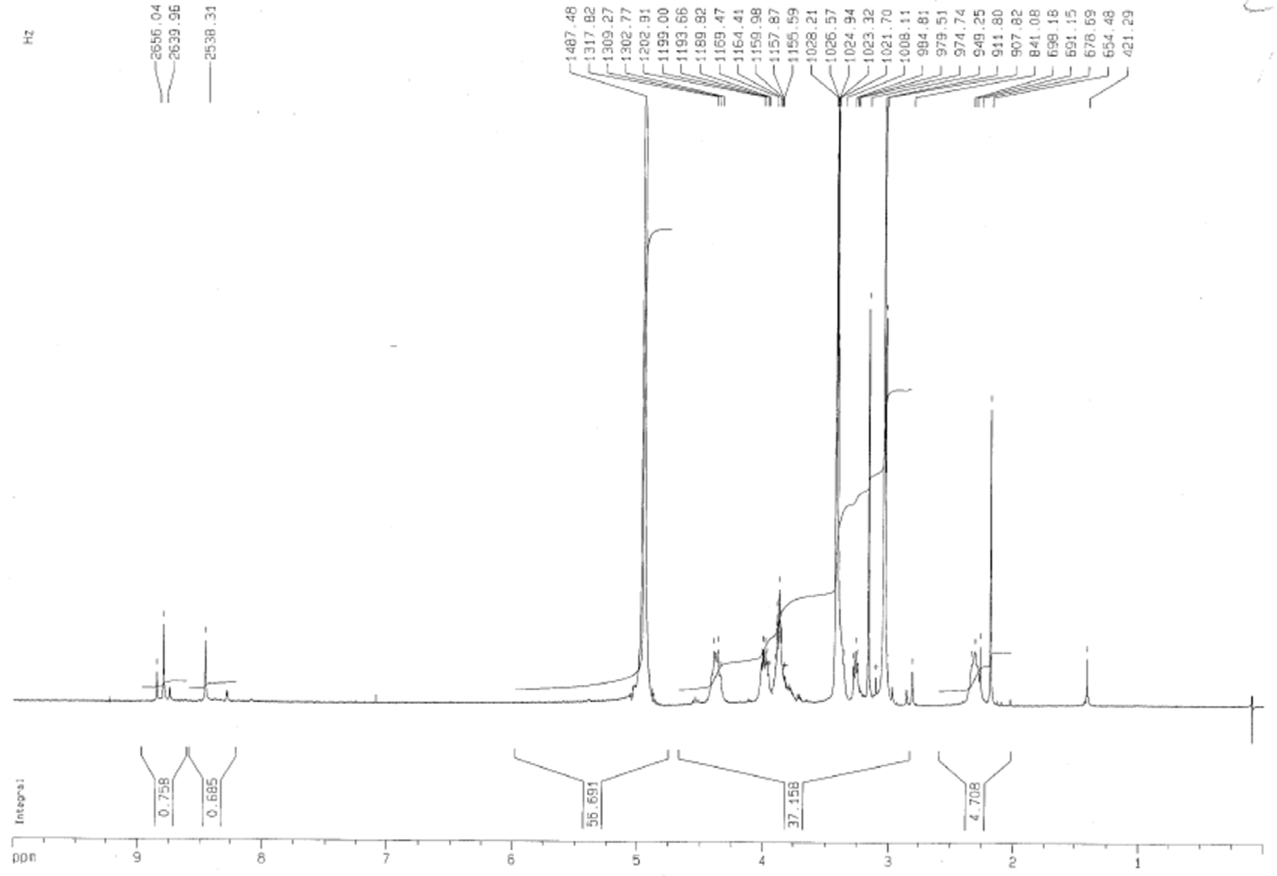

NDI-16•3CF$_3$COOH. ^{13}C-NMR (300 MHz, CD$_3$OD)

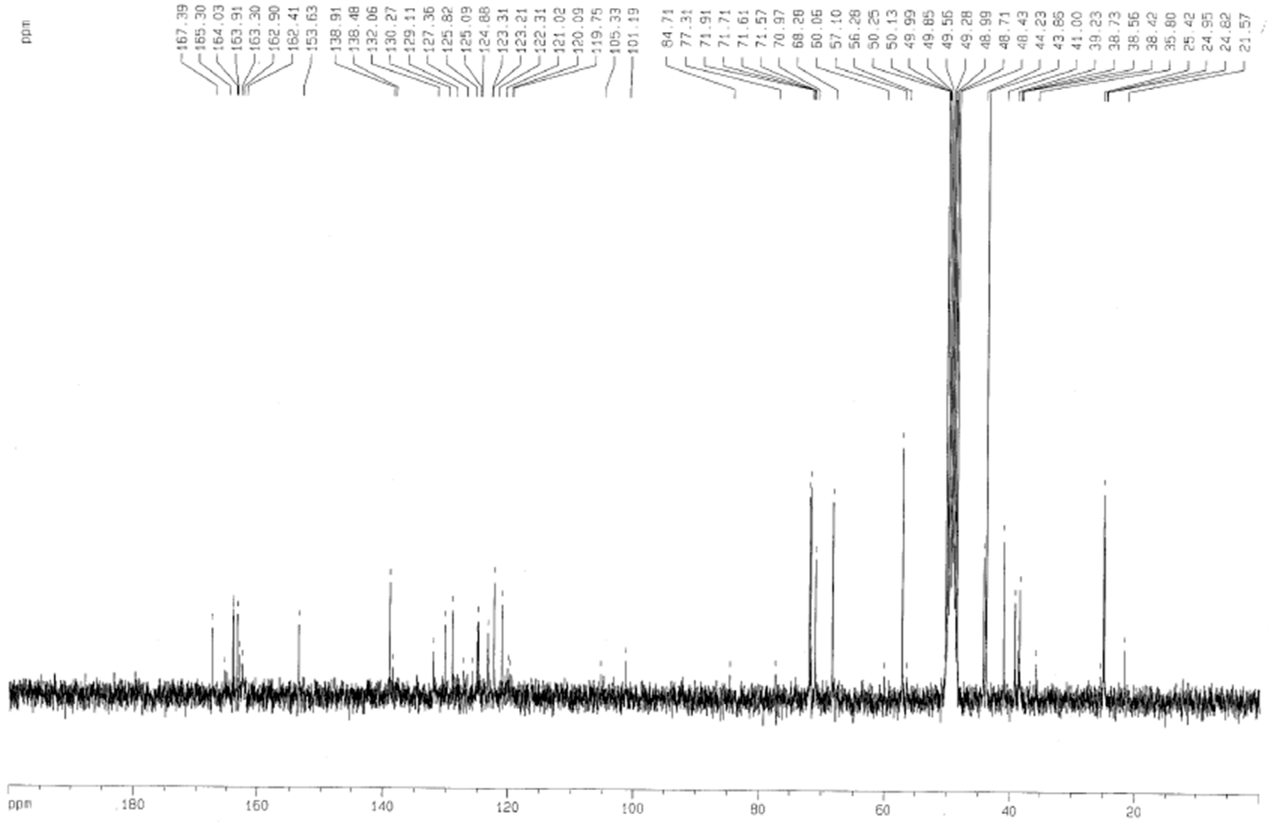

NDI-18. ^{1}H-NMR (300 MHz, DMSO-d$_6$)

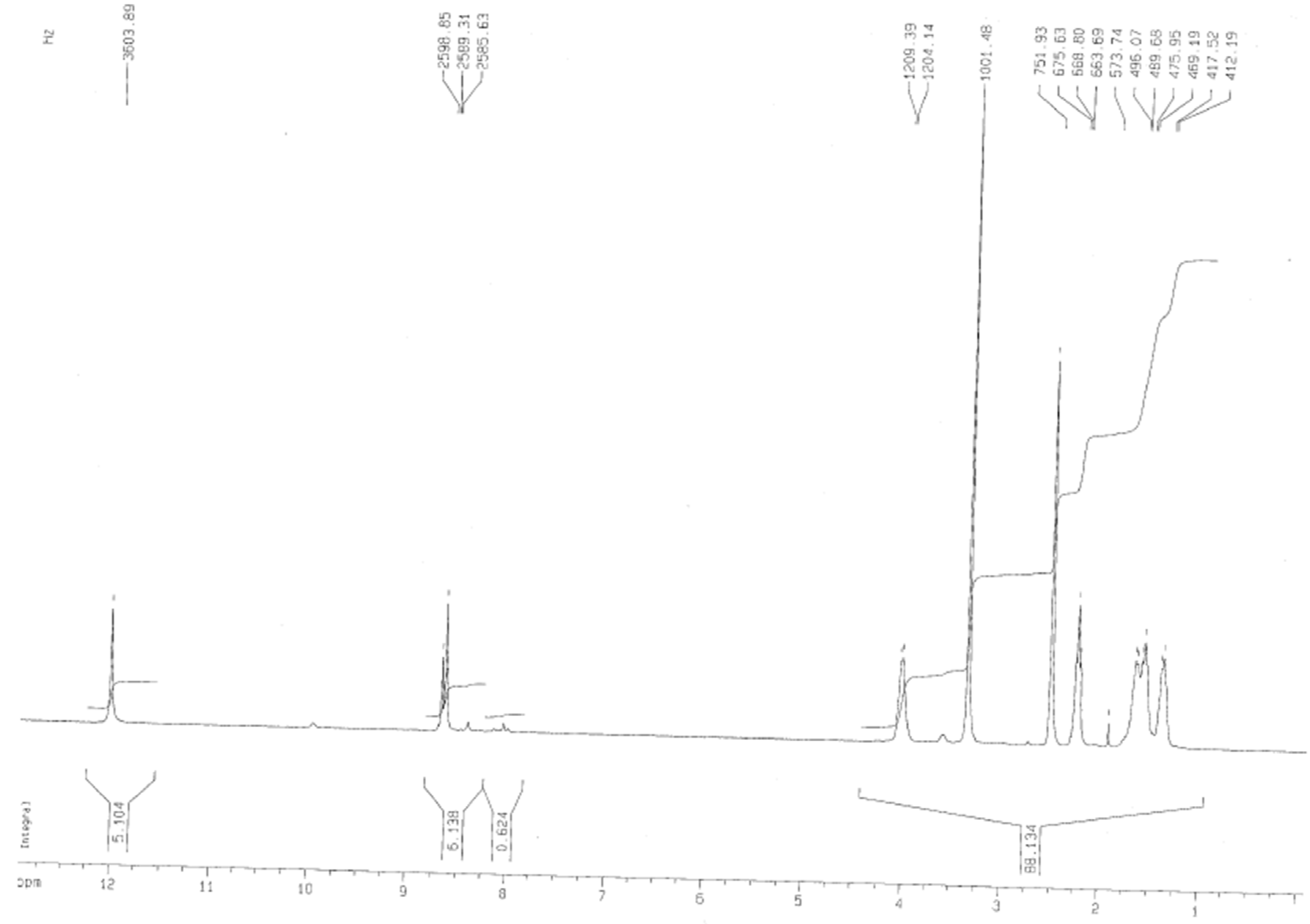

NDI-18. ^{13}C-NMR (75 MHz, DMSO-d$_6$)

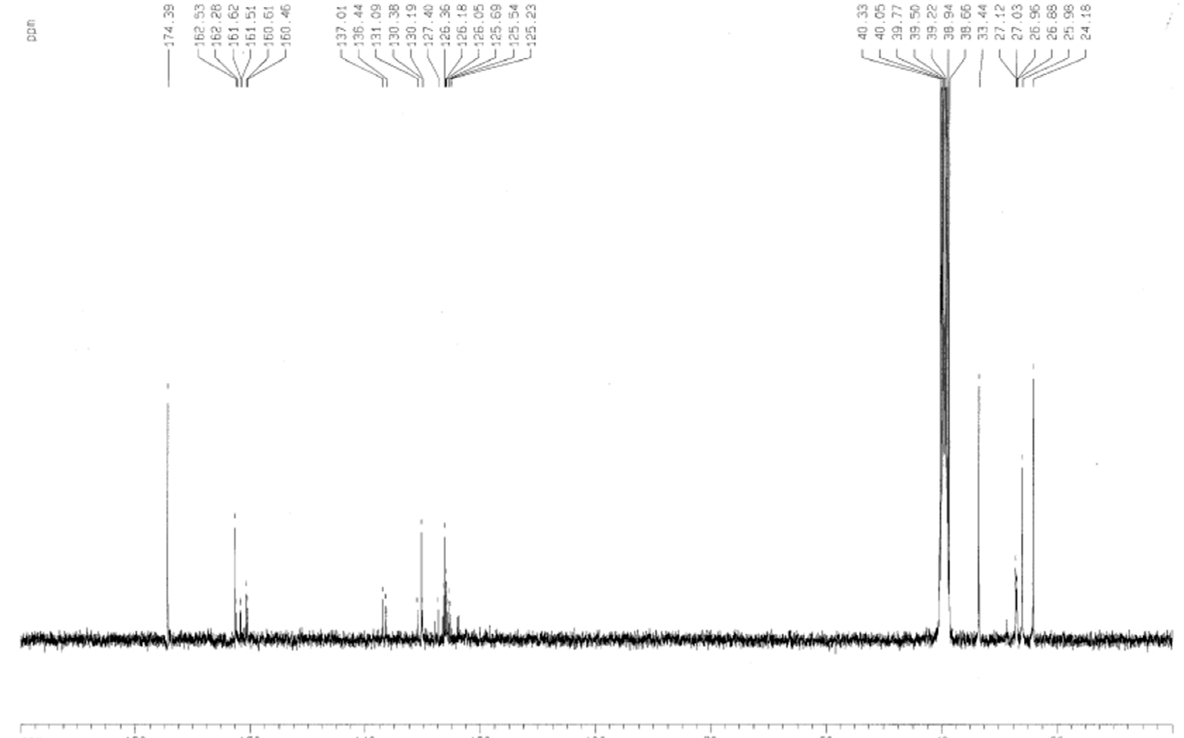

NDI-7•2CF₃COOH. ^{1}H-NMR (300 MHz, CD₃OD)

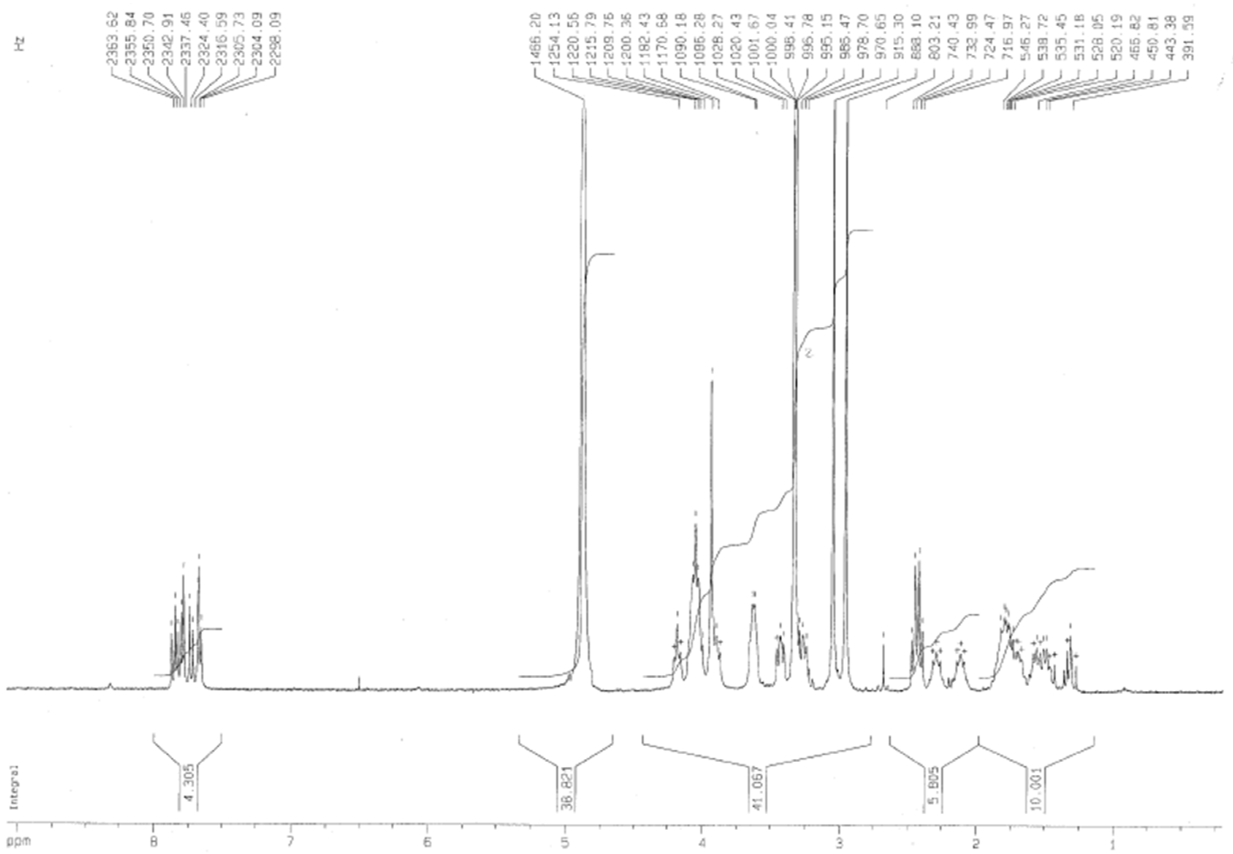

NDI-7•2CF₃COOH. ^{13}C-NMR (75 MHz, CD₃OD)

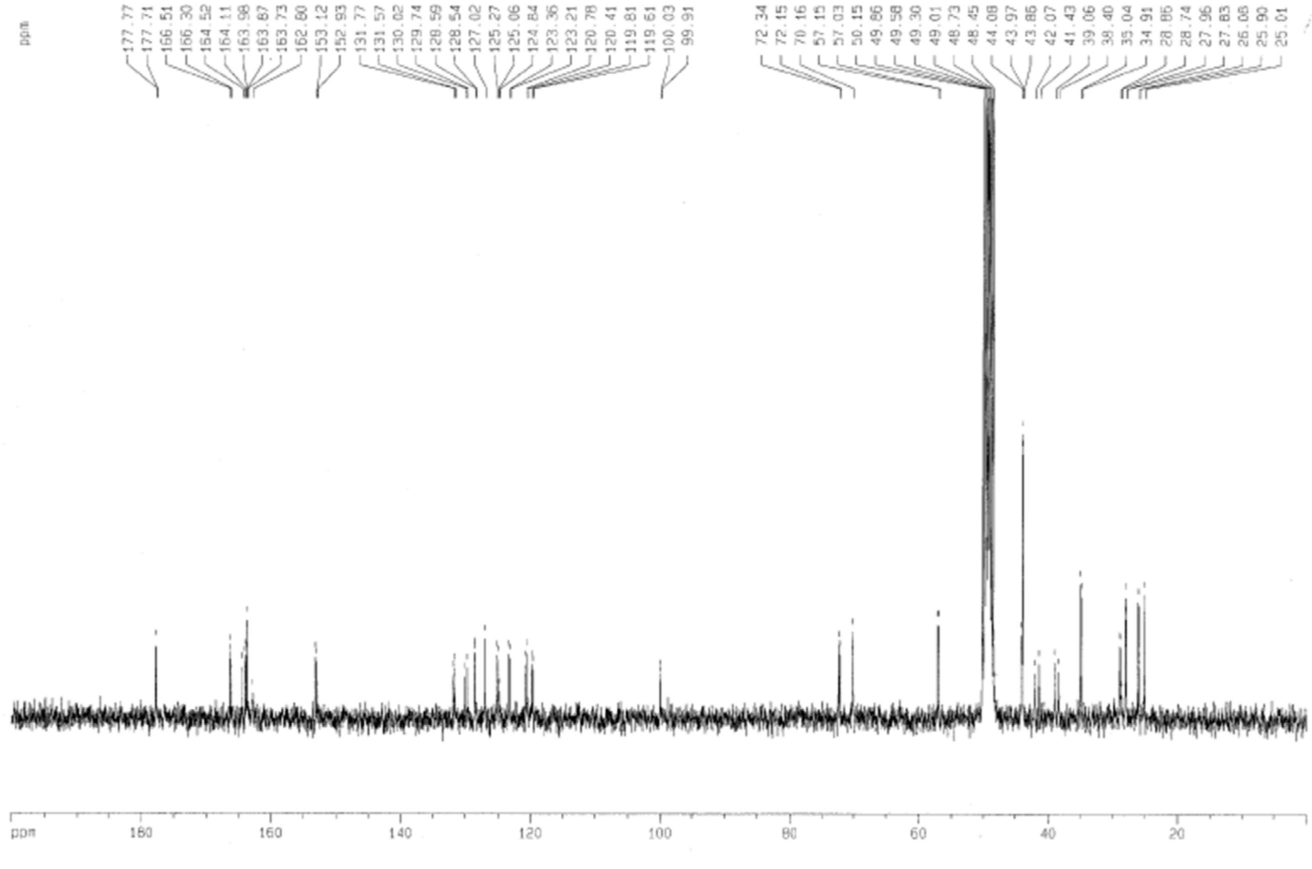

NDI-8•2CF3COOH. ^{1}H-NMR (300 MHz, D_2O)

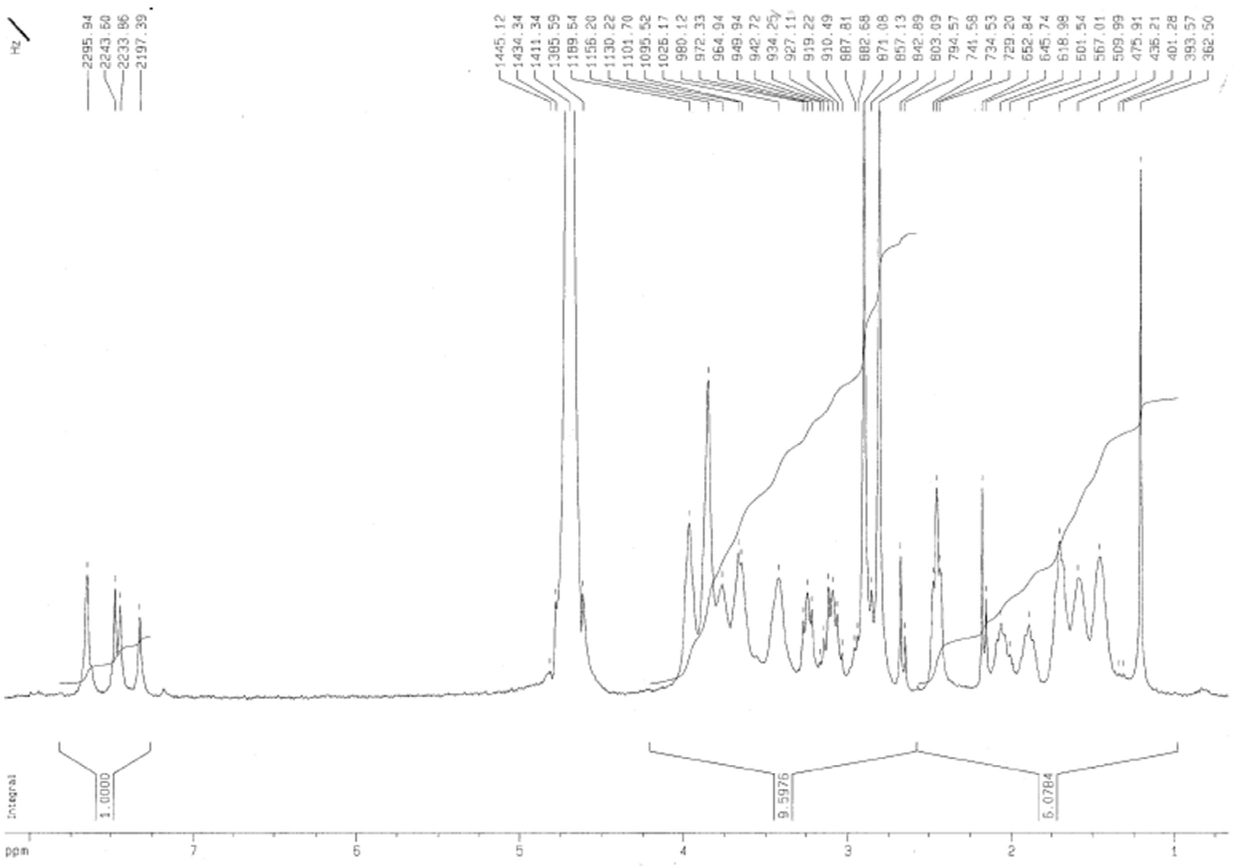

NDI-8•2CF3COOH. ^{13}C-NMR (75 MHz, D_2O)

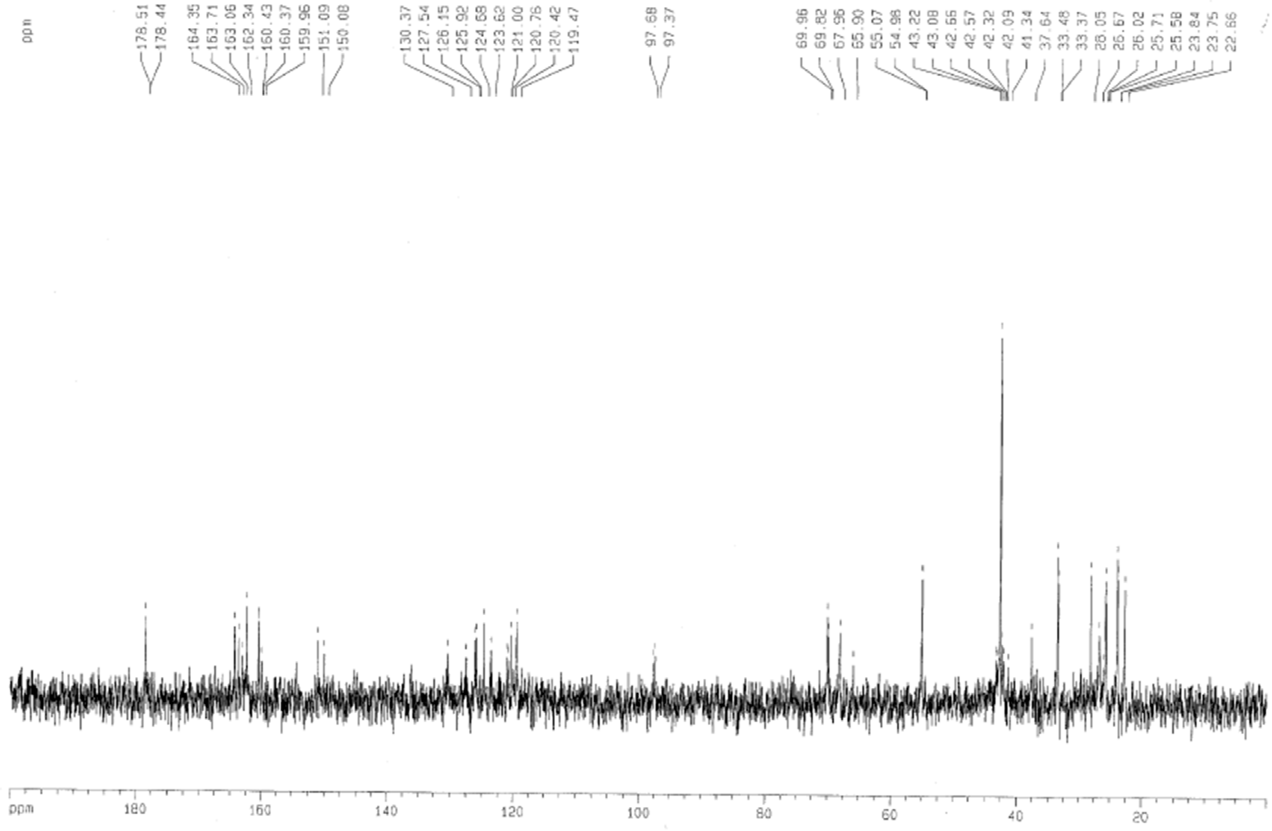

NDI-9•2CF₃COOH. ^{1}H-NMR (300 MHz, D_2O)

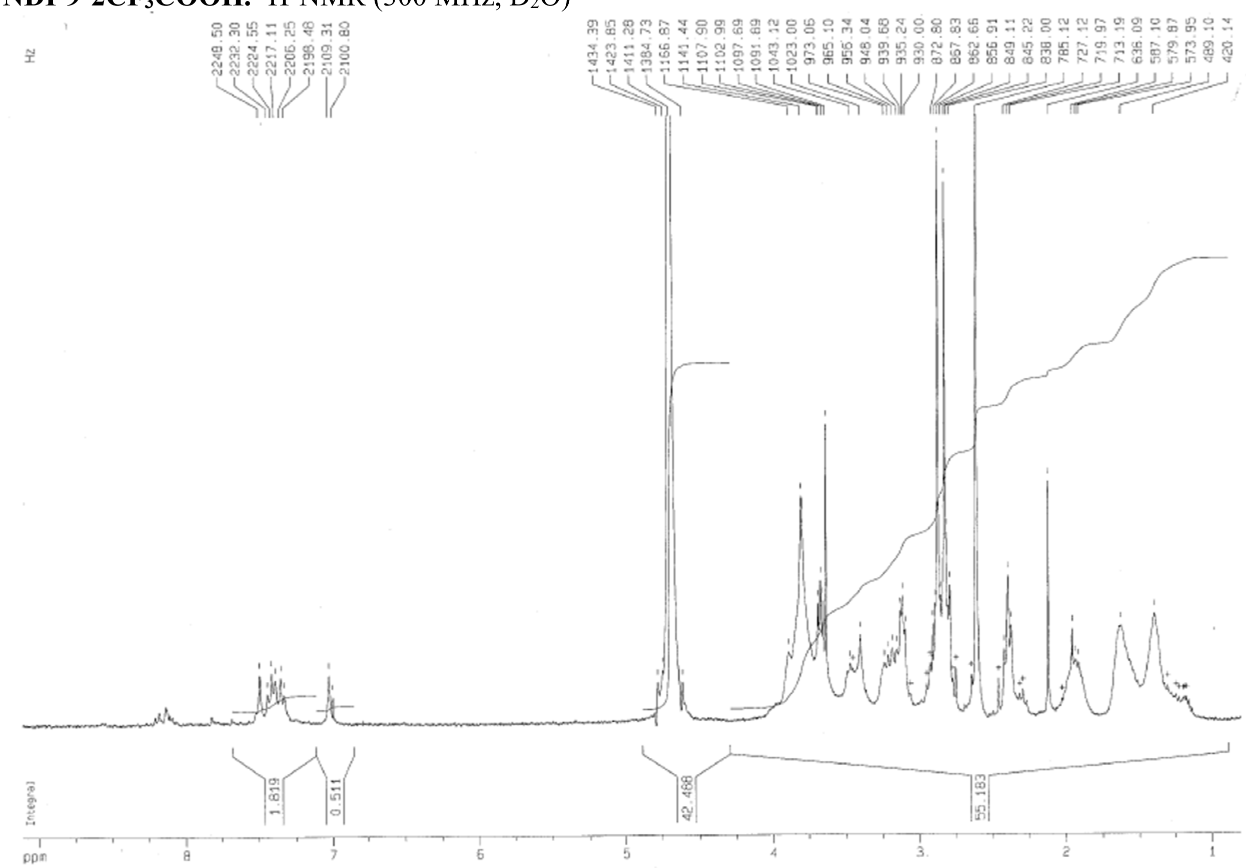

NDI-9•2CF₃COOH. ^{13}C-NMR (300 MHz, D_2O)

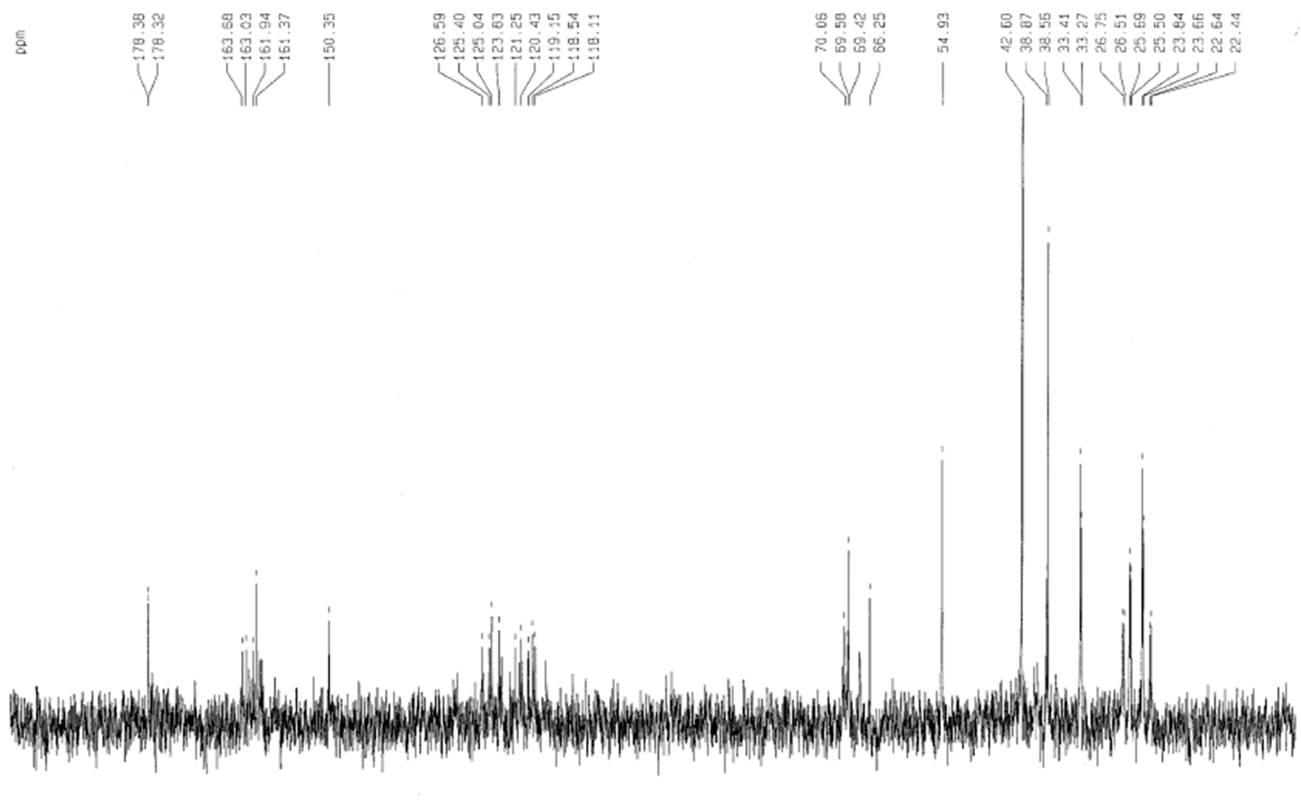

NDI-10•2CF$_3$COOH. ^{1}H-NMR (300 MHz, D_2O)

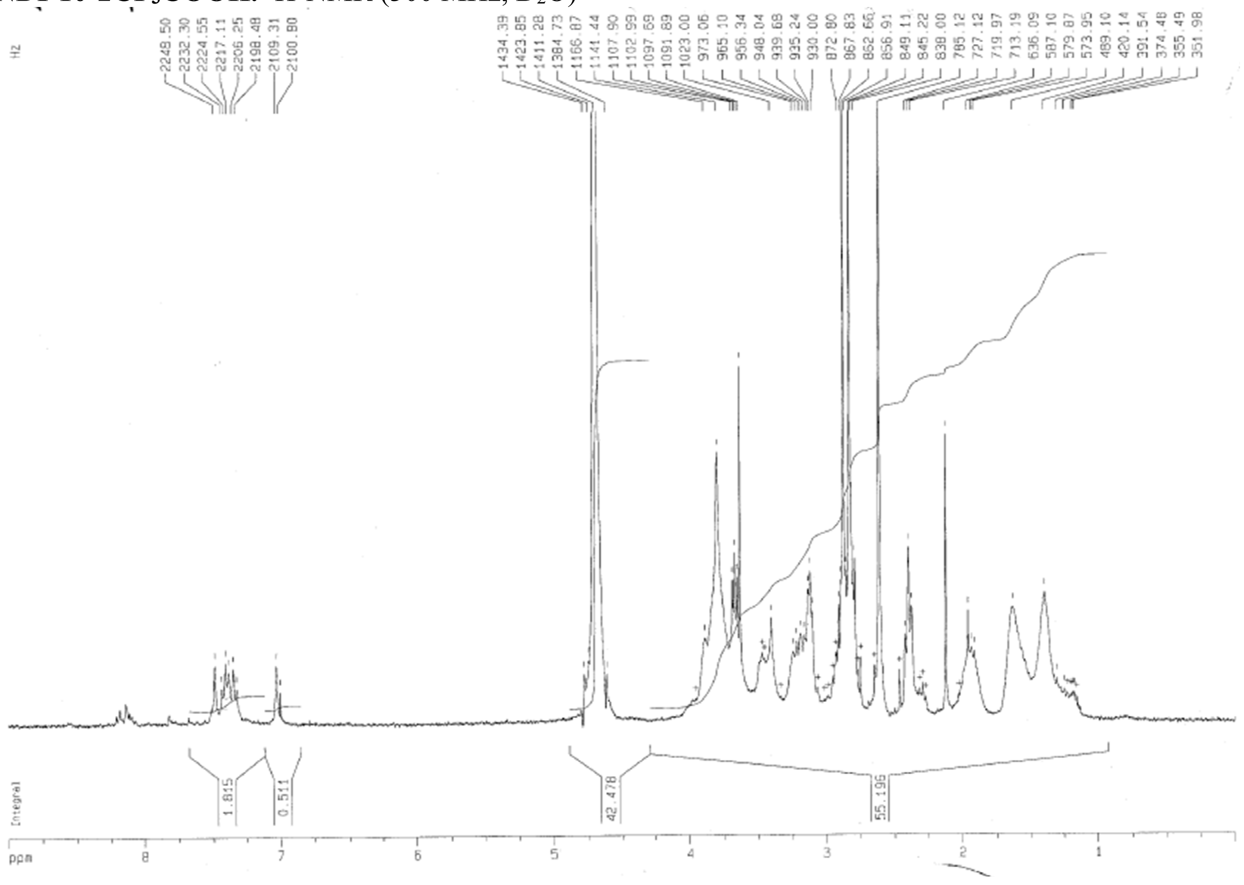

NDI-10•2CF$_3$COOH. ^{13}C-NMR (300 MHz, D_2O)

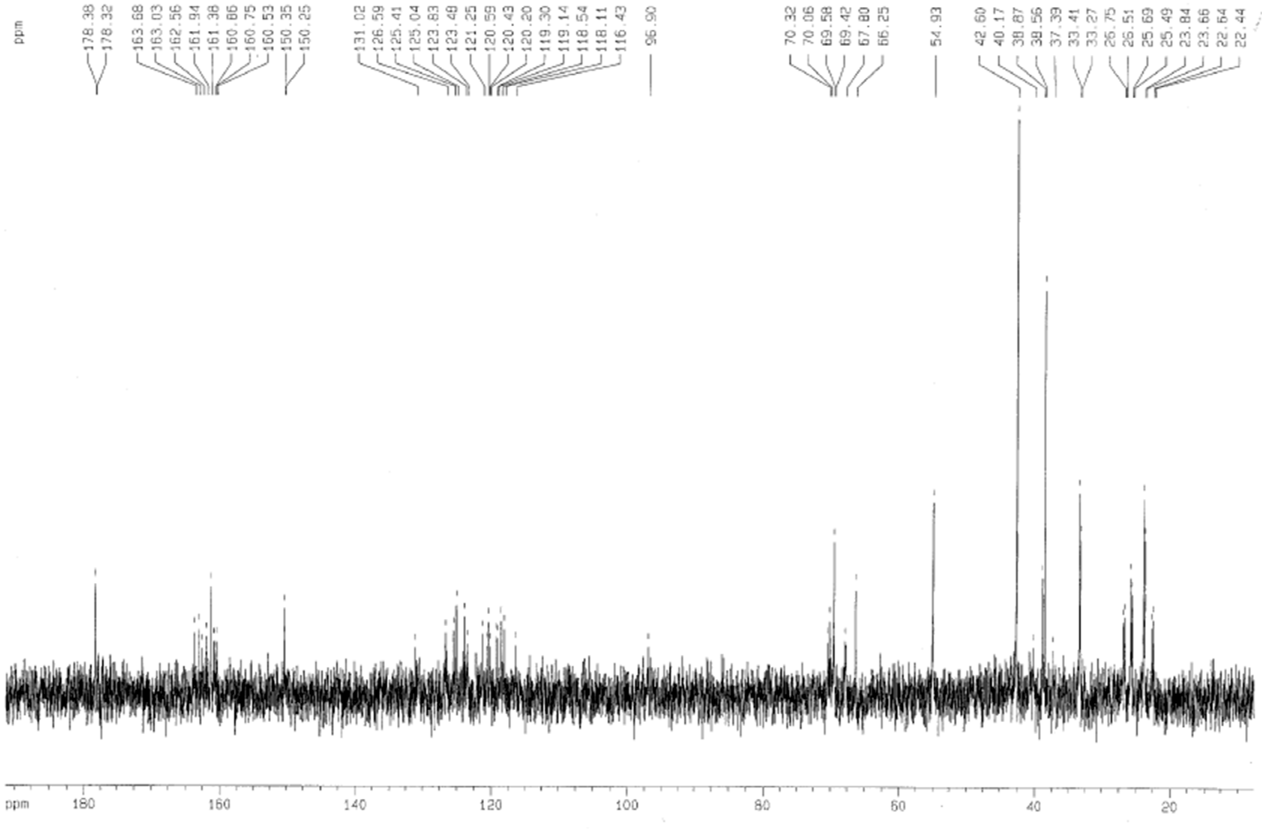

NDI-11•2CF$_3$COOH. ^{1}H-NMR (400 MHz, DMSO-d$_6$)

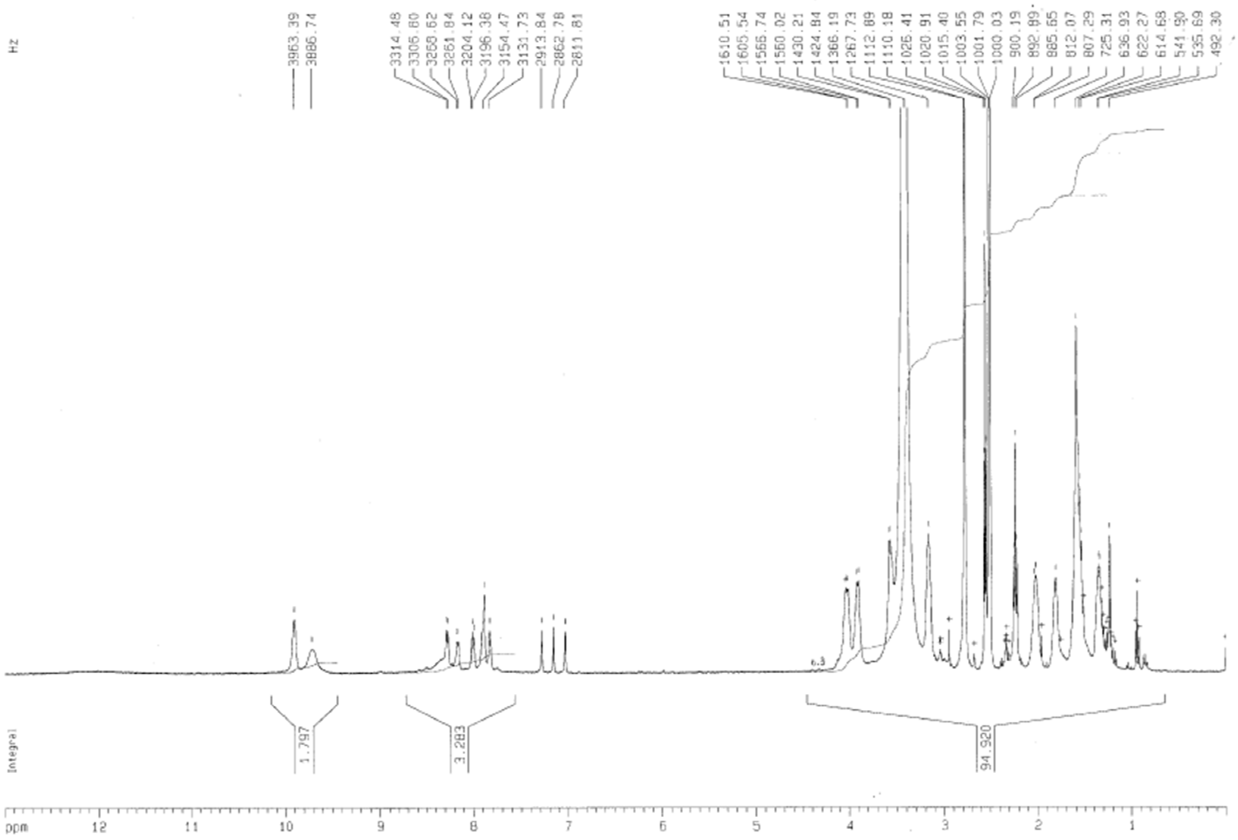

NDI-11•2CF$_3$COOH. ^{13}C-NMR (100 MHz, DMSO-d$_6$)

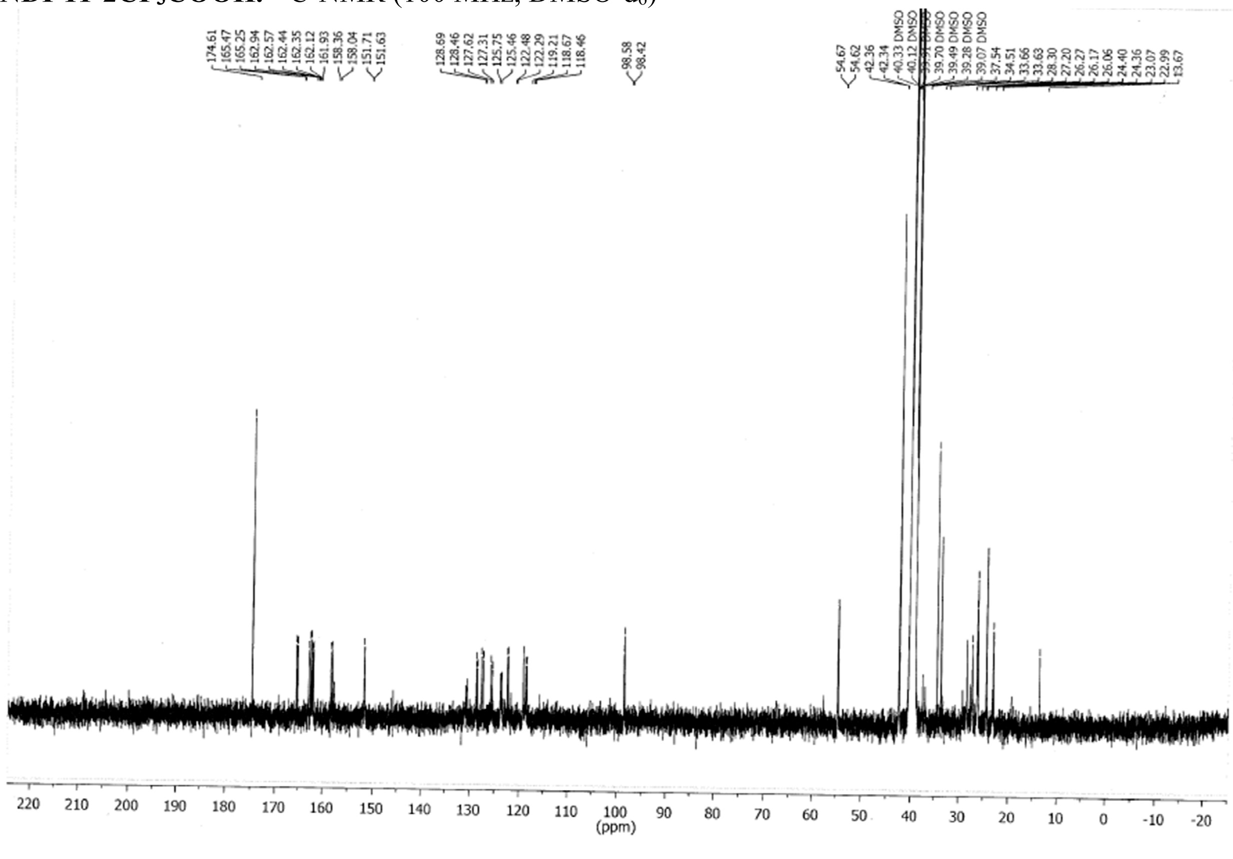

NDI-12•2CF$_3$COOH. ^{1}H-NMR (400 MHz, DMSO-d$_6$)

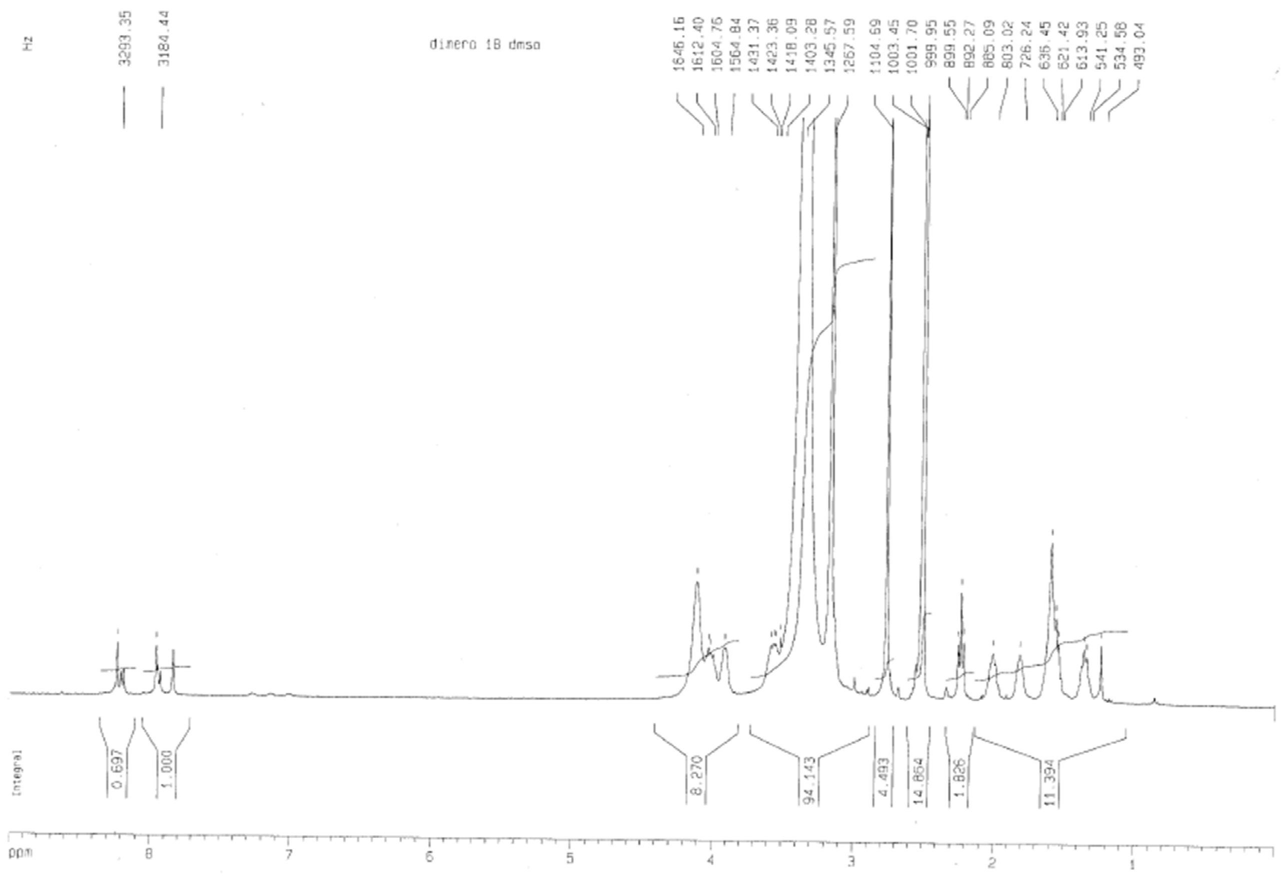

NDI-12•2CF$_3$COOH. ^{13}C-NMR (400 MHz, DMSO-d$_6$)

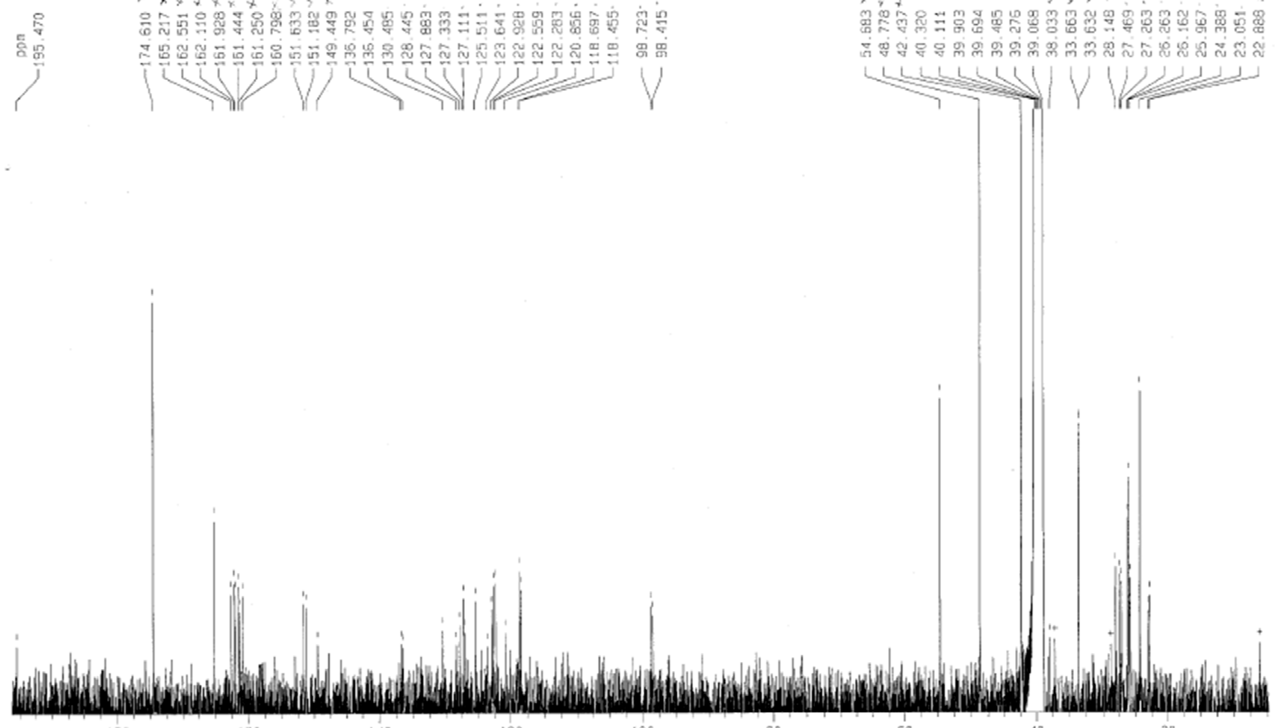

NDI-6•3CF$_3$COOH. ^{1}H-NMR (300 MHz, D_2O)

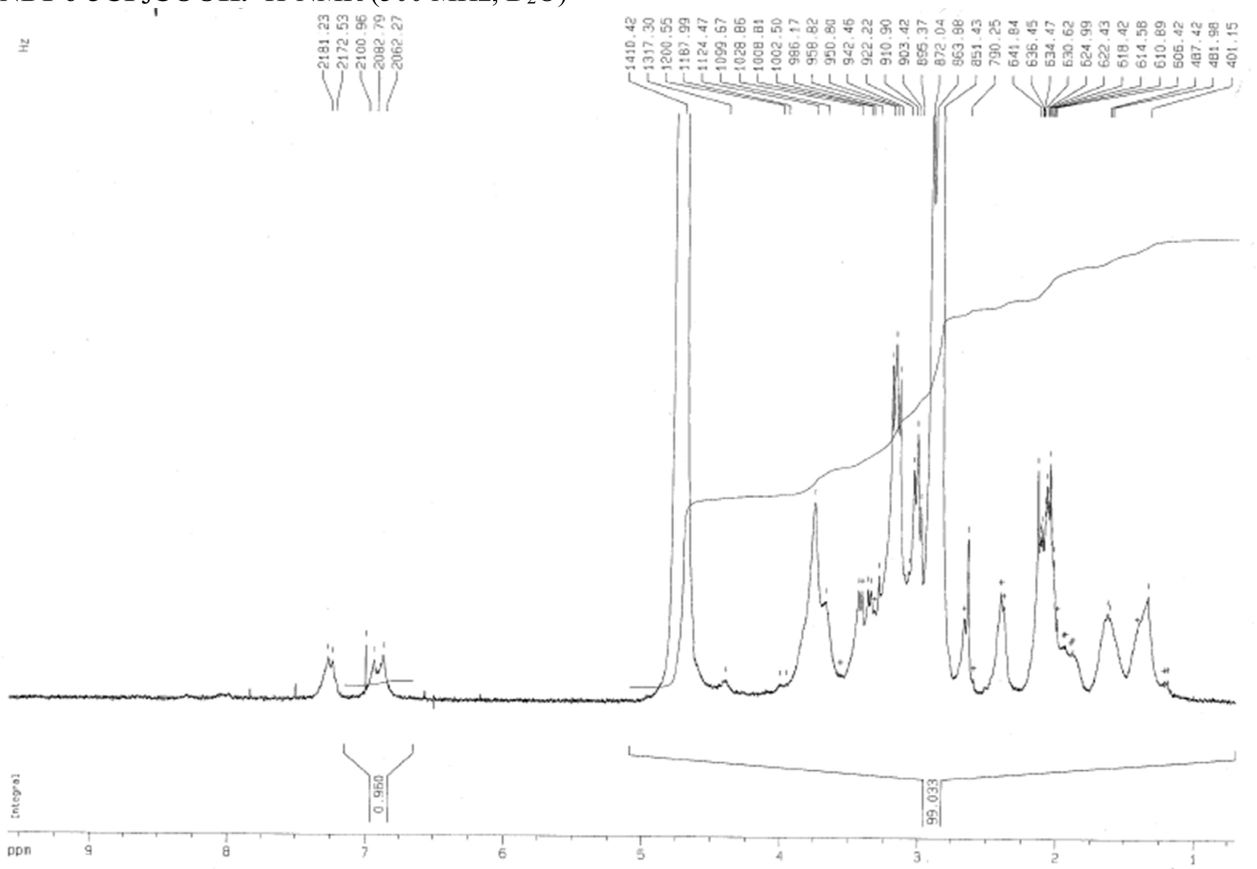

NDI-6•3CF$_3$COOH. ^{13}C-NMR (75 MHz, D_2O)

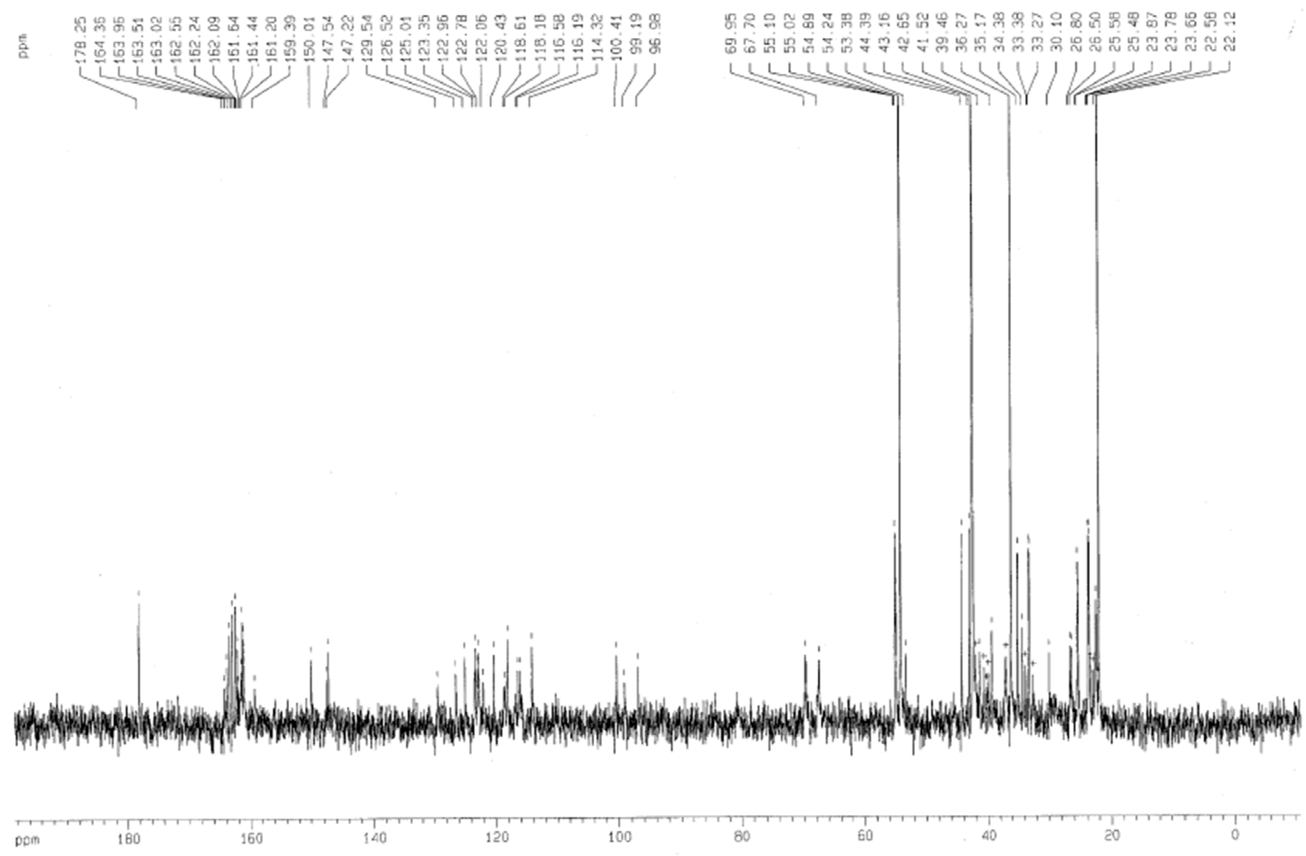

Supplement: Supplementary file 1 [file ijms-21-01964-s001.pdf]
